# Within-Person Variability Score-Based Causal Inference: A Two-Step Estimation for Joint Effects of Time-Varying Treatments

**DOI:** 10.1007/s11336-022-09879-1

**Published:** 2022-08-18

**Authors:** Satoshi Usami

**Affiliations:** Department of Education, University of Tokyo, Tokyo, Japan

**Keywords:** longitudinal data, observational study, causal inference, marginal structural model, structural nested mean model

## Abstract

Behavioral science researchers have shown strong interest in disaggregating within-person relations from between-person differences (stable traits) using longitudinal data. In this paper, we propose a method of within-person variability score-based causal inference for estimating joint effects of time-varying continuous treatments by controlling for stable traits of persons. After explaining the assumed data-generating process and providing formal definitions of stable trait factors, within-person variability scores, and joint effects of time-varying treatments at the within-person level, we introduce the proposed method, which consists of a two-step analysis. Within-person variability scores for each person, which are disaggregated from stable traits of that person, are first calculated using weights based on a best linear correlation preserving predictor through structural equation modeling (SEM). Causal parameters are then estimated via a potential outcome approach, either marginal structural models (MSMs) or structural nested mean models (SNMMs), using calculated within-person variability scores. Unlike the approach that relies entirely on SEM, the present method does not assume linearity for observed time-varying confounders at the within-person level. We emphasize the use of SNMMs with G-estimation because of its property of being doubly robust to model misspecifications in how observed time-varying confounders are functionally related to treatments/predictors and outcomes at the within-person level. Through simulation, we show that the proposed method can recover causal parameters well and that causal estimates might be severely biased if one does not properly account for stable traits. An empirical application using data regarding sleep habits and mental health status from the Tokyo Teen Cohort study is also provided.

**Supplementary Information:**

The online version contains supplementary material available at 10.1007/s11336-022-09879-1.

## Introduction

Estimating the causal effects of (a sequence of) time-varying treatments/predictors on outcomes is a challenging issue in longitudinal observational studies because researchers must account for time-varying and time-invariant confounders. For this analytic purpose, potential outcome approaches such as marginal structural models (MSMs; Robins, 1999; Robins et al., 2000) have been widely used in epidemiology. Although actual applications have been relatively infrequent, structural nested models (SNMs; Robins, 1989, 1992) with G-estimation are in principle more suitable and robust for handling violation of the usual assumptions of no unobserved confounders and sequential ignorability (Robins, 1999; Robins & Hernán, 2009; Vansteelandt & Joffe, 2014).

Parallel with such methodological development, behavioral science researchers have shown interest in inferring within-person relations in longitudinally observed variables, namely, how changes in one variable influence another for the same person. Investigations based on within-person relations might produce conclusions opposite to those based on between-person relations. For example, a person is more likely to have a heart attack during exercise (within-person relation), despite people who exercise more having a lower risk of heart attack (between-person relation; Curran & Bauer, 2011).

Statistical inference for disaggregating within- and between-person (or within- and between-group) relations has been a concern in behavioral sciences for more than half a century. However, recent methodological development and extensive discussion (Cole et al., 2005; Hamaker, 2012; Hamaker et al., 2015; Hoffman, 2014; Usami et al., 2019a) have rapidly increased interest in this topic. In the psychometrics literature, along with multilevel modeling (e.g., Wang & Maxwell, 2015), structural equation modeling (SEM)-based approaches have become one popular method for uncovering within-person relations. Among these approaches, applications of a random-intercept cross-lagged panel model (RI-CLPM; Hamaker et al., 2015), which includes common factors called *stable trait factors*, have rapidly increased, reaching more than 1500 citations on Google as of June 2022. This model was originally proposed to uncover reciprocal relations among focal variables that arise at the within-person level (i.e., simultaneous investigations for the effects of a variable *X* on a variable *Y*, along with the effects of *Y* on *X*), without explicit inclusion of (time-varying) observed confounders *L* (however, Mulder & Hamaker, 2021) discussed an extension that included a between-level predictor).

Despite its popularity and theoretical appeal, the concepts of stable traits and within-person relations in the RI-CLPM have not been fully characterized in the causal inference literature. This might be partly because psychometricians have used these terms vaguely and ambiguously in statistical models, without clarifying the assumed data-generating process (DGP) and providing clear mathematical definitions. For this reason, the RI-CLPM has not been contrasted with many other methodologies used for causal inference (e.g., MSMs and SNMs). One potential advantage of the RI-CLPM as SEM is that it can easily include and estimate measurement errors in statistical models under parametric assumptions. However, the RI-CLPM demands linear regressions at the within-person level that are correctly specified to link focal variables (as well as time-varying observed confounders, if included in the model). The linearity assumption typically imposed with respect to time-varying observed confounders in path modeling and SEM has often been criticized in the causal inference literature (e.g., Hong, 2015), and relaxing this assumption is often a key to consistently estimating the causal quantity of interest (e.g., Imai & Kim, 2019).

In this paper, we propose a method of *within-person variability score*-based causal inference for estimating joint effects of time-varying continuous treatments/predictors at the within-person level by controlling for stable traits (i.e., between-person differences), which are assumed to be uncorrelated with within-person relations as in the RI-CLPM. The proposed method is a two-step analysis. A within-person variability score for each person, which is disaggregated from the stable trait factor score of that person, is first calculated using weights based on a best linear correlation preserving predictor through SEM. Causal parameters are then estimated by MSMs or SNMs, using calculated within-person variability scores. The proposed method still requires specification of the structure for within-person variability scores over time for each variable (e.g., *Y* and *L*) in the first step. However, this approach is more flexible than the one that relies entirely on SEM (e.g., the RI-CLPM that includes time-varying observed confounders) in terms of modeling how time-varying observed confounders are functionally related to treatments/predictors and outcomes at the within-person level, without imposing the linearity assumption in these relations. We particularly emphasize the utility of SNMs with G-estimation because of its attractive property of being doubly robust to model misspecifications in how time-varying observed confounders are functionally related to treatments/predictors and outcomes at the within-person level.

The proposed method can be viewed as one that synthesizes two traditions for factor analysis methods and SEM in psychometrics and a method of causal inference (MSMs or SNMs) in epidemiology. Because causal estimands that are defined at the within-person level are less common in the causal inference literature (Lüdtke & Robitzsch, 2021), the proposed method offers new insights for researchers in a broad range of disciplines who are interested in causal inference. Also, the idea of using within-person variability scores can be applied to many other issues that are closely relevant to causal hypotheses, including reciprocal effects and mediation effects.

The remainder of this paper is organized as follows. Because the concepts of stable traits and within-person relations have not been fully characterized in the causal inference literature, in Sect. 2 we start our discussion by introducing the two different DGPs in which time-invariant factors are included. After providing formal definitions of stable trait factors (for between-person relations) and within-person variability scores (for within-person relations), the definition of joint effects of time-varying treatments at the within-person level and their identification conditions are described in Sect. 3. We then introduce the proposed methodology in Sect. 4. In Sect. 5, we perform simulations and show that the proposed method can recover causal parameters well, and that causal estimates might be severely biased if stable traits are not properly accounted for. Section 6 describes an empirical application of the proposed method using data from the Tokyo Teen Cohort (TTC) study (Ando et al., 2019). The final section gives some concluding remarks and discusses our future research agenda.

## Causal Models and Data-Generating Processes

In this section, we first explain two different DGPs and causal models in which time-invariant factors are included. In the first DGP, we assume that time-invariant factors have both direct and indirect effects on measurements; recent work by Gische et al. (2021), which provided a didactic presentation of the directed acyclic graph (DAG)-based approach and key concepts regarding causal inference based on a cross-lagged panel design, assumed this process. In the second DGP, we assume that time-invariant factors have only direct effects; this corresponds to the process that researchers (implicitly) assume in applying the RI-CLPM to infer within-person relations. This distinction of processes is inspired by Usami et al. (2019a), who highlighted how common factors included in the different statistical models to examine reciprocal relations have different conceptual and mathematical properties.

Below, we suppose that data are generated at fixed time points $t_0,t_1,\dots$,$t_{K}$. Let $A_{ik}$ denote a continuous treatment/predictor at time $t_k$ ($k=0,\dots ,K-1$) for person *i*, and let $L_{ik}$ denote time-varying observed confounders at that time for person *i*.^1^ Furthermore, $Y_{ik}$ is the outcome at time $t_k$ ($k=0,\dots ,K$) for person *i* and is part of the time-varying confounders $L_{ik}$. Suppose that a time-varying confounder has three characteristics: it is independently associated with future outcomes $Y_{ik'}$, it predicts subsequent levels of treatment as well as future confounders, and it is affected by an earlier treatment and confounders (Vansteelandt & Joffe, 2014). In this paper, for the purpose of explanation, we assume a single confounder that is measured concurrently with the outcome at each time point and is measured before the treatment/predictor level is determined for each person. Thus, we presume that the variables are ordered as $L_0,A_0,L_1,A_1,\dots ,L_{K-1},A_{K-1},L_K$.^2^

### Data-Generating Process 1: Time-Invariant Factors Have Both Direct and Indirect Effects on Measurements

**Fig. 1:**
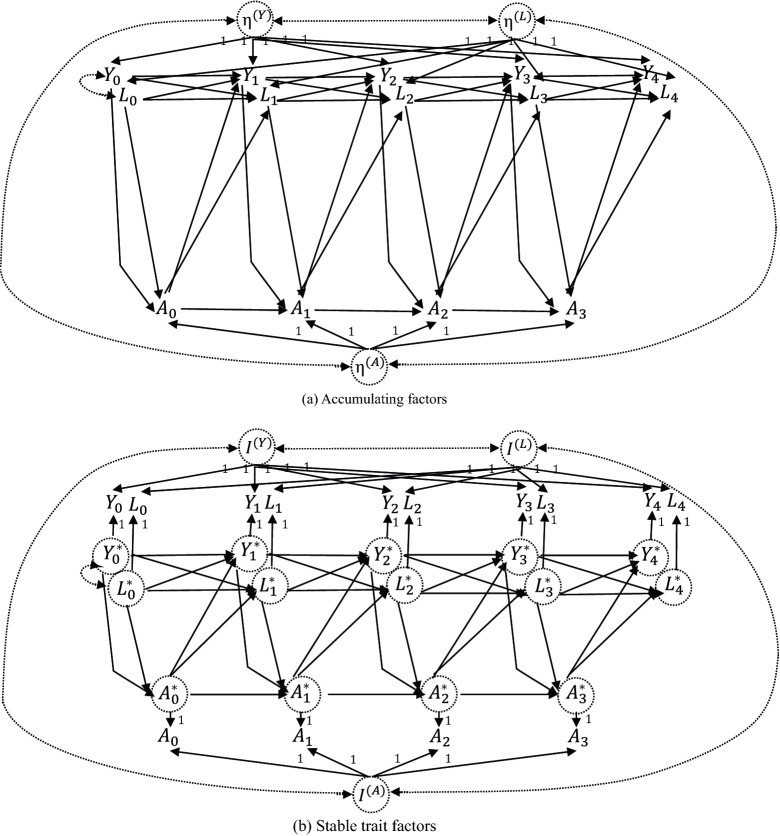
The linear causal diagrams (DAGs) for two different data-generating processes in which time-invariant factors are included. Solid single-headed arrows (directed edges) are labeled with path coefficients that quantify direct causal effects. A dashed double-headed arrow (bidirected edge) represents a correlation due to an unobserved common cause. Time-invariant factors are represented in dashed circles, indicating that these are latent variables. For explanatory purposes, it is temporarily assumed that the process does not start prior to the initial measurement. **a** Time-invariant factors ($\eta$: called accumulating factors) have both direct and indirect effects on measurements. **b** Time-invariant factors (*I*: called stable trait factors) have only direct effects on measurements.

Gische et al. (2021) introduced the DAG-based approach to causal inference, explaining how (SEM-based) statistical models can identify the causal models. Figure 1a is a DAG that expresses linear causal relations among variables in $K=4$; this is similar to the one presented by Gische et al. (2021) but with time-varying observed confounders *L* now included. Each solid single-headed arrow represents a direct causal relation, and a dashed double-headed arrow indicates the existence of an unobserved confounder. Dashed circles are used to express latent variables. To keep the illustration simple, here we assume (i) first-order (linear) lagged effects of variables and (ii) that mechanisms that are not directly targeted by treatment are not altered (modularity). More importantly, for the purpose of illustration, we temporarily assume that the process does *not* start prior to the initial measurement ($k=-1,-2,\dots$), indicating that initial measurements are the beginning of the process. Thus, time-invariant factors $\eta$ as random intercepts do *not* have direct causal effects on the dynamics among variables that might be going on prior to the initial measurement. We will revisit this issue later.

In this linear causal DAG model, we suppose that time-invariant factors $\eta$ are additive to express person-specific differences in the mean levels of the respective variables (*Y*, *A*, and *L*), which do not change over time. Without loss of generality, we assume that these factors have zero means ($E(\eta ^{(Y)})=E(\eta ^{(A)})=E(\eta ^{(L)})=0$). If we are interested in the longitudinal change of sleep time in adolescents, as will be investigated in the later empirical example, then $\eta$ reflects all time-invariant factors that might affect the level of sleep time in an adolescent during the course of study (e.g., sex, year of birth, constitution, genetic endowment, health, exercise habits, home environment including discipline, engagement in club/extracurricular activities in school). The values of coefficients corresponding to the paths from time-invariant factors to measurements are restricted to be equal to one. These restrictions (a) assign a scale to the latent random intercept and (b) reflect the assumption that the structural coefficients from the random intercepts to measurements do not change over time (Gische et al., 2021). The bidirected dashed edges between time-invariant factors indicate that they might show covarying relations due to unobserved confounding. Likewise, the bidirected dashed edge between initial measurements $Y_0$ and $L_0$ indicates that they might show covarying relations due to unobserved confounding.

Under this linear causal DAG model, the DGP can be represented by the following set of linear equations ($k \ge 1$):

1 $$\begin{aligned} Y_{ik}&=\eta ^{(Y)}_{i}+\mu ^{(Y)}_{k}+\alpha ^{(Y)}_{k}Y_{i(k-1)}+\beta ^{(Y)}_{k}A_{i(k-1)}+\gamma ^{(Y)}_{k}L_{i(k-1)}+d^{(Y)}_{ik}, \nonumber \\ A_{ik}&=\eta ^{(A)}_{i}+\mu ^{(A)}_{k}+\alpha ^{(A)}_{k}Y_{ik}+\beta ^{(A)}_{k}A_{i(k-1)}+\gamma ^{(A)}_{k}L_{ik}+d^{(A)}_{ik}, \nonumber \\ L_{ik}&=\eta ^{(L)}_{i}+\mu ^{(L)}_{k}+\alpha ^{(L)}_{k}Y_{i(k-1)}+\beta ^{(L)}_{k}A_{i(k-1)}+\gamma ^{(L)}_{k}L_{i(k-1)}+d^{(L)}_{ik}. \end{aligned}$$

Here, $\mu ^{(Y)}_{k}$, $\mu ^{(A)}_{k}$, and $\mu ^{(L)}_{k}$ are (fixed) intercepts at time $t_k$ and are omitted in the DAG representation. Residual terms are denoted by *d* and are assumed to be uncorrelated with time-invariant factors; they are also usually omitted in the DAG representation. It is also assumed that there is no unobserved common cause among these residuals (i.e., concurrent residuals are mutually uncorrelated). Note that the equations assume homogeneity: the coefficients $\alpha , \beta$, and $\gamma$ are fixed and constant across persons.

As suggested by Gische et al. (2021), a statistical model that captures the DAG depicted in Fig. 1a (i.e., Eq. 1) can be globally identified: all parameters can theoretically be estimated uniquely from observational data. Assuming sufficient sample size, correct model specification, and no excess multivariate kurtosis, the SEM-based maximum likelihood (ML) method provides estimates that are asymptotically unbiased, efficient, and consistent (Bollen, 1989). More details about causal identification and estimation in linearly parameterized causal DAG models are provided by Gische and Voelkle (in press).

A notable feature of this DGP is that time-invariant factors have both direct and indirect effects on measurements. For example, $\eta ^{(Y)}$ has a direct effect on $Y_3$ (i.e., $\eta ^{(Y)}\rightarrow Y_3$), while $Y_3$ is also caused by $Y_2$, which is again caused by $\eta ^{(Y)}$ (i.e., $\eta ^{(Y)}\rightarrow Y_2\rightarrow Y_3$). In addition, other time-invariant factors $\eta ^{(A)}$ and $\eta ^{(L)}$ also have indirect effects on $Y_3$ (e.g., $\eta ^{(A)}\rightarrow A_2\rightarrow Y_3$). These indirect effects result from the fact that time-invariant factors are modeled with lagged regressions in Fig. 1a (or Eq. 1), rather than being modeled separately.

Usami et al. (2019a) compared several existing statistical models to examine reciprocal relations among variables, emphasizing that whether or not common factors are modeled with lagged regression makes substantial differences in the conceptual and mathematical roles of common factors. For example, the common factors included in the latent change score model (LCS; McArdle & Hamagami, 2001), autoregressive latent trajectory model (ALT; Bollen & Curran, 2004), and general cross-lagged panel model (GCLM; Zyphur et al., 2020a; b) and individual-specific effects that are often included in longitudinal panel models of econometrics (e.g., dynamic panel model) are commonly modeled with lagged regressions, reflecting that they have both direct and indirect effects on measurements. This type of common factor is called an *accumulating factor* (Usami, 2021; Usami et al., 2019a) because its effects accumulate in measurements at later time points through the lagged regression. However, in the RI-CLPM (that includes time-varying observed confounders), which researchers are increasingly using to uncover within-person relations, common factors (i.e., stable trait factors) are *not* modeled with lagged regression, indicating that this statistical model cannot identify parameters in the causal DAG model as depicted in Fig. 1a (i.e., Eq. 1).

### Data-Generating Process 2: Time-Invariant Factors Have Only Direct Effects on Measurements

To clarify this point, let us consider a different (linear and first-order) DGP in which time-invariant factors are included but have only direct effects on measurements. In Fig. 1b, directed edges from time-invariant factors *I* are drawn to the corresponding measurements. Also, directed edges from time-varying factors, which are expressed by writing the variable name with an asterisk (e.g., $Y_3^*$), are drawn to the corresponding measurements. Directed edges are assumed between these time-varying factors, rather than between measurements as in Fig. 1a. Time-varying factors are also assumed to be uncorrelated with time-invariant factors. As a result, time-invariant factors *I* have only direct effects on measurements, and under the linearity assumption each measurement can be decomposed into the linear sum of time-invariant and time-varying factors that are mutually uncorrelated.

The values of coefficients corresponding to the paths from time-varying factors to measurements are all restricted to be one, and we assume that these time-varying factors have zero means. Under this linear causal DAG model, the DGP can be represented by the following linear equations (with the assumption of homogeneity of coefficients among persons) that have two major parts:

2 $$\begin{aligned} Y_{ik}=\mu ^{(Y)}_{k}+I^{(Y)}_{i}+Y^*_{ik},\,A_{ik}=\mu ^{(A)}_{k}+I^{(A)}_{i}+A^*_{ik},\,L_{ik}=\mu ^{(L)}_{k}+I^{(L)}_{i}+L^*_{ik} \end{aligned}$$

for $$k\ge 0$$, and

3 $$\begin{aligned} Y^*_{ik}&=\alpha ^{(Y)}_{k}Y^*_{i(k-1)}+\beta ^{(Y)}_{k}A^*_{i(k-1)}+\gamma ^{(Y)}_{k}L^*_{i(k-1)}+d^{(Y)}_{ik}, \nonumber \\ A^*_{ik}&=\alpha ^{(A)}_{k}Y^*_{ik}+\beta ^{(A)}_{k}A^*_{i(k-1)}+\gamma ^{(A)}_{k}L^*_{ik}+d^{(A)}_{ik}, \nonumber \\ L^*_{ik}&=\alpha ^{(L)}_{k}Y^*_{i(k-1)}+\beta ^{(L)}_{k}A^*_{i(k-1)}+\gamma ^{(L)}_{k}L^*_{i(k-1)}+d^{(L)}_{ik} \end{aligned}$$

for $k \ge 1$. $\mu ^{(Y)}_{k}$, $\mu ^{(A)}_{k}$, and $\mu ^{(L)}_{k}$ are the temporal group means (rather than fixed intercepts) at time point $t_k$ and are omitted in the DAG representation. The residual terms *d* are assumed to be uncorrelated with both time-invariant and time-varying factors and are also omitted in the DAG representation. As suggested from Eq. (2), under these specifications the time-varying factors $Y^*_{ik}$, $A^*_{ik}$, and $L^*_{ik}$ represent temporal deviations from the expected score for person *i* at time point $t_k$ (i.e., $\mu ^{(Y)}_{k}+I^{(Y)}_{i}$, $\mu ^{(A)}_{k}+I^{(A)}_{i}$, and $\mu ^{(L)}_{k}+I^{(L)}_{i}$), whereas time-invariant factors represent stable between-person differences over time. The time series $Y^*_{ik}$, $A^*_{ik}$, and $L^*_{ik}$ can thus be interpreted as within-person variations that are uncorrelated from time-invariant factors as stable between-person differences.

In psychology, *traits* were originally considered as personality characteristics that are stable over time and in different situations. To express such latent constructs, common factors are explicitly included in psychometric models. In the context of the RI-CLPM, such common factors are called *stable trait factors*, and they have the same role as that of time-invariant factors *I* in the linear causal DAG model as depicted in Fig. 1b (i.e., Eq. 2). In the RI-CLPM, the initial deviations are modeled as exogenous variables, and their variances and covariances are estimated. Residuals in this statistical model are usually assumed to follow a multivariate normal distribution. Although the original motivation for the RI-CLPM was to infer reciprocal (rather than unidirectional) relations and the model does not usually assume time-varying observed confounders *L* and higher-order lagged effects of variables, it can be extended in a straightforward manner to investigate (joint) effects of continuous treatments/predictors *A* on outcomes *Y*, while including *L* in linear regressions. Therefore, such an extended version of the RI-CLPM as a statistical model can identify the causal parameters if the assumed DGP as in Fig. 1b (i.e., Eqs. 2 and 3) is correct and if $K \ge 2$ (i.e., three or more time points; Usami et al., 2019a).

Usami et al. (2019a) explained that the conceptual and mathematical roles of common factors differ according to whether or not they are modeled with lagged regression in the statistical model. More specifically, in models that include accumulating factors, their influences on measurements at time $t_k$ (e.g., $\eta ^{(Y)}\rightarrow Y_k$) transmit to the future measurements (e.g., $Y_k \rightarrow Y_{k+1}$) through the lagged regression, which is also influenced by the same accumulating factors (e.g., $\eta ^{(Y)}\rightarrow Y_{k+1}$); as a result, the magnitudes of impacts from these factors change over time. In contrast, in models that include stable trait factors (e.g., the RI-CLPM), their impacts are stable over time because they have only direct effects. In this way, the conceptual meaning and inferential results for (within-person) relations among variables being modeled differ in each statistical model according to whether researchers assume the inclusion of accumulating factors or stable trait factors; see Usami et al. (2019a, pp. 643–644) for a more detailed comparison.

### Implications of Comparing Different DGPs: Control for Time-Invariant Unobserved Confounders and Initial Conditions

#### Control for Time-Invariant Unobserved Confounders

As we have argued, the conceptual and mathematical roles differ between stable trait factors and accumulating factors. Importantly, the differences between these factors can also be characterized as whether or not they can be considered as time-invariant unobserved confounders. For example, the accumulating factors of outcomes ($\eta ^{(Y)}$) cause measurements $Y_k$ ($k \ge 1$) while also being associated with measurements of treatments/predictor at the previous time point ($A_{k-1}$). In this sense, accumulating factors can be considered as unobserved confounders in evaluating causal effects of treatments/predictors. In contrast, the stable trait factors of outcomes ($I^{(Y)}$) also cause measurements $Y_k$ but are uncorrelated with within-person variations such as $Y^*_{k}$ and $A^*_{k-1}$. More specifically, when measurements $Y_k$ are unconditional, $I^{(Y)}$ does not confound the relations among within-person variations (e.g., the path from $A^*_{k-1}$ to $Y^*_{k}$) because the path from $I^{(Y)}$ to within-person variations $Y^*_k$ is blocked by the measurement $Y_k$, which act as colliders. Therefore, stable trait factors cannot be viewed as time-invariant unobserved confounders; rather, they should be characterized as merely random intercepts that are uncorrelated with predictors (i.e., within-person variations). This view differs from that of Usami et al. (2019a), who explain stable trait factors as time-invariant unobserved confounders.

If the assumed (linear and first-order) DGP as in Fig. 1b is correct and if all variables are observable, then controlling for only $Y^*_{k-1}$ and $L^*_{k-1}$ is sufficient to evaluate the within-person relation between $A^*_{k-1}$ and $Y^*_{k}$. One could argue that controlling for stable trait factors is not required to identify causal parameters for treatment effects at the within-person level, the reason being that within-person processes (time-varying factors) and between-person differences (stable trait factors as time-invariant factors) are mutually uncorrelated. However, because all these factors are actually latent variables and unobservable, we need to use measurements (as colliders) to infer treatment effects at the within-person level, and appropriate control of stable trait factors as latent variables is required in the statistical model. If the assumed DGP as in Fig. 1b is correct, then not controlling for stable trait factors causes biased estimates of causal parameters for the within-person relation (e.g., Usami et al., 2019b and the later simulations).

#### Initial Conditions

How to treat the initial measurements (i.e., $Y_0$, $L_0$, and $A_0$) is also important for distinguishing between the two DGPs in Fig. 1. Special attention needs to be paid to the initial measurements because there are no incoming directed edges to these variables from variables prior to the initial time point ($k=-1,-2,\dots$). Although we have assumed so far that the DGP does not start prior to the initial measurements, this assumption is not realistic in many applications, and the initial measurements must somehow account for the past of the process that is not explicitly modeled (see Fig. S1 in the Online Supplemental Material for more details). Assuming a DGP similar to that in Fig. 1a, Gische et al. (2021, Fig. 5) provided a straightforward and interpretable approach that freely estimates the coefficients (loading) from $\eta$ to *all* initial measurements. For example, for $\eta ^{(Y)}$, the coefficients from this factor to $Y_0$, $L_0$, and $A_0$ are freely specified rather than being fixed to either one or zero. In applying dynamic panel models in econometrics, one usually assumes that individual-specific components (i.e., accumulating factors) are correlated with the initial measurements to account for the past of the process.

Importantly, if the second DGP (Fig. 1b) is correct, then such special considerations are not required. This is because time-invariant factors *I* have only direct effects on measurements (rather than on temporal deviations as within-person variations) and no directed edges are assumed between observed variables. In other words, past time-varying factors ($Y_{-1}^*, Y_{-2}^*,\dots$, $A_{-1}^*, A_{-2}^*,\dots$, $L_{-1}^*, L_{-2}^*,\dots$) as variations in within-person processes cause observed variables separately from *I* as stable between-person differences (see also Fig. S1 in the Online Supplemental Material). Therefore, if the assumed DGP as depicted in Fig. 1b is correct, then the RI-CLPM, which includes time-varying observed confounders and assumes that initial variables at the within-person level ($Y_0^*$, $L_0^*$) are exogenous (and are mutually correlated) and that loadings from *I* to the corresponding initial measurements (i.e., $I^{(Y)}\rightarrow Y_0$, $I^{(A)}\rightarrow A_0$ and $I^{(L)}\rightarrow L_0$) are all set to one, can identify causal parameters for treatment effects, even if the DGP actually starts prior to the initial measurements.

### Summary and Discussion

The critical difference between the two different DGPs in Fig. 1 is whether the assumed time-invariant factors have only (stable) direct effects (i.e., stable trait factors) or both direct and indirect effects on measurements (i.e., accumulating factors). Because of this difference, stable trait factors as merely random intercepts cannot be viewed as time-invariant unobserved confounders, while special considerations for initial measurements are not required if the assumed DGP includes only stable trait factors (i.e., Fig. 1b). Although researchers are increasingly using the RI-CLPM as a statistical model to uncover within-person relations, stable traits and (implicitly) assumed DGPs have not been fully characterized in the causal inference literature. Below, we assume a DGP that includes stable trait factors as in Fig. 1b and also assume that measurements can be decomposed into the linear sum of time-invariant factors (i.e., stable traits) and time-varying factors (i.e., within-person variability scores) that are mutually uncorrelated. The proposed method of *within-person variability score*-based causal inference for (joint) effects of time-varying treatments at the within-person level can be effectively applied if such a DGP can be assumed. The proposed approach is more flexible than the one that relies entirely on SEM (e.g., the RI-CLPM that includes time-varying observed confounders) in terms of the linearity assumption regarding observed confounders at the within-person level.

A causal DAG represents a researcher’s theory about the causal process and should be drawn based on subject-matter knowledge. However, in many cases, researchers do not exactly know the true DGP and how time-invariant factors (if they exist) influence measurements (e.g., linearly or nonlinearly, directly or indirectly, or both). Although it is ideal if one can unambiguously articulate the theoretically derived expected relations for variables, this can be quite challenging in practical applications (Curran & Bauer, 2011). If linear SEM-based statistical models are used, then as a data-driven approach one could compare model fit indices between two statistical models that appropriately represent the causal models of Eq. (1) and Eqs. (2) and (3) (i.e., the RI-CLPM that includes time-varying observed confounders), and this would be useful for investigating the sensitivity of the conclusions.

In the context of applying the RI-CLPM, Lüdtke and Robitzsch (2021) argued that including stable trait factors might be better suited for short-term studies that typically use shorter time lags between time points. In short-term studies, one might be more certain that there are no indirect effects from time-invariant factors (i.e., only stable trait factors exist). Even if a researcher is certain that stable trait factors exist, they might not be additive and/or might be correlated with within-person variability scores if they share common causes (e.g., genotype; see also McNeish and Kelly Muthén and Asparouhov (2019), who discussed the issue of endogeneity in applying mixed-effects models). This indicates that the model fails to perfectly disentangle the within- and between-person relations. Also, if time-invariant unobserved confounders (rather than random intercepts that merely represent between-person differences as stable trait factors) are likely to be present, then other statistical approaches that account for such confounders might be more suitable. However, in our opinion there are no clear criteria that delineate when and how to include (time-invariant) factors in the assumed DGP, and continued discussion that also considers empirical investigations of each research hypothesis and sensitivity of results (e.g., the later simulations) will be required in the future.

## Formal Definitions of Stable Trait Factors, Within-Person Variability Scores and Joint Effects of Time-Varying Treatments

### Definitions of Stable Trait Factors and Within-Person Variability Scores

The terms “(stable) traits” and “within-person relations” have been used vaguely and ambiguously in statistical models, despite the existence of mathematical and interpretative differences among models (e.g., Usami et al., 2019a). Inspired by the discussion so far, we provide the formal definitions of these below.

A *stable trait factor* of person *i* (say, for *Y*) is defined in this paper as (i) the time-invariant factor that has additive influence on measurements, and (ii) its quantity is equal to the difference between the expected value of measurement (i.e., true score) of this person at time point $t_k$ (expressed as $T^{(Y)}_{ik}$) and the temporal group mean ($\mu ^{(Y)}_{k}$), which is invariant over time:

4 $$\begin{aligned} I_{i}^{(Y)}=T^{(Y)}_{ik}-\mu _{k}^{(Y)} \end{aligned}$$

for $k=0,\dots ,K$, $-\infty< {T}_{ik}^{(Y)}< \infty$, and $-\infty< \mu _{k}^{(Y)}< \infty$. Note that $E(I_{i}^{(Y)})=E({T}^{(Y)}_{ik}-\mu _{k}^{(Y)})=\mu _{k}^{(Y)}-\mu _{k}^{(Y)}=0$.

Next, the *within-person variability score* $Y^*_{ik}$ is defined as (i) a time-varying factor that has additive influence on measurements, and (ii) its quantity is equal to the difference between a measurement and its expected value:

5 $$\begin{aligned} Y^*_{ik}=Y_{ik}-{T}^{(Y)}_{ik}=Y_{ik}-(\mu _{k}^{(Y)}+I_{i}^{(Y)}), \end{aligned}$$

with the assumptions of $E(Y^*_{ik})=0$ and independence between ${T}^{(Y)}_{ik}$ and $Y^*_{ik}$. From this formulation, stable trait factors and within-person variability scores are uncorrelated because

6 $$\begin{aligned} \text {Cov}(I_{i}^{(Y)},Y^*_{ik})=E[(T^{(Y)}_{ik}-\mu _{k}^{(Y)})Y^*_{ik}] =E({T}^{(Y)}_{ik}Y^*_{ik})-\mu _{k}^{(Y)}E(Y^*_{ik})=0. \end{aligned}$$

Thus, variances of measurements at time point $t_k$ can be expressed as the sum of those of stable trait factor scores and within-person variability scores. This means that the time series for within-person variability scores have the following covariance structure:

7 $$\begin{aligned} \text {Cov}(Y^*_{ik},Y^*_{ik'})=\text {Cov}(Y_{ik},Y_{ik'})-\text {Var}(I_{i}^{(Y)}). \end{aligned}$$

In this paper, we use the terms *within-person relation* and *between-person relation* to describe the relations between variables that are based on within-person variability scores and stable trait factor scores, respectively.

### Definition of Joint Effects of Time-Varying Treatments at the Within-Person Level

Next, we explain the definition of joint (causal) effects of treatments at the within-person level using the potential outcome approach. We assume a similar causal DAG model to that in Fig. 1b: (i) measurements are expressed by the linear sum of stable trait factors and within-person variability scores, and (ii) within-person variability scores are expressed by functions (with assumption of homogeneity) of those in past time. However, unlike the presentation in Sect. 2, we relax some assumptions about the within-person variability scores to allow the following: (a) higher-order lagged effects and interaction effects of treatments/predictors can exist at the within-person level, and (b) time-varying observed confounders can be nonlinearly related with outcomes and treatments/predictors at the within-person level. The current focus is on evaluating the within-person relation between variables, that is, how the (joint) intervention of treatments/predictors influences future outcomes at the within-person level.

Below, we use overbars $\bar{Y}^*_k=\{Y^*_0,Y^*_1,\dots ,Y^*_k\}$ to denote the history of $Y^*$ through $t_k$ and underbars $\underline{Y}^*_k=\{Y^*_k,\dots ,Y^*_K\}$ to denote the future of this variable. Let ${Y^*_{ik}}^{{\bar{A}}^*_{i(k-1)}}$ ($k=1,\dots ,K$) denote the within-person variability score for the outcome that would take at time point $t_k$ for person *i* were this person to receive treatment history at the within-person level $\bar{A}^*_{i(k-1)}=\{A^*_{i0},\dots ,A^*_{i(k-1)}\}$ through $t_{k-1}$. Here, $A^*_{ik}=0$ ($k=0,\dots ,K-1$) indicates that the amount of treatments/predictors for person *i* is equal to the expected score of this person at time point $t_k$ (i.e., $A_{ik}=\mu ^{(A)}_{k}+I^{(A)}_{i}$). ${Y^*_{ik}}^{{\bar{A}}^*_{i(k-1)}}$ is a potential outcome, which we connect to the within-person variability score by the consistency assumption

8 $$\begin{aligned} Y^*_{ik}={Y^*_{ik}}^{\bar{a}^*_{i(k-1)}} \end{aligned}$$

if ${\bar{A}^*_{i(k-1)}}={\bar{a}^*_{i(k-1)}}$; otherwise, ${Y^*_{ik}}^{\bar{a}^*_{i(k-1)}}$ is counterfactual. Note that $Y^*_{ik}$ is a latent variable and unobservable, while potential outcomes for measurements (i.e., observed variables) are assumed in the standard potential outcome approach.

In potential outcome approach, causal effect refers to a contrast between potential outcomes under different treatment values. Therefore, for each causal effect, we can imagine a (hypothetical) randomized experiment to quantify it (i.e., target trial; Hernán & Robins, 2021). For example, (average) causal effect on $Y^*_{ik}$ when a continuous treatment/predictor $A^*_{i(k-1)}$ increases one unit from the reference value $a^{*r}_{i(k-1)}$ at time $t_{k-1}$ can be expressed as

9 $$\begin{aligned} E({Y^*_{ik}}^{\bar{a}^*_{i(k-2)},a^{*r}_{i(k-1)}+1}-{Y^*_{ik}}^{\bar{a}^*_{i(k-2)},a^{*r}_{i(k-1)}})=E({Y^*_{ik}}^{\bar{a}^*_{i(k-2)},a^{*r}_{i(k-1)}+1})-E({Y^*_{ik}}^{\bar{a}^*_{i(k-2)},a^{*r}_{i(k-1)}}). \end{aligned}$$

The standard assumption of no unobserved confounders or sequential ignorability indicates that

10 $$\begin{aligned} {\underline{Y}^*_{ik}}^{{\bar{a}}^*_{i(k-2)},0} \mathop {\perp \!\!\!\perp }A^*_{i(k-1)} | \bar{L}^*_{i(k-1)}, {\bar{A}^*_{i(k-2)}}=\bar{a}^*_{i(k-2)}. \end{aligned}$$

Here, $(\bar{a}^*_{i(k-2)},0)$ is the counterfactual history, that is, the history that agrees with $\bar{a}^*_{i(k-2)}$ through time $t_{k-2}$ and is zero thereafter. Along with the assumed causal DAG above as well as consistency and sequential ignorability, we impose the stable unit treatment value assumption (SUTVA; no unmodeled spillovers, e.g., Hong, 2015) and assumptions of positivity (i.e., the probability of receiving each level of treatment conditional on past confounders and treatments is greater than zero) and modularity. Under these assumptions, the average causal effect in Eq. (9) can be expressed using the difference in conditional means given information on confounders and treatment history as

11 $$\begin{aligned}&E({Y^*_{ik}}^{\bar{a}^*_{i(k-2)},a^{*r}_{i(k-1)}+1})-E({Y^*_{ik}}^{\bar{a}^*_{i(k-2)},a^{*r}_{i(k-1)}})\nonumber \\&\quad =E(Y^*_{ik}| \bar{L}^*_{i(k-1)}, {\bar{A}^*_{i(k-2)}}=\bar{a}^*_{i(k-2)}, A^*_{i(k-1)}=a^{*r}_{i(k-1)}+1)\nonumber \\&\qquad -E(Y^*_{ik}| \bar{L}^*_{i(k-1)}, {\bar{A}^*_{i(k-2)}}=\bar{a}^*_{i(k-2)}, A^*_{i(k-1)}=a^{*r}_{i(k-1)}). \end{aligned}$$

In other words, the causal effect of treatment $A^{*r}_{i(k-1)}$ at the within-person level can be evaluated by the difference in conditional means of $Y^*_{ik}$ between persons who receive $A^*_{i(k-1)}=a^{*r}_{i(k-1)}+1$ (i.e., treatment levels that are $a^{*r}_{i(k-1)}+1$ larger than their expected scores $\mu ^{(A)}_{(k-1)}+I^{(A)}_{i}$) and who receive $A^*_{i(k-1)}=a^{*r}_{i(k-1)}$, given information on confounders and treatment history.

Similarly, the average joint (causal) effects of a sequence of treatments/predictors ${{\bar{A}}^*_{i(k-1)}}$ on $Y^*_{ik}$ when they increase one unit from the reference values $\bar{a}^{*r}_{i(k-1)}$ can be expressed as

12 $$\begin{aligned}&E({Y^*_{ik}}^{\bar{a}^{*r}_{i(k-1)}+1})-E({Y^*_{ik}}^{\bar{a}^{*r}_{i(k-1)}})\nonumber \\&\quad =E(Y^*_{ik}| \bar{L}^*_{i(k-1)}, {\bar{A}^*_{i(k-1)}}=\bar{a}^*_{i(k-1)}+1)-E(Y^*_{ik}| \bar{L}^*_{i(k-1)}, {\bar{A}^*_{i(k-1)}}=\bar{a}^*_{i(k-1)}) \end{aligned}$$

As a simple example, suppose $K=2$ and that the DGP can be represented by linear and first-order models as in Eqs. (2) and (3) (assuming homogeneity and no interaction effects of treatments/predictors). Then, a conditional mean $E(Y^*_{i2}| \bar{L}^*_{i1}, \bar{A}^*_{i1}=\bar{a}^*_{i1})$ at $k=2$ can be expressed as the linear (weighted) sum of the terms $a^*_{i0}$ and $a^*_{i1}$:

13 $$\begin{aligned}&E(\alpha ^{(Y)}_{2}Y^*_{i1}+\beta ^{(Y)}_{2}a^*_{i1}+\gamma ^{(Y)}_{2}L^*_{i1}+d^{(Y)}_{i2}) \nonumber \\&\quad = \alpha ^{(Y)}_{2}(\alpha ^{(Y)}_{1}E(Y^*_{i0})+\beta ^{(Y)}_{1}a^*_{i0}+\gamma ^{(Y)}_{1}E(L^*_{i0}) )+\beta ^{(Y)}_{2}a^*_{i1}+\gamma ^{(Y)}_{2}(\alpha ^{(L)}_{1}E(Y^*_{i0})+\beta ^{(L)}_{1}a^*_{i0}+\gamma ^{(L)}_{1}E(L^*_{i0}) )\nonumber \\&\quad = \underbrace{[\alpha ^{(Y)}_{2}(\alpha ^{(Y)}_{1}E(Y^*_{i0})+\gamma ^{(Y)}_{1}E(L^*_{i0}))+ \gamma ^{(Y)}_{2}(\alpha ^{(L)}_{1}E(Y^*_{i0})+\gamma ^{(L)}_{1}E(L^*_{i0}))]}_{0}\nonumber \\&\qquad +\underbrace{[\alpha ^{(Y)}_{2}\beta ^{(Y)}_{1}+\gamma ^{(Y)}_{2}\beta ^{(L)}_{1}]}_{\beta _{20}}a^*_{i0}+\underbrace{\beta ^{(Y)}_{2}}_{\beta _{21}}a^*_{i1}\nonumber \\&\quad = \beta _{20}a^*_{i0}+\beta _{21}a^*_{i1}. \end{aligned}$$

From this result, joint (causal) effects of treatments $A^*_{i0}$ and $A^*_{i1}$ when increasing one unit from the reference values $a^{*r}_{i0}$ and $a^{*r}_{i1}$ become $(\beta _{20}(a^{*r}_{i0}+1)+\beta _{21}(a^{*r}_{i1}+1)) -(\beta _{20}a^{*r}_{i0}+\beta _{21}a^{*r}_{i1})= \beta _{20}+\beta _{21}=\alpha ^{(Y)}_{2}\beta ^{(Y)}_{1}+\gamma ^{(Y)}_{2}\beta ^{(L)}_{1}+\beta ^{(Y)}_{2}.$ Note that $\beta _{20}$ (the effect of intervention $A^*_0=a^*_0$ on $Y^*_2$) can also be evaluated by tracing the two paths $a^*_0 \rightarrow Y^*_1 \rightarrow Y^*_2$ ($=\alpha ^{(Y)}_{2}\beta ^{(Y)}_{1}$) and $a^*_0 \rightarrow L^*_1 \rightarrow Y^*_2$ ($=\gamma ^{(Y)}_{2}\beta ^{(L)}_{1}$) that start at $A^*_0 (=a^*_0)$ and end at $Y^*_2$ in Fig. 1b.^3^ Likewise, $E(Y^*_{i1}| L^*_{i0}, A^*_{i0}=a^*_{i0})$ at $k=1$ can be expressed as

14 $$\begin{aligned} E(\alpha ^{(Y)}_{1}Y^*_{i0}+\beta ^{(Y)}_{1}a^*_{i0}+\gamma ^{(Y)}_{1}L^*_{i0})= \underbrace{[\alpha ^{(Y)}_{1}E(Y^*_{i0})+\gamma ^{(Y)}_{1}E(L^*_{i0})]}_{0}+\underbrace{\beta ^{(Y)}_{1}}_{\beta _{10}}a^*_{i0} =\beta _{10}a^*_{i0}, \end{aligned}$$

thus the causal effect of treatment $A^*_{i0}$ when increasing one unit from the reference values $a^{*r}_{i0}$ at the within-person level becomes $\beta _{10}=\beta ^{(Y)}_{1}$, which is equivalent to the so-called cross-lagged parameter in Eq. (3).

From Eqs. (5) and (6) (i.e., stable trait factors are uncorrelated with within-person variability scores), we have the relation $E(Y^*_{ik}| \bar{L}^*_{i(k-1)}, {\bar{A}^*_{i(k-1)}}\!=\!\bar{a}^*_{i(k-1)})\!=\!E(Y_{ik}|I_{i}^{(Y)}, I_{i}^{(L)}, I_{i}^{(A)}, \bar{L}^*_{i(k-1)}, {\bar{A}^*_{i(k-1)}}=\bar{a}^*_{i(k-1)})-(\mu _{k}^{(Y)}+I_{i}^{(Y)})$. Because $\mu _{k}^{(Y)}+I_{i}^{(Y)}$ is the term that is not associated with treatments at the within-person level, it can be shown that

15 $$\begin{aligned}&E(Y^*_{ik}| \bar{L}^*_{i(k-1)}, {\bar{A}^*_{i(k-1)}}=\bar{a}^*_{i(k-1)}+1)-E(Y^*_{ik}| \bar{L}^*_{i(k-1)}, {\bar{A}^*_{i(k-1)}}=\bar{a}^*_{i(k-1)})\nonumber \\&\quad =E(Y_{ik}|I_{i}^{(Y)}, I_{i}^{(L)}, I_{i}^{(A)}, \bar{L}^*_{i(k-1)}, {\bar{A}^*_{i(k-1)}}\nonumber = \bar{a}^*_{i(k-1)}+1)\\&\qquad -E(Y_{ik}|I_{i}^{(Y)}, I_{i}^{(L)}, I_{i}^{(A)}, \bar{L}^*_{i(k-1)}, {\bar{A}^*_{i(k-1)}}=\bar{a}^*_{i(k-1)}). \end{aligned}$$

The right side of the equation can be interpreted as person-specific joint (causal) effects in the sense that it accounts for stable traits of persons ($I_i^{(Y)}$). Therefore, joint (causal) effects of $A^*$ on $Y^*$ (i.e., Eq. 12; at the within-person level) can be interpreted as person-specific joint (causal) effects on *Y* under the assumed linear causal DAG such as in Fig. 1b.

### Identification Conditions for Causal Parameters

So far, we have assumed a causal DAG model that is similar to that shown in Fig. 1b. In the proposed method, the assumptions for identifying parameters for joint (causal) effects can now be summarized as (i) measurements ($K \ge 2$) are expressed by the linear sum of stable trait factors and within-person variability scores that are mutually uncorrelated, (ii) within-person variability scores are expressed by functions (with assumption of homogeneity) of those in past time, (iii) consistency, (iv) sequential ignorability, (v) SUTVA, (vi) positivity, (vii) modularity, and (viii) multivariate normality (if MLE is used in the first step). The proposed method can be used effectively in applications in which these assumptions are met.

Regarding the second assumption, if $K \ge 2$ and the DGP can be represented by linear (and first-order) equations such as in Eqs. (2) and (3) (assuming homogeneity and no interaction effects of treatments/predictors), then in this special case, the RI-CLPM (that includes time-varying observed confounders) as a statistical model can identify parameters for joint (causal) effects. However, the linearity assumption that is typically imposed for time-varying observed confounders and outcomes (and treatments/predictors) in path modeling and SEM (including the RI-CLPM) has often been criticized in the causal inference literature (e.g., Hong, 2015), and relaxing this assumption is often key to consistently estimating the causal quantity of interest (e.g., Imai & Kim, 2019). In addition, ensuring a correct specification in terms of the linearity is very challenging in that many equations must be diagnosed in longitudinal designs.

As we will see, the proposed method still requires specifications of the structure for within-person variability scores over time in each variable (*Y*, *A*, and *L* in the first step) as well as parametric models for treatments and outcomes at the within-person level (in the second step). However, the assumption of linearity is not required for these parametric models in MSMs and SNMs, and in MSMs one does not need to model the relation between outcomes and time-varying observed confounders (at the within-person level) because it is the means of potential outcomes that are marginalized over these confounders that are of concern. Notably, SNMs with G-estimation have the property of being doubly robust to model misspecifications in how time-varying observed confounders are functionally related to treatments and outcomes (at the within-person level).

## Proposed Methodology

We are now ready to introduce a method of within-person variability score-based causal inference for estimating joint effects of time-varying continuous treatments, assuming that the above conditions for identification are satisfied. The proposed method consists of a two-step analysis. First, within-person variability scores are calculated using weights through SEM that models only the measurement parts that include stable trait factors. Then, causal parameters are estimated by MSMs or SNMs, using the scores calculated in the first step. This approach is more flexible than the one that relies entirely on SEM (e.g., the RI-CLPM that includes time-varying observed confounders) in terms of modeling how time-varying observed confounders are functionally related to treatments/predictors and outcomes at the within-person level, without imposing the linearity assumption in these relations. Before explaining the proposed methodology, we briefly discuss the motivation for adopting a two-step method, rather than simultaneously estimating stable trait factors (or within-person variations) and causal parameters.

In general, partial misspecification in measurements and/or structural models is known to cause large biases in estimates of model parameters. In the present context, when a simultaneous estimation procedure such as the RI-CLPM is used, misspecification in the structural models at the within-person level may greatly affect parameter estimates in the measurement model ((co)variances of stable factors and within-person variability scores), and vice versa.

To avoid such confounding in interpreting the estimation results, in the SEM context Anderson and Gerbing (1988) proposed a two-step procedure that first confirms the measurement model with a saturated model, so that structural relations have no impact on the measurement model. Then, using an appropriate measurement model, the substantive structural relations model of interest is added (Hoshino & Bentler, 2013). Applications of similar multistep estimation procedures can be seen for diverse classes of latent variable models (Bakk & Kuha, 2017; Croon, 2002; Skrondal & Laake, 2001; Vermunt, 2010).

Another potential advantage of two-step estimation is its feasibility. MSMs and SNMs usually do not assume common factors, and the optimization procedure for these models is different from that in SEM. For this reason, fully customized programming is required if performing simultaneous estimation. However, in two-step estimation, parameters in measurement models can be estimated in the first step through various software packages for SEM, including Amos, SAS PROC CALIS, R packages (sem, lavaan, OpenMx), LISREL, EQS, and Mplus. MSMs and SNMs can be straightforwardly applied just by using calculated within-person variability scores instead of measurements.

Two-step estimation is also advantageous because it poses less risk of improper solutions. This problem is encountered relatively often when applying the RI-CLPM because of negative variance parameters and a singular approximate Hessian matrix for stable trait factor variance–covariance (e.g., Usami et al., 2019a), which is likely caused by misspecifications in linear regressions (i.e., the structural model). We will separately estimate stable trait factors for each variable (*Y*, *A*, and *L*) without influence from specified structural models, thus minimizing the risk of improper solutions.

### Step 1: Estimation of Stable Trait Factors and Prediction of Within-Person Variability Scores

The first step of our method is divided into two sub-steps: (i) specification of the measurement models and parameter estimation and (ii) prediction of within-person variability scores.

#### Specification of the Measurement Models and Parameter Estimation

As stated earlier, we assume that measurements are expressed by the linear sum of stable trait factors and within-person variability scores that are mutually uncorrelated, as in Eq. (2). This equation can be viewed as a factor analysis model that includes a single common factor *I* (whose factor loadings are all one)^4^ and a unique factor as temporal deviations. In vector notation, the causal model of Eq. (2) for outcome *Y* becomes

16 $$\begin{aligned} Y_{i}=\mu ^{(Y)}+I^{(Y)}_{i}1_{K+1}+Y^*_i, \end{aligned}$$

where $\mu ^{(Y)}$ is a $(K+1) \times 1$ mean vector, $E(I^{(Y)}_{i})=0$, $\text {Var}(I^{(Y)}_{i})=\phi ^2_{(Y)}$, $E(Y^*_i)=0$, and $\text {Cov}(I^{(Y)}_i,Y^*_i)=0$. We denote as $\Psi _{(Y)}$ a $(K+1) \times (K+1)$ variance–covariance matrix of within-person variability scores. This implies that the variance–covariance matrix of *Y* (denoted as $\Sigma _{(Y)}$) is of the form $\Sigma _{(Y)}=\phi ^2_{(Y)}1_{K+1}1^t_{K+1}+\Psi _{(Y)}$.

Unlike the standard factor analysis model, $\Psi _{(Y)}$ has a dependence structure and is not diagonal. Therefore, in using SEM to estimate the parameters in Eq. (16), some structure—such as compound symmetry, a Toeplitz structure, or a (first-order) autoregressive (AR) structure—must be specified in $\Psi _{(Y)}$ for model identification. When the model is correctly specified, consistent estimators for $\mu ^{(Y)}$, $\phi ^2_{(Y)}$, and $\Psi _{(Y)}$ can be obtained by MLE in SEM (Jöreskog & Lawley, 1968).

In SEM, missing values can be easily handled by full information maximum likelihood (Enders & Bandalos, 2001) with the assumption of missing at random (MAR; Rubin, 1976). If data are suspected to be missing not at random (MNAR), then appropriate sensitivity analyses and/or multiple imputation should be considered (Resseguier et al., 2011). Models that account for MNAR can be easily estimated in popular software packages for SEM (see Enders, 2011; Newsom, 2015).

Another advantage of SEM is that validity of the specified model can be diagnosed via multiple model fit indices, along with model comparisons using information criteria. In this paper, we use three current major indices (e.g., Hu & Bentler, 1999; Kline, 2016): (a) the comparative fit index (CFI), (b) the root-mean-square error of approximation (RMSEA), and (c) the standardized root-mean-square residual (SRMR).

Similarly, we also set measurement models for treatments/predictors *A* and observed confounders *L* separately in this sub-step, then estimate parameters for mean vectors ($\mu ^{(A)}$ and $\mu ^{(L)}$), stable trait factor variances ($\phi ^2_{(A)}$ and $\phi ^2_{(L)}$), and variance–covariance matrices of within-person variability scores $\Psi _{(A)}$ and $\Psi _{(L)}$.

#### Predicting Within-Person Variability Scores

Let $X_i=(Y_i,A_i,L_i)^t$ and $X^*_i=(Y^*_i,A^*_i,L^*_i)^t$ be vectors of measurements and within-person variability scores, respectively, and let $\mu =(\mu ^{(Y)},\mu ^{(A)},\mu ^{(L)})^t$ be a mean vector. Also let $\Sigma$ and $\Psi$ be covariance matrices for measurements $X_i$ and within-person variability scores $X^*_i$.

We consider linear prediction of within-person variability scores ${\hat{X}}^*_i$ under the condition that $\Sigma$ and $\Psi$ are known. Consider a $(3K+1) \times (3K+1)$ weight matrix *W* that provides within-person variability scores from measurements as

17 $$\begin{aligned} {\hat{X}}^*_i=W^t(X_i-\mu ), \end{aligned}$$

satisfying the relation

18 $$\begin{aligned} E({\hat{X}}^*_i{\hat{X}}^{*t}_i)=W^tE[(X_i-\mu )(X_i-\mu )^t]W=W^t\Sigma W=\Psi . \end{aligned}$$

Unlike standard applications of factor analysis, we are interested in predicting within-person variability (unique factor) scores, rather than stable trait factor (common factor) scores. However, the current problem of determining weights *W* shares the similar motivation of predicting factor scores. In the factor analysis literature, a predictor that preserves the covariance structure of common factors has been developed as a linear correlation preserving predictor (Anderson & Rubin, 1956; Green, 1969; ten Berge et al., 1999).

With this point in mind, *W* that can provide the best linear predictor of ${\hat{X}^*}_i$ minimizing the risk function, defined as the trace of a residual covariance matrix (i.e., mean squared error MSE(${\hat{X}}^*_i$)=$E[({\hat{X}^*}_i-X^*_i)^t({\hat{X}^*}_i-X^*_i)]$), which also satisfies the relation in Eq. (18), can be obtained by utilizing singular value decomposition as

19 $$\begin{aligned} W^t=\Psi ^{1/2}{(\Psi ^{3/2}\Sigma ^{-1}\Psi ^{3/2})}^{-1/2}\Psi ^{3/2}\Sigma ^{-1}. \end{aligned}$$

Here, for a positive (semi)definite matrix *C*, we denote as $C^{1/2}$ the positive (semi)definite matrix such that its square equals *C*. Matrices $C^{-1/2}$ and $C^{3/2}$ are the inverse (if it exists) and the third power of $C^{1/2}$, respectively. A derivation of *W* is provided in the Online Supplemental Material.

We use the sample means $\bar{X}$ and covariance matrix *S* of *X* as estimators of $\mu$ and $\Sigma$. As implied from the relation in Eq. (7), we use estimated stable trait factor variances to estimate $\Psi$ as

20 $$\begin{aligned} {\hat{\Psi }} = S-{\hat{\Phi }}^+, \end{aligned}$$

where ${\hat{\Phi }}^+$ consists of estimated stable trait factor (co)variances. In the simple case where the initial measurement of *Y* ($Y_0$) is missing and the number of measurements equals *K* for each variable, ${\hat{\Phi }}^+$ becomes

21 $$\begin{aligned} {\hat{\Phi }}^+={\hat{\Phi }} \otimes 1_K1^t_K=\left( \begin{array}{lll} {\hat{\phi }}^2_{(Y)} &{}\quad {\hat{\phi }}_{(Y,A)} &{}\quad {\hat{\phi }}_{(Y,L)} \\ {\hat{\phi }}_{(Y,A)} &{}\quad {\hat{\phi }}^2_{(A)} &{}\quad {\hat{\phi }}_{(A,L)} \\ {\hat{\phi }}_{(Y,L)} &{}\quad {\hat{\phi }}_{(A,L)} &{}\quad {\hat{\phi }}^2_{(L)} \end{array} \right) \otimes 1_K1^t_K, \end{aligned}$$

where ${\hat{\Phi }}$ is an estimator of a $3 \times 3$ stable trait factor covariance matrix $\Phi$. Because stable trait factor covariances are not estimated in the previous sub-step, we use covariances between calculated linear correlation preserving predictors for variables. For example, this predictor for *Y* can be expressed as

22 $$\begin{aligned} \hat{I}_{i}^{(Y)}=\frac{{\hat{\phi }}_{(Y)}}{\sqrt{1_{K+1}^t{{\hat{\Sigma }}^{-1}_{(Y)}}1_{K+1}}}1_{K+1}^t{{\hat{\Sigma }}^{-1}_{(Y)}}(Y_i-\bar{Y}). \end{aligned}$$

$\hat{I}_{i}^{(A)}$ and $\hat{I}_{i}^{(L)}$ can be calculated in the same manner, whereby we obtain ${\hat{\phi }}_{(Y,A)}=\text {Cov}(\hat{I}_{i}^{(Y)},\hat{I}_{i}^{(A)})$, ${\hat{\phi }}_{(Y,L)}=\text {Cov}(\hat{I}_{i}^{(Y)},\hat{I}_{i}^{(L)})$, and ${\hat{\phi }}_{(A,L)}=\text {Cov}(\hat{I}_{i}^{(A)},\hat{I}_{i}^{(L)})$. Predictors $\hat{I}_i=(\hat{I}_{i}^{(Y)},\hat{I}_{i}^{(A)},\hat{I}_{i}^{(L)})^t$ satisfy the relation $E(\hat{I}_{i}\hat{I}_{i}^t)=\Phi$ if the model is correctly specified in the previous sub-step. From Eqs. (17) and (19)–(21), we can thus obtain ${\hat{X}}^*_i$ without specifying the structural models that connect within-person variability scores from different variables ($Y^*$, $A^*$, and $L^*$), successfully maintaining independence from the next step.

### Applying MSMs and SNMMs

The second step of the proposed method is straightforward, because we just need to apply MSMs or SNMs using calculated within-person variability scores. Robins and co-workers developed SNMs with G-estimation (Robins, 1989; Robins et al., 1992) and MSMs with an inverse probability weight (IPW) estimator (Robins, 1999; Robins et al., 2000). These methods have been extended to treat clustered outcomes (e.g., Brumback et al., 2014; He et al., 2015, 2019). However, (joint) causal effects under the control of stable trait factors have not been investigated in this area because inference for stable traits and within-person relations has been an issue in the psychometric and behavioral science literature, and these concepts have yet to be fully characterized in the causal inference literature.

MSMs are advantageous in that they can be easily understood and fit with standard, off-the-shelf software that allows for weights (e.g., He et al., 2019; Vansteelandt & Joffe, 2014). However, it is well known that MSMs can be highly sensitive to misspecification of the treatment assignment model, even when there is a moderate number of time points (e.g., Hong, 2015; Lefebvre et al., 2008). Imai and Ratkovic (2015) proposed a covariate balancing propensity score methodology for robust IPW estimation.

Because of the attractive property of being doubly robust in G-estimators, SNMs are a better approach for handling violation of the usual assumptions of no unmeasured confounders or sequential ignorability (Vansteelandt & Joffe, 2014). In addition, SNMs can allow direct modeling of the interactions and moderation effects of treatments/predictors *A* with observed confounders *L*. Another advantage of SNMs is that the variance of locally efficient IPW estimators in MSMs exceeds that of G-estimators in SNMs, unless *A* and *L* are independent. We therefore emphasize the utility of SNMs in this paper. Because we are now interested in evaluating the joint effects of treatments on the mean of an outcome, rather than those on the entire distribution of the outcome, we apply structural nested mean models (SNMMs; Robins, 1994).

Note that potential disadvantages of SNMs are their limited utility for G-estimation when applying logistic SNMs and their limited availability of off-the-shelf software. Regarding the latter point, Wallace, Moodie, and Stephens (2017) developed an R package for G-estimation of SNMMs.

#### MSMs Using Within-Person Variability Scores

MSMs are typically applied to evaluate the joint effects of a sequence of treatments on the outcome, which is measured only at the end of a fixed follow-up period ($t_K$). For generality of discussion, as before we assume that the outcome is measured each time and that the primary interest is evaluation of effects of a sequence of past treatments on the outcome at each time point.

MSMs consider the marginal mean of potential outcomes that are marginalized over the observed confounders *L*. In the current context, we consider potential outcomes at the within-person level, namely, $E({Y^*_{ik}}^{\bar{A}^*_{i(k-1)}})$ with treatment history $\bar{A}^*_{i(k-1)}=\bar{a}^*_{i(k-1)}$. $E({Y^*_{ik}}^{\bar{A}^*_{i(k-1)}})$ might take the form

23 $$\begin{aligned} E({Y^*_{ik}}^{\bar{A}^*_{i(k-1)}})=\sum _{t=1}^{k}\beta _{k(t-1)}A^*_{i(t-1)} \end{aligned}$$

with $k=1,2,\dots ,K.$^5^ The average joint (causal) effects of $\bar{A}^*_{i(k-1)}$ on $Y^*_{ik}$ when increasing one unit from the reference values in each treatment become $\sum _{t=1}^k\beta _{k(t-1)}$. Parameters $\tau =(\beta _{k0}, \beta _{k1}, \dots , \beta _{k(k-1)})^t$ can be estimated by fitting a weighted conditional model with an IPW estimator. One useful option for calculating weights is to use stabilized weights $w_{ik}$ for person *i* at time point $t_k$ (Hernán et al., 2002) as

24 $$\begin{aligned} w_{ik}=\prod _{t=1}^{k-1}\frac{f(A^*_{it}|A^*_{i(t-1)})}{f(A^*_{it}|A^*_{i(t-1)},L^*_{it})}, \end{aligned}$$

where $f(A^*_{it}|A^*_{i(t-1)},L^*_{it})>0$ for all $A^*_{it}$, if $f(A^*_{i(t-1)},L^*_{it}) \ne 0$ (the positivity assumption). Parameters will be biased if the treatment assignment model $f(A^*_{it}|A^*_{i(t-1)},L^*_{it})$ is misspecified, but misspecification of $f(A^*_{it}|A^*_{i(t-1)})$ does not result in bias. In MSMs, unlike the RI-CLPM (that includes *L*), one does not need to model the relation between outcomes and time-varying observed confounders at the within-person level because marginal (joint) effects of treatments/predictors are the primary focus in applying this method. Also, one can allow a nonlinear relation between treatments/predictors and confounders in the treatment assignment model $f(A^*_{it}|A^*_{i(t-1)},L^*_{it})$, although estimates are sensitive to this model misspecification.

#### SNMMs Using Within-Person Variability Scores

SNMMs simulate the sequential removal of an amount (*blip*) of treatment at $t_{k-1}$ on subsequent average outcomes, after having removed the effects of all subsequent treatments. SNMMs then model the effect of a blip in treatment at $t_{k-1}$ on the subsequent outcome means while holding all future treatments fixed at a reference level 0 (Vansteelandt & Joffe, 2014); in other words, the level that is equal to expected scores of a person in the current context.

SNMMs parameterize contrasts of ${\underline{Y}^*}_{ik}^{\bar{a}^*_{i(k-1)},0}$ and ${\underline{Y}^*}_{ik}^{\bar{a}_{i(k-2)},0}$ conditionally on treatments/predictors and confounder histories through $t_{(k-1)}$ as

25 $$\begin{aligned} g[E({\underline{Y}^*}_{ik}^{\bar{a}^*_{i(k-1)},0}|\bar{L}^*_{i(k-1)}&=\bar{l}^*_{i(k-1)}, \bar{A}^*_{i(k-1)}=\bar{a}^*_{i(k-1)})]- g[E({\underline{Y}^*}_{ik}^{\bar{a}^*_{i(k-2)},0}|\bar{L}^*_{i(k-1)}=\bar{l}^*_{i(k-1)}, \bar{A}^*_{i(k-1)}=\bar{a}^*_{i(k-1)})]\nonumber \\ {}&=h_{k}(\bar{l}^*_{i(k-1)},\bar{a}^*_{i(k-1)};\tau ) \end{aligned}$$

for each $k=1,\dots ,K$, where $g(\cdot )$ is a known link function, and $h_{k}(\bar{l}^*_{i(k-1)},\bar{a}^*_{i(k-1)};\tau )$ is a known $(K-k+1)$-dimensional function, smooth in the finite-dimensional parameter $\tau$ (Vansteelandt & Joffe, 2014).

In the following empirical applications using the data of $K=2$, a linear SNMM using the identity link $g(x) = x$ is given by

26 $$\begin{aligned}&E({Y^*_{i2}}^{a_{i0},a_{i1}}-{Y^*_{i2}}^{a_{i0},0} | \bar{L}^*_{i1}=\bar{l}^*_{i1},\bar{A}^*_{i1}=\bar{a}^*_{i1})=(\beta _{21}+\gamma _{21} l^*_{i1})a^*_{i1}, \nonumber \\&E({Y^*_{i2}}^{a_{i0},0}-{Y^*_{i2}}^{0,0} | L^*_{i0}=l^*_{i0},A^*_{i0}=a^*_{i0})=(\beta _{20}+\gamma _{20}l^*_{i0})a^*_{i0}, \nonumber \\&E({Y^*_{i1}}^{a_{i0},0}-{Y^*_{i1}}^{0,0} | L^*_{i0}=l^*_{i0},A^*_{i0}=a^*_{i0})=(\beta _{10}+\gamma _{10}l^*_{i0})a^*_{i0}. \end{aligned}$$

Here, the first equation models the effect of $A^*_{i1}$ on $Y^*_{i2}$, the second models the effect of $A^*_{i0}$ on $Y^*_{i2}$, and the third models the effect of $A^*_{i0}$ on $Y^*_{i1}$. The (conditional) average joint effects of $A^*_{i0}$ and $A^*_{i1}$ on $Y^*_{i2}$ when increasing one unit from the reference values in each treatment become $\beta _{20}+\gamma _{20}l^*_{i0}+\beta _{21}+\gamma _{21}l^*_{i1}$. This effect becomes $\beta _{20}+\beta _{21}$ if there are no interaction effects between confounders and treatments.

SNMMs consider a transformation $U^*_{im}(\tau )$ of $\underline{Y}^*_{ik}$, the mean value of which is equal to the mean that would be observed if treatment were stopped from time $t_{k-1}$ onward, in the sense that

27 $$\begin{aligned}&E(U^*_{i(k-1)}(\tau )|\bar{L}^*_{i(k-1)},\bar{A}^*_{i(k-2)}= \bar{a}^*_{i(k-2)},A^*_{i(k-1)}) \nonumber \\&=E({\underline{Y}^*}_{ik}^{\bar{a}^*_{i(k-2)},0}|\bar{L}^*_{i(k-1)},\bar{A}^*_{i(k-2)}= \bar{a}^*_{i(k-2)},A^*_{i(k-1)}) \end{aligned}$$

for $k=1,\dots ,K$. Here, $U^*_{i(k-1)}(\tau )$ is a vector with components $Y^*_{im}-\sum _{l=k-1}^{m-1}h_{l,m}(\bar{L}^*_{il},\bar{A}^*_{il};\tau )$ for $m=k,\dots ,K$ if $g(\cdot )$ is the identity link. For instance, in the above example of $K=2$,

28 $$\begin{aligned}&U_{i1}^*(\tau )=Y^*_{i2}-(\beta _{21}+\gamma _{21}L^*_{i1})A^*_{i1}, \nonumber \\&U_{i0}^*(\tau )=(Y^*_{i1}-(\beta _{10}+\gamma _{10}L^*_{i0})A^*_{i0}, Y^*_{i2}-(\beta _{21}+\gamma _{21}L^*_{i1})A^*_{i1}-(\beta _{20}+\gamma _{20}L^*_{i0})A^*_{i0})^t. \end{aligned}$$

The assumptions of sequential ignorability (Eq. 10) together with identity (Eq. 27) imply that

29 $$\begin{aligned} E(U^*_{i(k-1)}(\tau ^*) |\bar{L}^*_{i(k-1)},\bar{A}^*_{i(k-1)}) = E(U^*_{i(k-1)}(\tau ^*) |\bar{L}^*_{i(k-1)},\bar{A}^*_{i(k-2)}) \end{aligned}$$

for $k=1,\dots ,K$. The parameters $\tau$ can therefore be estimated by solving the estimating equation

30 $$\begin{aligned}&\sum _{i=1}^N\sum _{k=1}^{K}[d_{k-1}(\bar{L}^*_{i(k-1)},\bar{A}^*_{i(k-1)}) -E(d_{k-1}(\bar{L}^*_{i{k-1}},\bar{A}^*_{i{k-1}})|\bar{L}^*_{i(k-1)},\bar{A}^*_{i(k-2)})] \circ \nonumber \\&\quad V^{-1} \circ [U^*_{i(k-1)}(\tau )-E(U^*_{i(k-1)}(\tau )|\bar{L}^*_{i(k-1)},\bar{A}^*_{i(k-2)})]=0, \end{aligned}$$

where $d_{k-1}(\bar{L}^*_{i(k-1)},\bar{A}^*_{i(k-1)})$ is an arbitrary $p \times (K-k+1)$-dimensional function, with *p* the dimension of $\tau$, and $V^{-1}$ is a $p \times (K-k+1)$-dimensional vector that includes the reciprocal of the variance of each element in $U^*_{i(k-1)}(\tau )-E(U^*_{i(k-1)}(\tau )|\bar{L}^*_{i(k-1)},\bar{A}^*_{i(k-2)})$.

This estimating equation essentially sets the sum across the time points of the conditional covariances between $U^*_{i(k-1)}(\tau )$ and the function $d_{k-1}(\bar{L}^*_{i(k-1)},\bar{A}^*_{i(k-1)})$, given $\bar{L}^*_{i(k-1)}$ and $\bar{A}^*_{i(k-2)}$, are zero. If there is homoscedasticity in *V*, then local semiparametric efficiency under the SNMM is attained upon choosing

31 $$\begin{aligned} d_{k-1}(\bar{L}^*_{i(k-1)},\bar{A}^*_{i(k-1)})=E\left[ \left. \frac{\partial U^*_{i(k-1)}(\tau ^*)}{\partial \tau }\right| \bar{L}^*_{i(k-1)},\bar{A}^*_{i(k-1)}\right] \end{aligned}$$

(Vansteelandt & Joffe, 2014). Solving estimating equation (30) requires a parametric model $\mathcal {A}$ for the treatment/predictor $A^*_{ik}$: $f(A^*_{i(k-1)} | \bar{L}^*_{i(k-1)},\bar{A}^*_{i(k-2)};\eta )$ with $k=1,\dots ,K$. It also requires a parametric model $\mathcal {B}$ for the conditional mean of $U^*_{i(k-1)}(\tau )$, namely, $f(U^*_{i(k-1)}(\tau ) | \bar{L}^*_{i(k-1)},\bar{A}^*_{i(k-2)};\kappa )$. Notably, when the parameters $\eta$ and $\kappa$ are variation-independent, G-estimators that solve Eq. (30), obtained by substituting $\eta$ and $\kappa$ with consistent estimators, are doubly robust (Robins & Rotnitzky, 2001, cited from Vansteelandt & Joffe, 2014), meaning that estimates of causal parameters are consistent when either model $\mathcal {A}$ or model $\mathcal {B}$ is correctly specified. In addition, unlike the RI-CLPM (that includes *L*), one can allow nonlinear effects of time-varying observed confounders on treatments/predictors and outcomes in models $\mathcal {A}$ and $\mathcal {B}$.

## Simulation Studies

### Method

This section describes a Monte Carlo simulation for systematically investigating how effectively the proposed method using calculated within-person variability scores can recover causal parameters, and it presents comparisons of estimation performance versus other potential (centering) methods to account for stable traits. We consider two different scenarios: (i) the assumed linear (and first-order) DGP of Fig. 1b (i.e., causal models represented in Eqs. 2 and 3) is correct and other assumptions of consistency, sequential ignorability, SUTVA, positivity, modularity, and multivariate normality are all satisfied, and (ii) some assumptions are violated and the statistical model contains misspecifications. In the whole simulation, for simplicity we also assume that causal effects are homogeneous among persons and interactions or moderation effects with observed confounders are not present.

In the first scenario, initial within-person variability scores ($Y^*_{i0}$, $A^*_{i0}$, and $L^*_{i0}$) are first generated so that they are normally distributed and their variances and covariances become 10 and 3, respectively. Then, within-person variability scores at succeeding times are sequentially generated via a first-order linear autoregressive model (i.e., Eq. 3) with the stationarity assumption^6^:

32 $$\begin{aligned} Y^*_{ik}&=0.40Y^*_{i(k-1)}+0.40A^*_{i(k-1)}+0.10L^*_{i(k-1)}+d^{(Y)}_{ik},\nonumber \\ A^*_{ik}&=0.20Y^*_{ik}+0.40A^*_{i(k-1)}+0.30L^*_{ik}+d^{(A)}_{ik}, \nonumber \\ L^*_{ik}&=0.20Y^*_{i(k-1)}+0.20A^*_{i(k-1)}+0.50L^*_{i(k-1)}+d^{(L)}_{ik}, \end{aligned}$$

If $K=4$, this setting produces (see also the calculation in Eq. 13)

33 $$\begin{aligned}&E({Y^*_{i4}}^{\bar{a}^*_{i3}})=\beta _{40}a^*_{i0}+\beta _{41}a^*_{i1}+\beta _{42}a^*_{i2}+\beta _{43}a^*_{i3}=0.0486a^*_{i0}+0.09a^*_{i1}+0.18a^*_{i2}+0.40a^*_{i3},\nonumber \\&E({Y^*_{i3}}^{\bar{a}^*_{i2}})=\beta _{30}a^*_{i0}+\beta _{31}a^*_{i1}+\beta _{32}a^*_{i2}=0.09a^*_{i0}+0.18a^*_{i1}+0.40a^*_{i2},\nonumber \\&E({Y^*_{i2}}^{\bar{a}^*_{i1}})=\beta _{20}a^*_{i0}+\beta _{21}a^*_{i1}=0.18a^*_{i0}+0.40a^*_{i1},\nonumber \\&E({Y^*_{i1}}^{a^*_{i0}})=\beta _{10}a^*_{i0}=0.40a^*_{i0}. \end{aligned}$$

Because no moderation effects are assumed, estimating 10 different causal parameters $\tau _{K=4}=(\beta _{10},\beta _{20},\beta _{21},\beta _{30},\beta _{31},\beta _{32},\beta _{40},\beta _{41},\beta _{42},\beta _{43})^t$ is a common goal between MSMs and SNMMs. The variance of normal residual *d* was set to 5 for each variable, making the variance of within-person variability scores for each variable become almost 10 at each time point (the proportion of variance explained in Eq. (32) becomes almost 50%).

Independently of generating within-person variability scores, three kinds of stable trait factors ($I^{(Y)}_{i}$, $I^{(A)}_{i}$, and $I^{(L)}_{i}$) are generated by multivariate normal with a correlation of 0.3. Observed values are then generated using the relation of Eq. (2),

34 $$\begin{aligned} Y_{ik}=I^{(Y)}_{i}+Y^*_{ik}, \quad A_{ik}=I^{(A)}_{i}+A^*_{ik}, \quad L_{ik}=I^{(L)}_{i}+L^*_{ik}, \end{aligned}$$

where temporal group means are set to zero at each time point (i.e., $\mu ^{(Y)}_{k}=\mu ^{(A)}_{k}=\mu ^{(L)}_{k}=0).$

In this simulation, we systematically changed the total number of persons as $N= 200, 600$, and 1000, the number of time points as $K= 4$ and 8, and the size of stable trait factor variances as $\phi _{(Y)}^2=\phi _{(A)}^2=\phi _{(L)}^2= 10/9, 30/7$, and 10. This setting of stable trait factor variances indicates that the proportion of this variance to that of measurements becomes around 10%, 30%, and 50%, respectively, at each time point. To make it easier to compare the results between the $K=4$ and $K=8$ conditions, in $K=8$ we suppose only $A^*_{i4}$, $A^*_{i5}$, $A^*_{i6}$, and $A^*_{i7}$ are intervened, while controlling for $A^*_{i0}$, $A^*_{i1}$, $A^*_{i2}$, and $A^*_{i3}$. This setting produces conditional means of (potential) outcomes as functions of treatments intervened: $\beta _{84}a^*_{i4}+\beta _{85}a^*_{i5}+\beta _{86}a^*_{i6}+\beta _{87}a^*_{i7} =0.0486a^*_{i4}+0.09a^*_{i5}+0.18a^*_{i6}+0.40a^*_{i7}$ at $k=8$, $\beta _{74}a^*_{i4}+\beta _{75}a^*_{i5}+\beta _{76}a^*_{i6} =0.09a^*_{i4}+0.18a^*_{i5}+0.40a^*_{i6}$ at $k=7$, $\beta _{64}a^*_{i4}+\beta _{65}a^*_{i5} =0.18a^*_{i4}+0.40a^*_{i5}$ at $k=6$, and $\beta _{54}a^*_{i4}=0.40a^*_{i4}$ at $k=5$. There are a total of 10 causal parameters $\tau _{K=8}=(\beta _{54},\beta _{64},\beta _{65},\beta _{74},\beta _{75},\beta _{76},\beta _{84},\beta _{85},\beta _{86},\beta _{87})^t$ that are equal to those in the $K=4$ condition (i.e., $\tau _{K=4}=\tau _{K=8}$).

By crossing these factors, we generated 200 simulation data for each combination of factors. For comparison, each simulation dataset was analyzed by MSMs and SNMMs using four different scores: (1) true within-person variability scores (true factor score centering: e.g., ${Y}^*_{ik}=Y_{ik}-I^{(Y)}_i$ for *Y*), (2) within-person variability scores predicted by the proposed method (Eq. 17), (3) scores based on observed person-specific means (observed-mean centering, e.g., ${\hat{Y}}^*_{ik}=Y_{ik}-\bar{Y}_i$, where $\bar{Y}_i=\sum _{k=0}^KY_{ik}/(K+1)$), and (4) observed scores (no centering, e.g., ${\hat{Y}}^*_{ik}=Y_{ik}$). In the current scenario, the no-centering method totally ignores the presence of stable traits. On the other hand, because observed means include the components of both stable traits (between-person differences) and within-person variability, observed-mean centering fails to perfectly disentangle stable individual differences from within-person variability.^7^ Under each simulation condition, we calculated the bias and root-mean-squared error (RMSE) of 10 kinds of estimates of causal parameters from MSMs and SNMMs.

In the first step of the proposed method, to identify the measurement model (e.g., Eq. 16 for *Y*) SEM that assumes a linear AR(1) structure with time-varying autoregressive parameters and residual variances is specified for within-person variability scores in each variable. Although a true model (i.e., AR(*K*) structure) cannot be specified because of the identification problem, we confirmed that the AR(1) structure generally provides acceptable model fits under the current parameter setting.

The results are discarded when improper solutions appear in the first step because of out-of-range parameter estimates (e.g., negative variance). In the current simulation, fewer than 0.1% of all estimates produced such improper solutions. We also confirmed that improper solutions were not found in the second step of applying MSMs and SNMMs. When applying MSMs, a first-order linear regression model is specified for the treatment assignment model, namely, $f(A_t|A_{t-1},L_{t})$ (i.e., the correct specification). For SNMMs, models $\mathcal {A}$ and $\mathcal {B}$ are also specified in an appropriate manner.

In the second scenario where model misspecifications are present, we assume various DGPs in which (a) measurement errors are present, (b) time-invariant factors do not influence measurements as stable trait factors, and (c) quadratic effects of time-varying observed confounders are present in the treatment assignment model, keeping the other conditions the same from the first scenario. More specifically, in (a), all measurements are influenced by normally distributed measurement errors with variances of 10% or 20% of those of the initial measurements (=10+$\phi ^2$). In (b), the relation between outcomes and time-invariant factors (*I*) is set as $Y_{ik}=(1+0.5k/K)I^{(Y)}_{i}+0.3I^{(A)}_{i}+0.3I^{(L)}_{i}+Y^*_{ik}$, (i.e., time-varying loadings from $I^{(Y)}$ and those from other variables ($I^{(A)}$ and $I^{(L)}$) are present), resulting from the assumed DGP such as that in Fig. 1a in which time-invariant factors have both direct and indirect effects on measurements. In (c), quadratic effects from time-varying observed confounders are included in the treatment assignment model as $A^*_{ik}=0.20Y^*_{ik}+0.08Y^{*2}_{ik}+0.40A^*_{i(k-1)}+0.30L^*_{ik}+d^{(A)}_{ik}$, indicating that the treatment assignment model that includes only linear effects of $Y^*_{ik}$ assumed in the current MSM and SNMM is misspecified. Note that causal parameters for time-varying treatments ($\tau$) remain unchanged even if quadratic effects exist in the treatment assignment model because time-varying treatments are now intervened.

The simulation was conducted in R, using the *lavaan* package (Rosseel, 2012) to estimate parameters by SEM with MLE in the first step and the *ipw* package for MSMs in the second step. In SNMMs, we solve Eq. (30) via the Newton–Raphson method. Simulation code is available in the Online Supplemental Material.

**Fig. 2:**
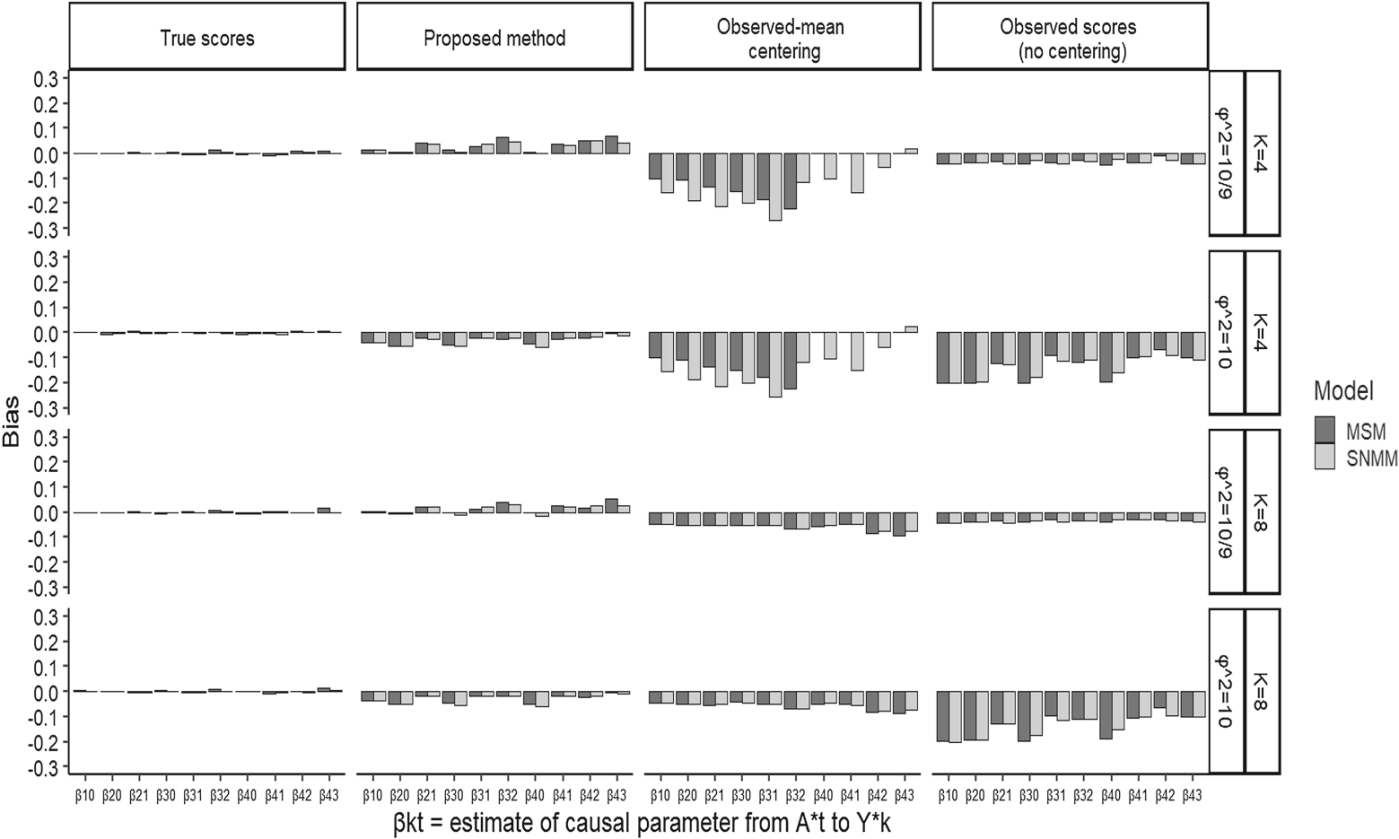
Biases of causal effects estimates ($N = 1000$). Note: Because of rank deficient, in $K=4$ estimates of $\beta _{{40}} ,\beta _{{41}} ,\beta _{{42}} ,\beta _{{43}}$ are not available in marginal structural model with observed mean centering.

### Results

Because of space limitations, Fig. 2 shows only biases of estimates of causal parameters in MSMs and SNMMs when $\phi ^2= 10/9$ and 10. Because differences in the *N* value were minor in terms of bias, here we only show the result when $N=1000$. Results under other conditions are provided in the Online Supplemental Material (Figs. S3 and S4).

Figure 2 shows that true score conditions produce almost no biases in both MSMs and SNMMs. SNMMs show smaller RMSEs compared with MSMs on average (Fig. S2). In the proposed method, estimates show biases because of the biased estimates of stable trait factor (co)variances triggered by a model misspecification in the first step. However, the magnitude of biases is much smaller than in the observed-mean centering and no-centering methods. SNMMs again show smaller RMSEs than do MSMs (Fig. S2). The observed-mean centering method shows negative biases, and their magnitude becomes larger when $K=4$. This result is caused by negatively biased covariances in variables resulting from subtracting observed means from measurements, and this impact increases as *K* decreases. Another critical aspect of this method is that linear dependence prevents identification of joint effects of all past treatments on $Y_K$ (in this case, $\beta _{40}$, $\beta _{41}$, $\beta _{42}$, $\beta _{43}$ in $K=4$). We therefore do not recommend use of observed-mean centering. The no-centering method shows serious negative biases when $\phi ^2$ is not small, indicating that ignoring the presence of stable traits is critical to estimating causal effects. Magnitudes of stable trait factor variances should vary depending on the nature of variables and study period, but in the author’s experience many studies that applied the RI-CLPM have shown significant and moderate to large sizes of ${\hat{\phi }}^2$ (e.g., the proportion of stable trait factor variance to that of measurements is above 30%). The following application also demonstrates large stable trait factor variance estimates.

As supplemental analyses, we additionally explored the performance of the methods under different parameter settings, as well as different model specification of SEM in the first step. From this, we find similar tendencies in the results (Figs. S5–S8): (a) SNMMs show smaller RMSEs than do MSMs, and (b) the proposed method shows adequate performance in terms of biases and RMSEs, and it works better than the no-centering method (especially when $\phi$ is larger) and the observed-mean centering method (especially when *K* is smaller). We also investigated the performance of linear correlation preserving predictor (${\hat{I}}^{(Y)}_i$ in Eq. 22) centering (e.g., ${\hat{Y}}^*_{ik}=Y_{ik}-{\hat{I}}^{(Y)}_i$), confirming that the proposed method worked much better than this method on average (Figs. S5–S8).

Similar results were also observed in the second scenario, where model misspecifications are present (Figs. S9–S14). More specifically, when measurement errors were present, the biases and RMSEs became larger in all methods (Figs. S9 and S10). However, the proposed method still outperforms other centering methods. When time-invariant factors do not influence measurements as stable trait factors, the overall results of biases and RMSEs were not largely affected (Figs. S11–S12), regardless of the magnitude of $\phi ^2$. This result is a little surprising, considering that the specified time-varying loadings from factors ($=1+0.5k/K$ at time $t_k$) are not small (i.e., the impact of this factor on the variance of measurement at time $t_K$ is almost twice that at time $t_0$). This may suggest that causal parameters can be recovered relatively well even when ignoring time-varying impacts from time-invariant factors that are actually present in the first step. However, future investigations are required in order to better clarify when estimated causal parameters are seriously biased under various scenarios for misspecified measurement models. When quadratic effects from time-varying observed confounders are present in the treatment assignment model but are ignored in analyses, biases and RMSEs in MSMs become larger on average. In the proposed method, this is salient in the RMSEs for the $K=8$ and $\phi ^2=10$ conditions (Figs. S13–S14). SNMMs, which have the property of being doubly robust in G-estimators, were less influenced even if these by-no-means small quadratic effects are ignored, and in many conditions the proposed method again outperforms other centering methods.

## Empirical Application

This section describes an empirical application of the proposed method using data from the Tokyo Teen Cohort (TTC) study (Ando et al., 2019). We assume a similar causal DAG model to that in Fig. 1b: (i) measurements are expressed by the linear sum of stable trait factors and within-person variability scores, (ii) within-person variability scores are expressed by functions (with assumption of homogeneity) of those in past time, along with (iii) consistency, (iv) sequential ignorability, (v) SUTVA, (vi) positivity, and (vii) modularity.

TTC was a multidisciplinary longitudinal cohort study on the psychological and physical development of adolescents who were 10 years old at enrollment and lived in municipalities in the Tokyo metropolitan area (Setagaya, Mitaka, Chofu). Datasets were collected in three waves: from 2012 to 2015, from 2014 to 2017, and from 2017 to 2019 (i.e., $K=2$). In total, 3171 children participated in the survey. See Ando et al. (2019) for more detailed information about measured variables, participant recruitment, and demographic characteristics of participants in the TTC study.

In this example, we estimate the (joint) causal effects of time-varying sleep duration (*A*) on later depressive symptoms (*Y*) in adolescents. Several epidemiological studies have suggested a relationship between sleep habits (sleep duration, bedtime, and bedtime regularity) and mental health status (depression and anxiety) in adolescents. For example, Matamura et al. (2014) applied the CLPM to data from 314 monozygotic twins living in Japan and showed that sleep duration had significant associations with mental health indices, even after controlling for genetic and shared environmental factors. However, to the author’s knowledge, no studies have investigated this relation that accounts for stable traits in sleep duration and symptoms (i.e., at the within-person level).

The Short Mood and Feelings Questionnaire (SMFQ; Angold et al., 1995) was used to measure depression in adolescents (*Y*). The SMFQ consists of 13 items assessing depressive symptoms rated on a three-point scale (0: *not true*, 1: *sometimes true*, 2: *true*) regarding feelings and actions over the preceding two weeks. Higher SMFQ scores suggest more severe symptoms. These data were measured at home by self-report questionnaires. In this example, sleep duration in hours (*A*) was measured by the question “How long do you usually sleep on weekdays?" Observed confounders were body mass index (BMI; $L_B$) and bedtime ($L_A$), which was measured by the question “When do you usually go to bed on weekdays?" Because many adolescents reported no problems for all items on the SMFQ, the score distribution was positively skewed. In the present example, we focus on the clinical group comprising $N=416$ adolescents (13.1%) with SMFQ scores of 6 or higher during the study. Katon et al. (2008) reported 80% sensitivity and 81% specificity at this cutoff for diagnosis of major depression based on the Computerized Diagnostic Interview Schedule for Children (C-DISC). Missing data were primarily due to dropout. Of the 416 samples, 113 adolescents provided all three responses in the study. Descriptive statistics of sleep duration, SMFQ score, bedtime, and BMI are available in the Online Supplemental Material (Table S1).

In the first step, we use generalized least squares in the *lavaan* package to estimate the model parameters for each variable. To identify the measurement model (e.g., Eq. 16 for *Y*), SEM that assumes an AR(1) structure with time-varying autoregressive parameters and residual variances is specified for within-person variability scores of each variable. Let $P_i$ be the total number of variables observed in adolescent *i*. $P_i \times P_i$ weights $W_i$ are calculated from estimated parameters under the assumption of MAR. Within-person variability scores $X_i^*$ are then calculated using this weight and measurements $X_{i,\mathrm{obs}}$ for adolescent *i* as $\hat{X}_i^*=W^t_iX_{i,\mathrm{obs}}$.

Causal parameters ($\beta$ and $\gamma$) of sleep duration at 10 and 12 years old ($A^*_0$ and $A^*_1$) on later depressive symptoms (SMFQ scores $Y^*_1$ and $Y^*_2$) are estimated using calculated within-person variability scores by linear SNMM. In linear SNMM, blip functions and $U^*(\tau )$ are set as in Eqs. (26) and (28), except that the two confounders $L_A$ and $L_B$ are present in this example. When applying SNMMs, models $\mathcal {A}$ and $\mathcal {B}$ are both specified using first-order linear regression models. All calculated within-person variability scores were used in the analysis under the assumption of MAR.

We confirmed that the first step did not find improper solutions, and that current AR(1) models that assume time-varying parameters fit better than those that do not. Table S2 summarizes the model fit indices and estimated parameters in this step. All stable trait factor variance estimates are significant, indicating the necessity of controlling for stable traits. Specifically, the proportions of variances in measurements attributable to estimated stable trait factors at $k=0$ are 24.5%, 54.5%, 48.2%, and 74.8% for *Y*, *A*, $L_A$, and $L_B$, respectively.

Table 1 provides the estimation results of causal parameters, along with estimates based on the no-centering and observed-mean centering methods for comparison. As seen in Table 1, the proposed method reveals that intervention of longer sleep duration at 12 years old ($A^*_1$) has a positive effect (${\hat{\beta }}_{21}=-2.704$, 95%CI $[-4.938,-0.470]$, $p<$.05) on later depressive symptoms at 14 years old ($Y^*_2$) at the within-person level, but this estimate is not significant in the no-centering and observed mean score-centering methods. Similar positive effects of sleep duration were found in previous studies (Matamura et al., 2014), but the present analysis newly investigates this causal hypothesis at the within-person level by controlling for stable traits of persons. When the no-centering method is applied, the causal effect estimate of $A^*_0$ on $Y^*_1$ is significant, showing that intervention of longer sleep duration at 10 years old has a *negative* effect (${\hat{\beta }}_{10}=1.693$, 95% CI [0.405,2.981], $p<$.05) on later depressive symptoms at 12 years old. Considering that the magnitudes of the estimated stable trait factor variances were moderate or large for all variables, causal effect estimates in the no-centering method are unreliable and might be seriously biased.

In supplemental analyses, we confirmed that the major findings did not change even when using only data of adolescents who provided all three responses ($N=113$) and a different cutoff for SMFQ (Angold et al., 1995; Tables S2–S5). Again, statistical significance as well as sign and magnitude in estimates of causal parameters might change according to the choice of calculation (centering) methods for within-person variability scores, and ignoring the presence of stable traits of persons might lead to incorrect conclusions.

**Table 1:** Estimates of causal parameters of sleep duration on depression (SMFQ) ($N=416$)

	Proposed method	Observed-mean centering	Observed scores (no centering)
$\text {Sleep}_1 \rightarrow \text {SMFQ}_2\,(\beta _{21})$	$-$ **2.704 (1.140)**	0.095 (0.869)	$-$ 1.492 (1.080)
$(\text {Sleep}_1 \times \text {Bedtime}_1) \rightarrow \text {SMFQ}_2\, (\gamma _{21A})$	$-$ 0.603 (1.416)	0.916 (1.857)	0.336 (1.057)
$(\text {Sleep}_1 \times \text {BMI}_1) \rightarrow \text {SMFQ}_2 \,(\gamma _{21B})$	$-$ 0.442 (0.638)	$-$ 0.748 (1.169)	$-$ 0.532 (0.399)
$\text {Sleep}_0 \rightarrow \text {SMFQ}_2 \,(\beta _{20})$	$-$ 0.293 (1.179)	$-$ 0.309 (1.021)	0.185 (1.117)
$(\text {Sleep}_0 \times \text {Bedtime}_0) \rightarrow \text {SMFQ}_2 \,(\gamma _{20A})$	2.278 (1.918)	$-$ 3.012 (1.784)	1.169 (1.792)
$(\text {Sleep}_0 \times \text {BMI}_0) \rightarrow \text {SMFQ}_2 \,(\gamma _{20B})$	$-$ **1.856 (0.780)**	$-$ 0.251 (0.986)	0.306 (0.287)
$\text {Sleep}_0 \rightarrow \text {SMFQ}_1 \,(\beta _{10})$	0.702 (0.686)	0.279 (0.572)	**1.693 (0.657)**
$(\text {Sleep}_0 \times \text {Bedtime}_0) \rightarrow \text {SMFQ}_1 \,(\gamma _{10A})$	0.315 (1.177)	1.037 (1.069)	$-$ 0.918 (0.920)
$(\text {Sleep}_0 \times \text {BMI}_0) \rightarrow \text {SMFQ}_1 \,(\gamma _{10B})$	0.021 (0.473)	0.773 (0.572)	$-$ 0.101 (0.212)

Bold font indicates statistical significance.

## General Discussion

We proposed a two-step estimation method for within-person variability score-based causal inference to estimate joint effects of time-varying (continuous) treatments/predictors by controlling for stable traits. In the first step, a within-person variability score for each person, which is disaggregated from the stable trait factor score, is calculated using weights based on the best linear correlation preserving predictor through SEM. Causal parameters are then estimated by MSMs or SNMs, using calculated within-person variability scores. The proposed method can be viewed as one that synthesizes the two traditions of factor analysis/SEM in psychometrics and a method of causal inference (MSMs or SNMs) in epidemiology.

In this paper, we began by providing formal definitions of stable trait factors (for between-person relations) and within-person variability scores (for within-person relations), because these concepts have not been fully characterized in the causal inference literature despite the fact that they have been attracting increasing attention in psychometrics and behavioral science (e.g., Hamaker et al., 2015; Usami et al., 2019a). On the other hand, in epidemiology the conceptual and mathematical differences between stable trait factors and accumulating factors, along with which kind of time-invariant factor is included in each statistical model, have received less attention. This paper may help bridge the gap. We have also clarified the assumptions required to identify causal parameters for within-person variability score-based causal inference: (i) (as depicted in Fig. 1b) measurements are expressed by the linear sum of stable trait factor scores (defined as Eq. 4) and within-person variability scores (defined as Eq. 5) that are mutually uncorrelated, (ii) within-person variability scores are expressed by functions of those (with assumption of homogeneity) in past time, (iii) consistency, (iv) sequential ignorability, (v) SUTVA, (vi) positivity, (vii) modularity, and (viii) multivariate normality (if MLE is used in the first step).

As for the second assumption, our approach is more flexible than the RI-CLPM (that includes time-varying observed confounders), which researchers are becoming increasingly interested in for uncovering within-person relations among variables, in that the assumption of linearity is not required with respect to time-varying observed confounders at the within-person level. We particularly emphasize the utility of SNMs with G-estimation, because of its property of being doubly robust to the model misspecifications in how the time-varying observed confounders are functionally related to treatments/predictors and outcomes, along with flexibility in that it allows investigation of moderation effects of treatments with observed confounders.

Through simulation and empirical application, we illustrated that ignoring the presence of stable traits might lead to incorrect conclusions in causal effects. We also confirmed that the proposed approach is superior to observed-mean centering, as a conventional method to predict stable traits of persons. Especially when *K* is small, observed-mean centering showed serious negative biases in estimates of causal parameters. Considering that most research applying the RI-CLPM to uncover within-person relations used longitudinal data with two or three time points ($K=1,2$; e.g., Usami et al., 2019b), observed-mean centering cannot be recommended.

A recent study provided closed-form parametric expressions of causal effects for linear models (Gische et al., 2021), and Gische and Voelkle (in press) proposed asymptotically efficient estimators in the case of ML estimation. It is suggested that, at least in large samples, the parametric procedure proposed by Gische and Voelkle (in press) may give smaller standard errors compared with the proposed method in simulations in which data are generated by a linear model with normal residuals. Comparing the performances of these methods and the RI-CLPM under various conditions that account for nonlinear relations among variables is an important topic for future studies.

One caveat for the proposed method, which is relevant to the second assumption above, is that in the first step, one must correctly specify the structure (such as the AR(1) structure) for within-person variability scores in each variable so that the (identified) SEM can yield consistent estimates of parameters, which are required for consistent estimation of causal effects in the subsequent step. However, in general, how to establish the correct (or even a plausible) DAG model is a major challenge (Hamaker et al., 2020; see also the discussion in Sect. 2.4). Relatedly, the premise that stable trait factors exist and loadings from factors are equal to those in the DGP might be restrictive in actual applications; therefore, we need to carefully account for the consequences of possible model misspecifications in the first step on the results in the second step to precisely infer within-person relations. The good news is that in the present simulation, we confirmed that the specified time-varying AR(1) structure works well to recover causal parameters, and its performance was not largely influenced even when there were model misspecifications (i.e., time-varying effects from time-invariant factors). However, additional large-scale simulations to further clarify the robustness of the method regarding this point are needed in future studies.

We can also use model fit indices to evaluate how well the structure specified in the first step fits to the data, especially when the number of time points is large. However, this procedure is not a fundamental solution. Even if a researcher is certain that SEM that assumes stable trait factors for each variable can be specified in the first step, in general there is still at great risk of violating some assumptions. Notably, relating to the first assumption above, stable trait factors and within-person variability scores (temporal deviations) at each time point might be correlated if they share common causes. Future studies should investigate how this violation impacts the estimated causal parameters.

We used two-step estimation to account for feasibility, but this issue remains in that one still must write programming code, as that in the Online Supplemental Material. We are planning to develop packages for the proposed method. Another potential limitation is that the proposed approach (as well as the RI-CLPM) demands longitudinal data with three or more time points ($K\ge 2$) to identify the measurement model (SEM) in the first step unless strong parameter constraints are imposed.

Because we take an SEM approach in the first step, accounting for measurement errors, which is closely related to violation of the consistency assumption, is feasible under the parametric assumption. Although we expect that longitudinal data with large *K* are required for precisely estimating measurement-error variances, we plan to investigate how the proposed method works under measurement models that include measurement errors.

This paper opens a new avenue for exploring other various research questions that are closely relevant to causal hypotheses. For example, use of within-person variability scores can be extended to cases in which one is interested in uncovering reciprocal effects (e.g., Usami et al., 2019a) and mediation effects (e.g., Goldsmith et al., 2018; Tchetgen & Shpitser, 2012), as well as to multilevel modeling and hierarchical continuous time modeling (Driver & Voelkle, 2018). Note that there is still room for discussion on the issues of within-person relation and stable traits, as well as the issue about when and how to include (time-invariant) factors in the assumed DGP (see Sect. 2.4). The present paper is intended to promote substantial discussion about the conceptual and statistical properties of the time-invariant factors (e.g., stable trait factors or accumulating factors) included in the assumed DGP among researchers who wish to infer within-person relations and causality, and the hope is that the proposed method helps in exploring various causal hypotheses in longitudinal design and guiding better decision-making for researchers.

### Supplementary Information

Below is the link to the electronic supplementary material.

Supplementary file 1 (pdf 1259 KB)

## Footnotes

1 Time-invariant observed confounders $L_i$ can be included as a special case, but in the DGPs discussed herein, only time-varying observed confounders $L_{ik}$ are assumed for simplicity.

2 *Y* is not shown explicitly here because it is part of *L*. We often omit *Y* in expressing time-varying observed confounders in this paper.

3 Here, the path $a^*_0 \rightarrow a^*_1 \rightarrow Y^*_2$ does *not* need to be accounted for because time-varying treatments are now intervened and $a^*_1$ does not depend on $a^*_0$).

4 Although we defined stable trait factors as the (time-invariant) difference between the expected value of a given person’s measurement and the temporal group mean, one could argue for another definition that allows for time-varying influences on measurements. If this is the case, time-varying factor loadings can be freely specified in this step (except for one fixed factor loading for identification). However, there may be some cost in that the minimum number of time points required to identify the measurement model becomes larger than that in specifying time-invariant loadings.

5 Here, an intercept becomes zero. Other terms such as quadratic effects (e.g., $A^{*2}_{ik}$) for time-varying treatments can be included in MSMs. Also, one can include observed covariates/nonconfounders to assess effect modification (Hernán & Robins, 2021).

6 In this paper, we use the term *stationarity assumption* to indicate invariance of autoregressive parameters, cross-lagged parameters, and residual variance parameters over time, rather than indicating means and (co)variances in variables to be invariant over time.

7 As a similar problem, the risk of using observed person-specific (or cluster-specific) means to express cluster effects is recognized as Nickell’s bias and Lüdtke’s bias for estimates of regression coefficients in applications of multilevel models (e.g., Asparouhov & Muthén, 2018; Lüdtke et al., 2008; McNeish & Hamaker, 2020; Usami, 2017).
