# Erratum to: Within-Person Variability Score-Based Causal Inference: A Two-Step Estimation for Joint Effects of Time-Varying Treatments

**DOI:** 10.1007/s11336-022-09890-6

**Published:** 2022-10-19

**Authors:** Satoshi Usami

**Affiliations:** Department of Education, University of Tokyo, Tokyo, Japan

Correction to: Psychometrika 10.1007/s11336-022-09879-1

The original version of the article contains the below listed errors. The following transcription errors have been corrected:

1. Second line in Eq. (15): “${\bar{A}^*_{i(k-1)}}\bar{a}^*_{i(k-1)}+1$” has been changed to “${\bar{A}^*_{i(k-1)}}=\bar{a}^*_{i(k-1)}\,{+}\,1$”.
2. below Eq. (23): “$\tau =(\beta _{k0}, \beta _{k1}, \dots , \beta _{K(K-1)})^t$” has been changed to “$\tau =(\beta _{k0}, \beta _{k1}, \dots , \beta _{k(k-1)})^t$”.
3. Second line in Eq. (27): “$\bar{A}^*_{i(k-2)}\bar{a}^*_{i(k-2)}$” has been changed to “$\bar{A}^*_{i(k-2)}=\bar{a}^*_{i(k-2)}$”. Also, the equation number “(27)” was moved to the second line.

