# Supplementary material for: Within-Person Variability Score-Based Causal Inference: A Two-Step Estimation for Joint Effects of Time-Varying Treatments
Source: Psychometrika. 2022 Aug 18;88(4):1466–94. doi: 10.1007/s11336-022-09879-1 (PMC10656338; doi:10.1007/s11336-022-09879-1)
Supplement: Supplementary file 1 — (pdf 1259 KB) [file 11336_2022_9879_MOESM1_ESM.pdf]

Online Supplemental Material

Tables .... p. 2
Figures .... p. 7
Derivation of weights matrix W .... p. 22
Simulation codes .... p. 25

Table S1: Means (SDs) of variables in the dataset.

	SMFQ	Sleep duration (hrs)	Bedtime (pm)	BMI
$k = 0$ (year10)	10.72 (4.13)	8.96 (0.65)	9:51 (40 min)	17.20 (2.83)
$k = 1$ (year12)	11.26 (4.67)	8.41 (0.80)	10:25 (49 min)	18.63 (3.15)
$k = 2$ (year14)	11.72 (4.99)	7.62 (0.95)	11:19 (57 min)	20.50 (3.32)

Table S2: Parameter estimates (SEs) and model fit indices at step 1 ($K = 2$).

	SMFQ (Y)	Sleep duration (A)	Bed time (L_A)	BMI (L_B)
Number of parameters	6	6	6	6
Degree of freedom	0	0	0	0
CFI	1	1	1	1
RMSEA	0	0	0	0
SRMR	0	0	0	0
Regression coefficient $Y_0^* \rightarrow Y_1^*$	-0.112 (0.161)	0.143 (0.254)	0.581 (0.180)	0.543 (0.264)
Regression coefficient $Y_1^* \rightarrow Y_2^*$	-0.046 (0.146)	0.208 (0.136)	0.357 (0.124)	0.484 (0.228)
$V(Y_0^*)$	15.928 (2.812)	0.224 (0.061)	0.268 (0.082)	2.919 (1.132)
Residual variances of Y_1^*	17.733 (3.716)	0.385 (0.072)	0.394 (0.048)	1.967 (0.565)
Residual variances of Y_2^*	21.448 (3.419)	0.632 (0.075)	0.601 (0.070)	1.486 (0.389)
$V(I)$	5.158 (1.977)	0.268 (0.064)	0.249 (0.083)	8.683 (1.692)

Note: Model fits are perfect because saturated models are used.

Table S3: Causal effect estimates (SEs) of sleep duration on depression (SMFQ) (N=113).
(SMFQ cutoff = 6; Complete data by listwise deletion)

	Proposed method	Observed-mean centering	Observed scores (no centering)
$Sleep_1 \rightarrow SMFQ_2 \quad (\beta_{21})$	**-6.013 (1.523)**	0.175 (0.881)	-1.695 (1.110)
$(Sleep_1 \times Bedtime_1) \rightarrow SMFQ_2 \quad (\gamma_{21A})$	0.914 (2.744)	0.983 (1.910)	0.432 (1.127)
$(Sleep_1 \times BMI_1) \rightarrow SMFQ_2 \quad (\gamma_{21B})$	**-1.482 (0.544)**	-0.641 (1.187)	-0.568 (0.414)
$Sleep_0 \rightarrow SMFQ_2 \quad (\beta_{20})$	**-5.016 (1.950)**	-0.544 (1.049)	0.412 (1.166)
$(Sleep_0 \times Bedtime_0) \rightarrow SMFQ_2 \quad (\gamma_{20A})$	**19.127 (5.241)**	-2.255 (1.791)	**6.612 (1.799)**
$(Sleep_0 \times BMI_0) \rightarrow SMFQ_2 \quad (\gamma_{20B})$	-0.159 (0.575)	-0.571 (0.894)	0.110 (0.311)
$Sleep_0 \rightarrow SMFQ_1 \quad (\beta_{10})$	1.943 (1.589)	0.515 (1.026)	1.060 (1.109)
$(Sleep_0 \times Bedtime_0) \rightarrow SMFQ_1 \quad (\gamma_{10A})$	-0.533 (4.272)	0.657 (1.751)	-0.889 (1.711)
$(Sleep_0 \times BMI_0) \rightarrow SMFQ_1 \quad (\gamma_{10B})$	0.034 (0.469)	1.161 (0.874)	-0.040 (0.296)

Note: Bold font indicates statistical significance.

Table S4: Causal effects estimates (SEs) of sleep duration on depression (SMFQ) (N=254). (SMFQ cutoff = 8)

	Proposed method	Observed-mean centering	Observed scores (no centering)
$Sleep_1 \rightarrow SMFQ_2 \quad (\beta_{21})$	-	0.646 (1.000)	-1.803 (1.065)
$(Sleep_1 \times Bedtime_1) \rightarrow SMFQ_2 \quad (\gamma_{21A})$	-	1.390 (2.416)	-0.840 (0.910)
$(Sleep_1 \times BMI_1) \rightarrow SMFQ_2 \quad (\gamma_{21B})$	-	-0.745 (1.763)	-0.045 (0.430)
$Sleep_0 \rightarrow SMFQ_2 \quad (\beta_{20})$	-	-0.639 (1.199)	-0.884 (1.191)
$(Sleep_0 \times Bedtime_0) \rightarrow SMFQ_2 \quad (\gamma_{20A})$	-	0.447 (2.525)	1.915 (1.775)
$(Sleep_0 \times BMI_0) \rightarrow SMFQ_2 \quad (\gamma_{20B})$	-	-0.541 (1.060)	-0.012 (0.246)
$Sleep_0 \rightarrow SMFQ_1 \quad (\beta_{10})$	-	0.668 (0.627)	**1.802 (0.916)**
$(Sleep_0 \times Bedtime_0) \rightarrow SMFQ_1 \quad (\gamma_{10A})$	-	0.169 (1.525)	-0.911 (1.317)
$(Sleep_0 \times BMI_0) \rightarrow SMFQ_1 \quad (\gamma_{10B})$	-	0.770 (0.695)	-0.148 (0.240)

Note: Hyphens indicate estimates were not obtained because of nonconvergence.

Table S5: Causal effects estimates (SEs) of sleep duration on depression (SMFQ) (N=60).
(SMFQ cutoff = 8; Complete data by listwise deletion)

	Proposed method	Observed-mean centering	Observed scores (no centering)
$Sleep_1 \rightarrow SMFQ_2 \quad (\beta_{21})$	**-4.012 (1.057)**	-0.418 (1.035)	-2.056 (1.120)
$(Sleep_1 \times Bedtime_1) \rightarrow SMFQ_2 \quad (\gamma_{21A})$	1.153 (1.573)	-0.964 (2.571)	-1.074 (1.016)
$(Sleep_1 \times BMI_1) \rightarrow SMFQ_2 \quad (\gamma_{21B})$	0.017 (0.579)	-1.415 (1.778)	0.110 (0.504)
$Sleep_0 \rightarrow SMFQ_2 \quad (\beta_{20})$	**-5.258 (1.561)**	-1.426 (1.127)	-0.088 (1.255)
$(Sleep_0 \times Bedtime_0) \rightarrow SMFQ_2 \quad (\gamma_{20A})$	2.246 (3.087)	-1.139 (2.445)	**6.360 (2.112)**
$(Sleep_0 \times BMI_0) \rightarrow SMFQ_2 \quad (\gamma_{20B})$	0.443 (0.475)	-0.255 (0.869)	-0.264 (0.319)
$Sleep_0 \rightarrow SMFQ_1 \quad (\beta_{10})$	2.179 (1.782)	1.125 (1.149)	1.476 (1.402)
$(Sleep_0 \times Bedtime_0) \rightarrow SMFQ_1 \quad (\gamma_{10A})$	0.035 (3.524)	2.065 (2.493)	0.532 (2.359)
$(Sleep_0 \times BMI_0) \rightarrow SMFQ_1 \quad (\gamma_{10B})$	-0.081 (0.543)	0.702 (0.886)	-0.165 (0.357)

Note: Bold font indicates statistical significance.

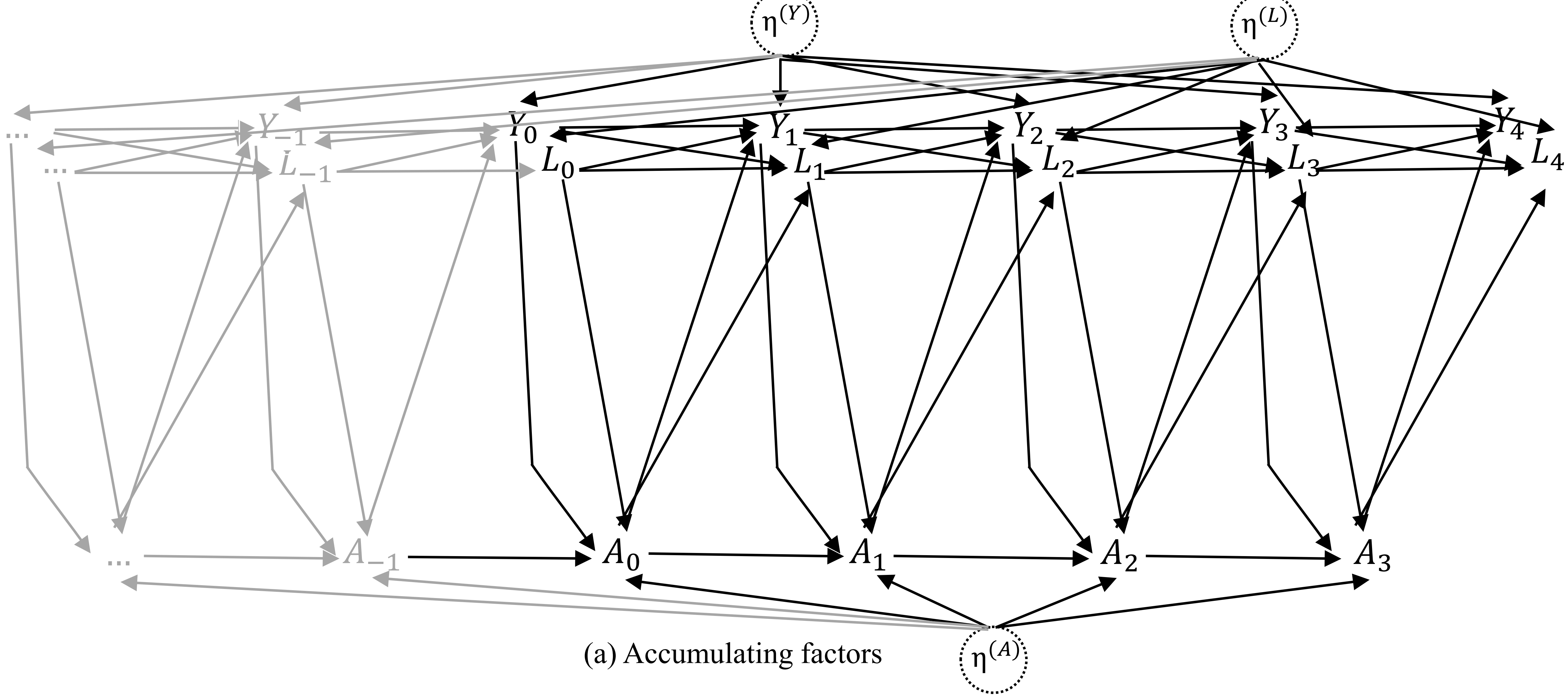

(a) Accumulating factors

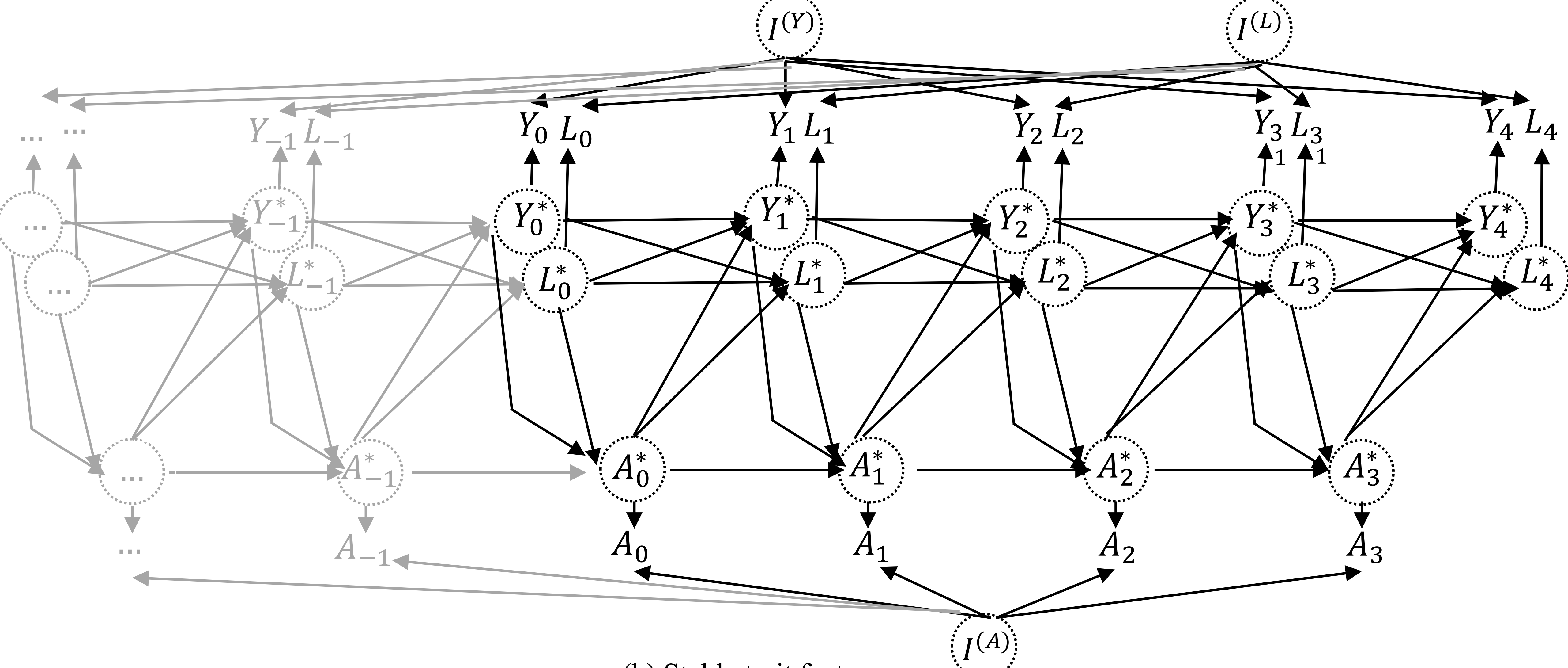

(b) Stable trait factors

Figure S1: The causal diagram (DAG) that extends Figure 1 to account for cases in which the process starts prior to the initial measurements. Measurements that are observed in the study are drawn in black, whereas the unobserved past of the process is drawn in gray. In (a), causal effects from time-invariant factors (η: accumulating factors) prior to the initial measurements transmit to the future measurements, and thus initial measurements must somehow account for the impact from this factor in past of the process (e.g., Gische et al., 2021). However, this point is irrelevant to (b), in which time-invariant factors (I: stable trait factors) have only direct effects on measurements and the effects do *not* transmit to the future measurements.

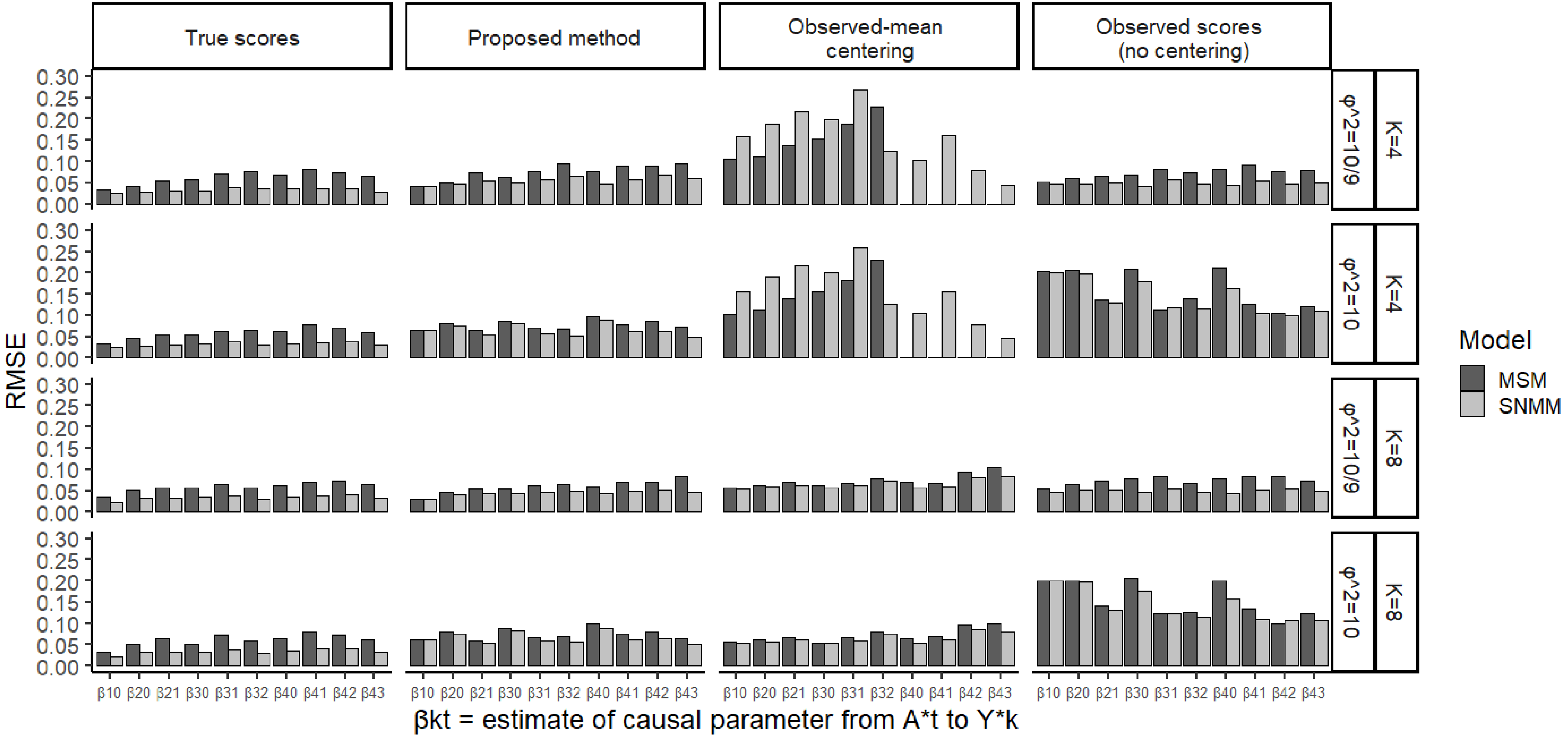

Figure S2. RMSEs of causal effect estimates ($N = 1{,}000$)

Note: Because of rank deficiency, in K=4 no estimates of $\beta_{40}, \beta_{41}, \beta_{42}, \beta_{43}$ are available in the marginal structural model with observed-mean centering.

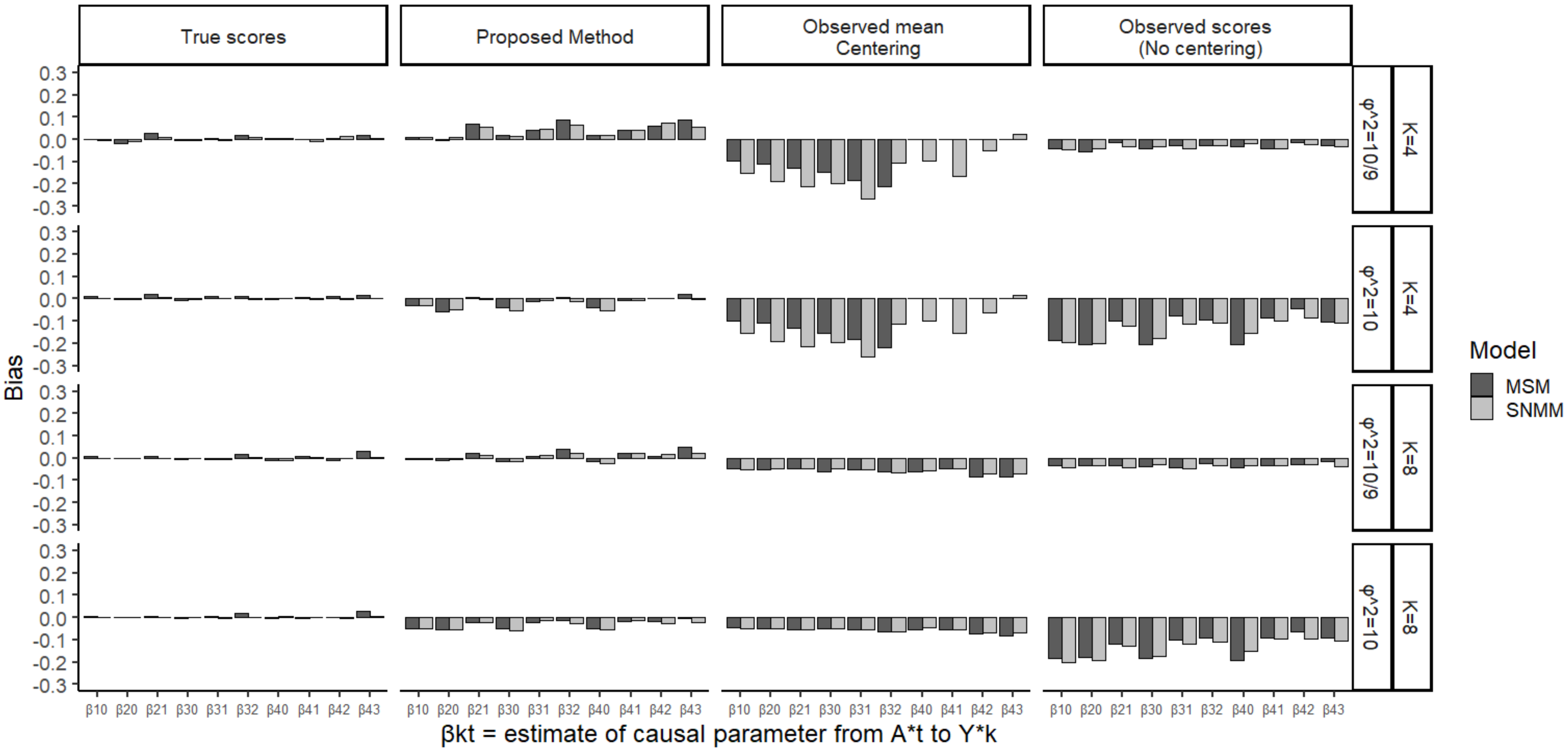

Figure S3. Biases of causal effect estimates ($N = 200$)

Note: Because of rank deficiency, in K=4 no estimates of $\beta_{40}, \beta_{41}, \beta_{42}, \beta_{43}$ are available in the marginal structural model with observed-mean centering.

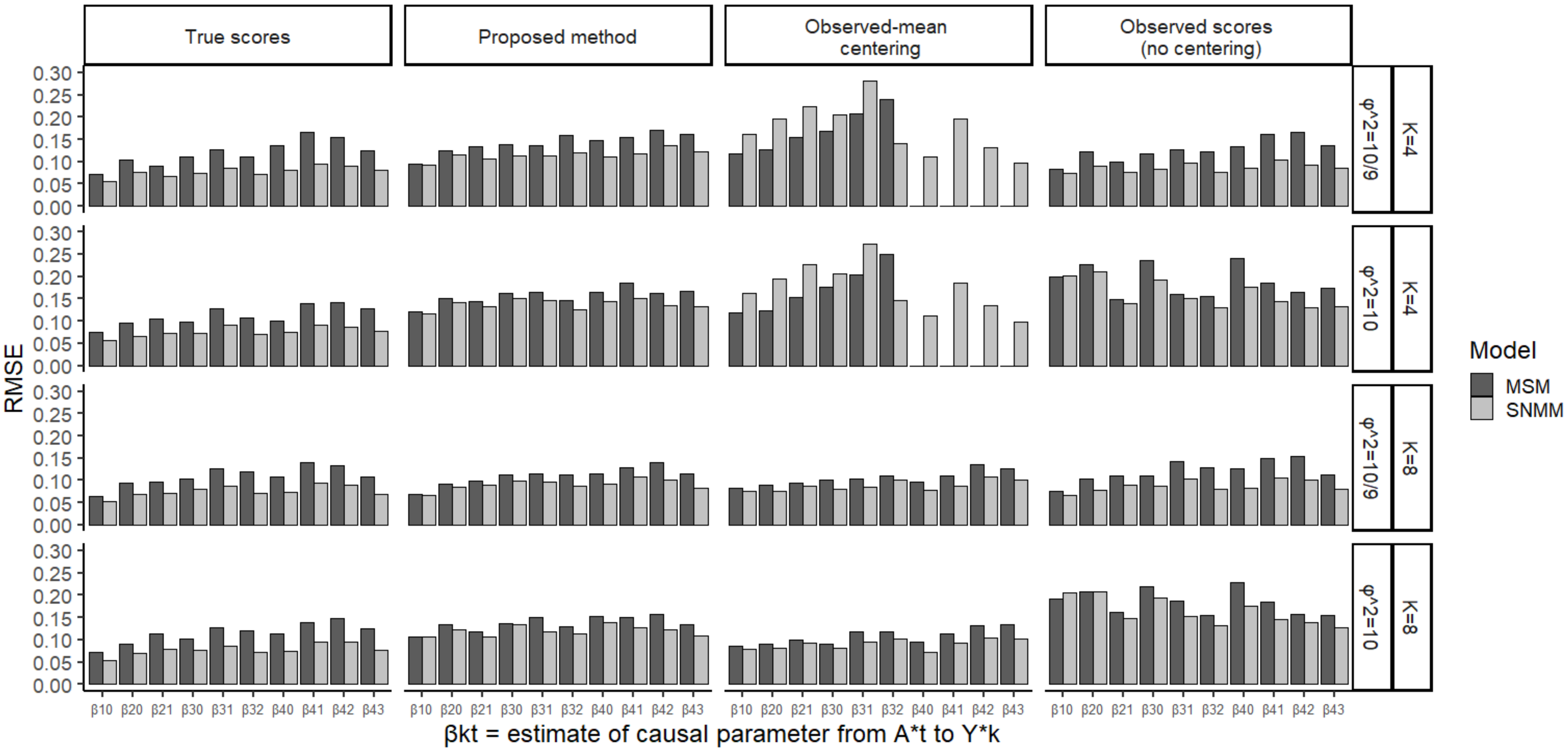

Figure S4. RMSEs of causal effect estimates ($N = 200$)

Note: Because of rank deficiency, in *K*=4 no estimates of $\beta_{40}, \beta_{41}, \beta_{42}, \beta_{43}$ are available in the marginal structural model with observed-mean centering.

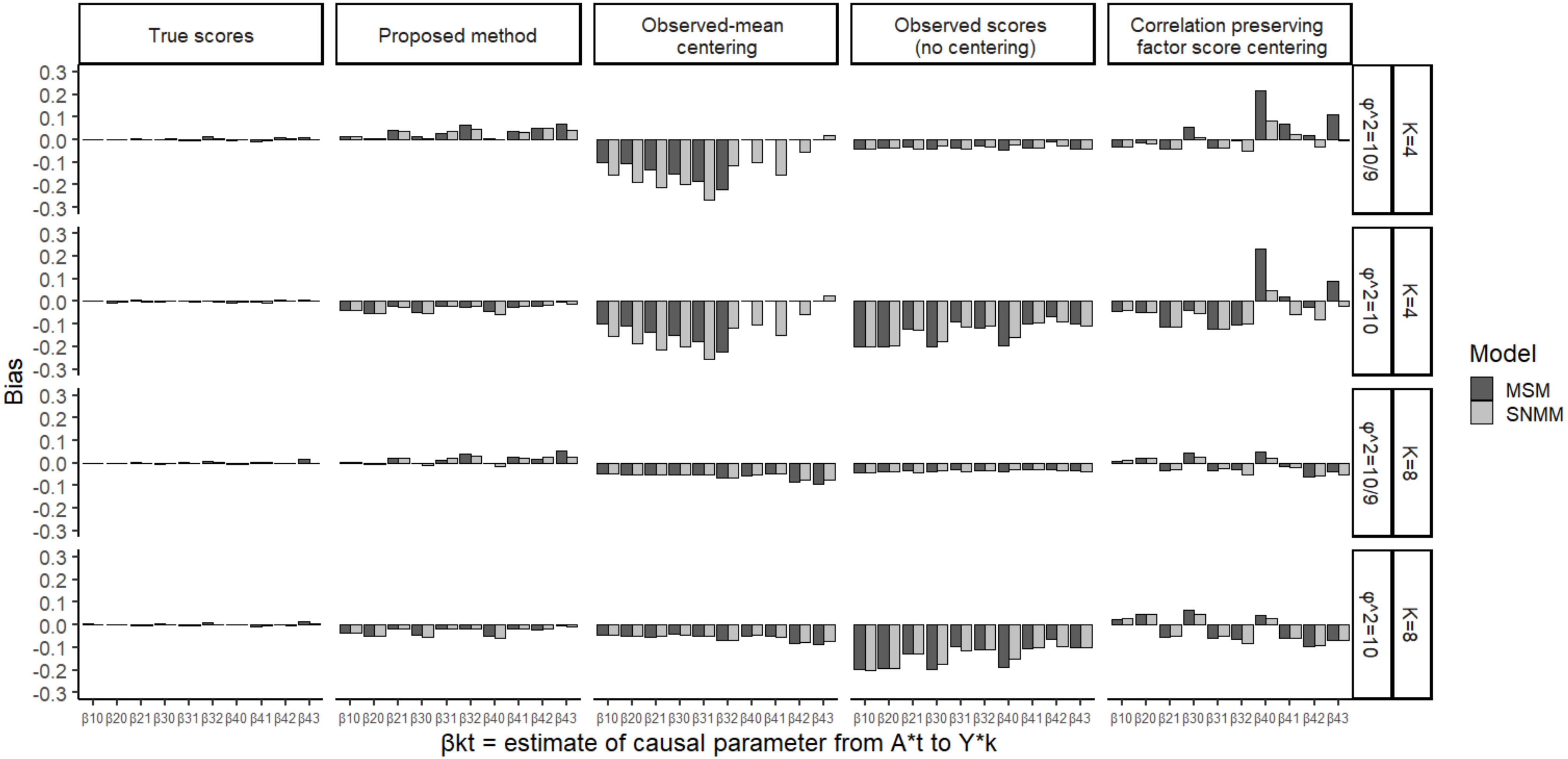

Figure S5. Biases of causal effect estimates that include the condition of correlation preserving factor score centering ($N = 1{,}000$)

Note: Because of rank deficiency, in *K*=4 no estimates of $\beta_{40}, \beta_{41}, \beta_{42}, \beta_{43}$ are available in the marginal structural model with observed-mean centering.

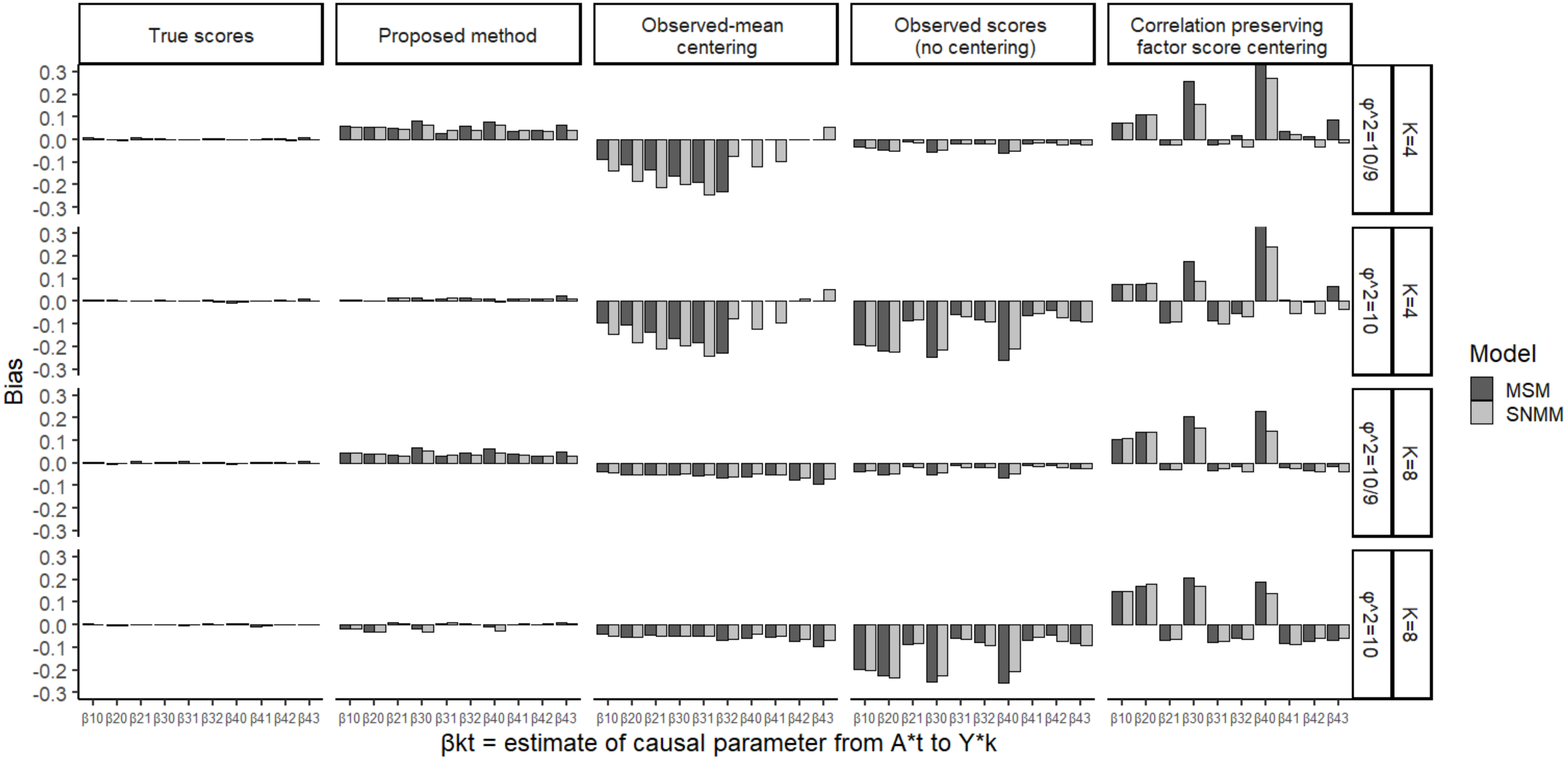

Figure S6. Biases of causal effect estimates ($N = 1{,}000$, variance of residuals = 10)

Note: Because of rank deficiency, in K=4 no estimates of $\beta_{40}, \beta_{41}, \beta_{42}, \beta_{43}$ are available in the marginal structural model with observed-mean centering.

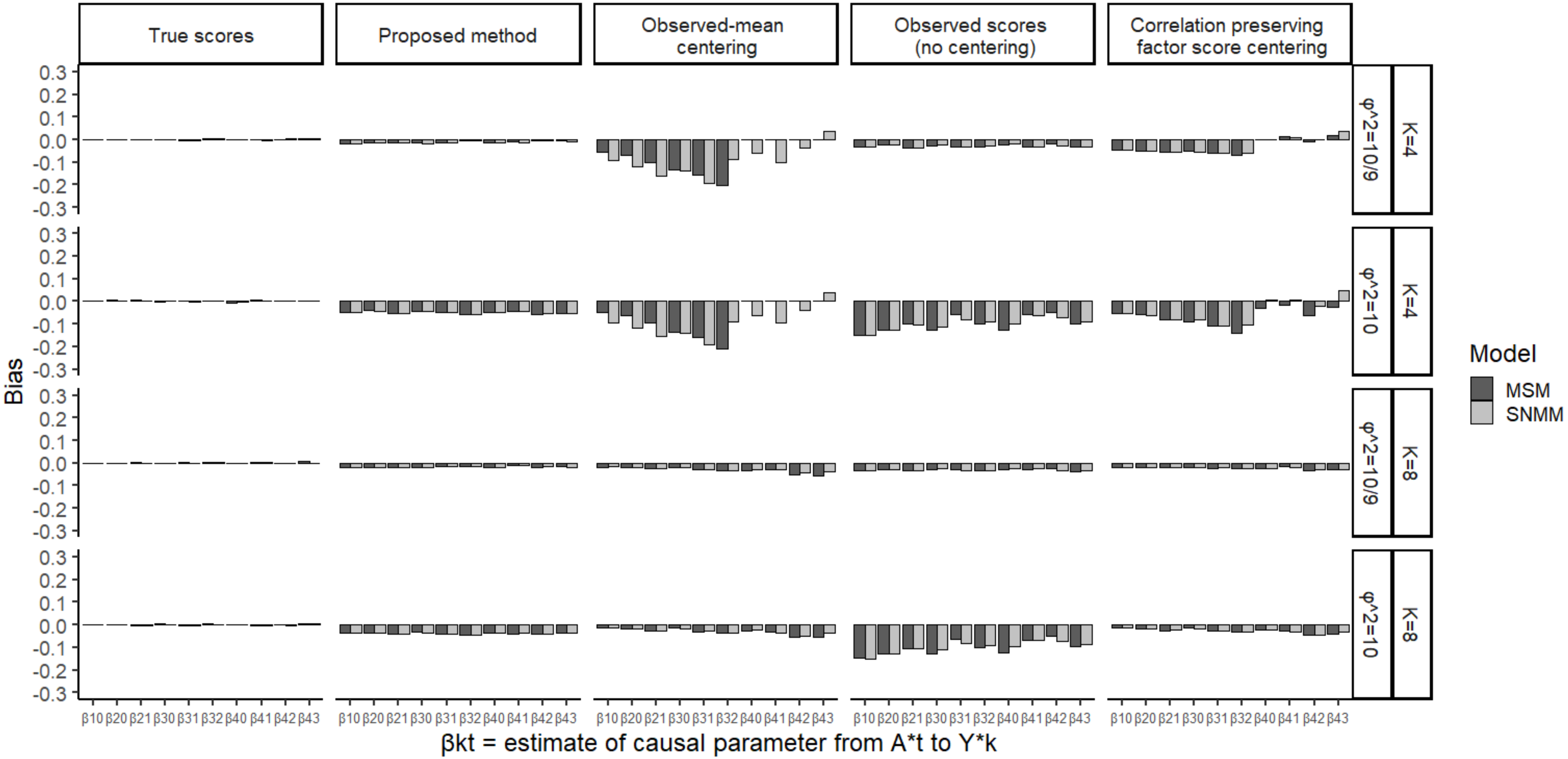

Figure S7. Biases of causal effect estimates ($N = 1{,}000$, variance of residuals = 5, Y_1^*=0.2Y_0^*+0.3A_0^*+0.2*L_0^*+$d(Y)$, A_1^*<-0.2Y_0^*+0.3A_0^*+0.2L_0^* + $d(A)$, L_1^*<-0.2Y_0^*+0.2A_0^*+0.4L_0^* + $d(L)$, proportions of variances explained in Equation (32) are almost 30%)

Note: Because of rank deficiency, in *K*=4 no estimates of $\beta_{40}, \beta_{41}, \beta_{42}, \beta_{43}$ are available in the marginal structural model with observed-mean centering.

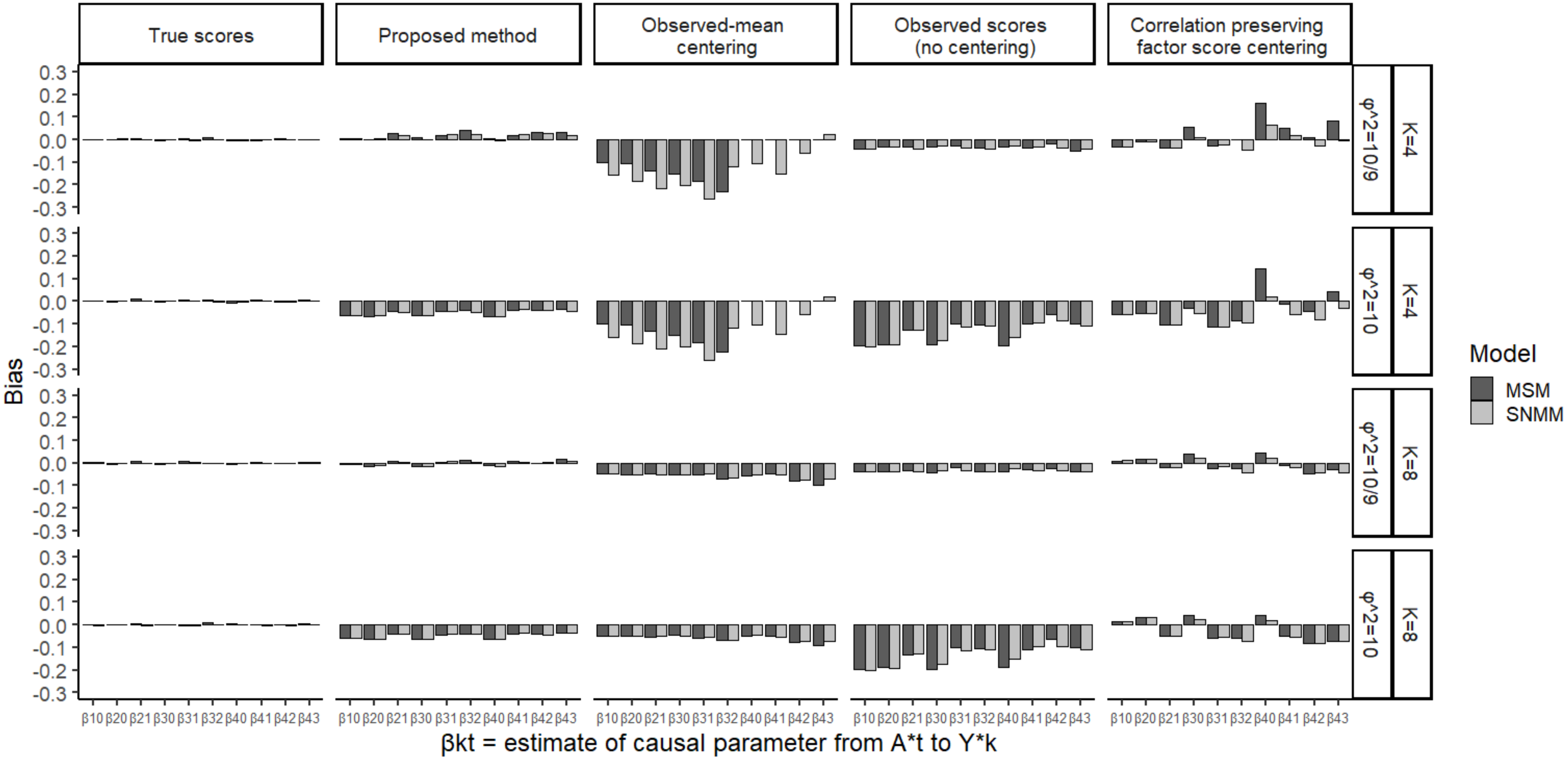

Figure S8. Biases of causal effect estimates ($N = 1{,}000$, AR(2) effects are included in step 1)

Note: Because of rank deficiency, in *K*=4 no estimates of $\beta_{40}, \beta_{41}, \beta_{42}, \beta_{43}$ are available in the marginal structural model with observed-mean centering.

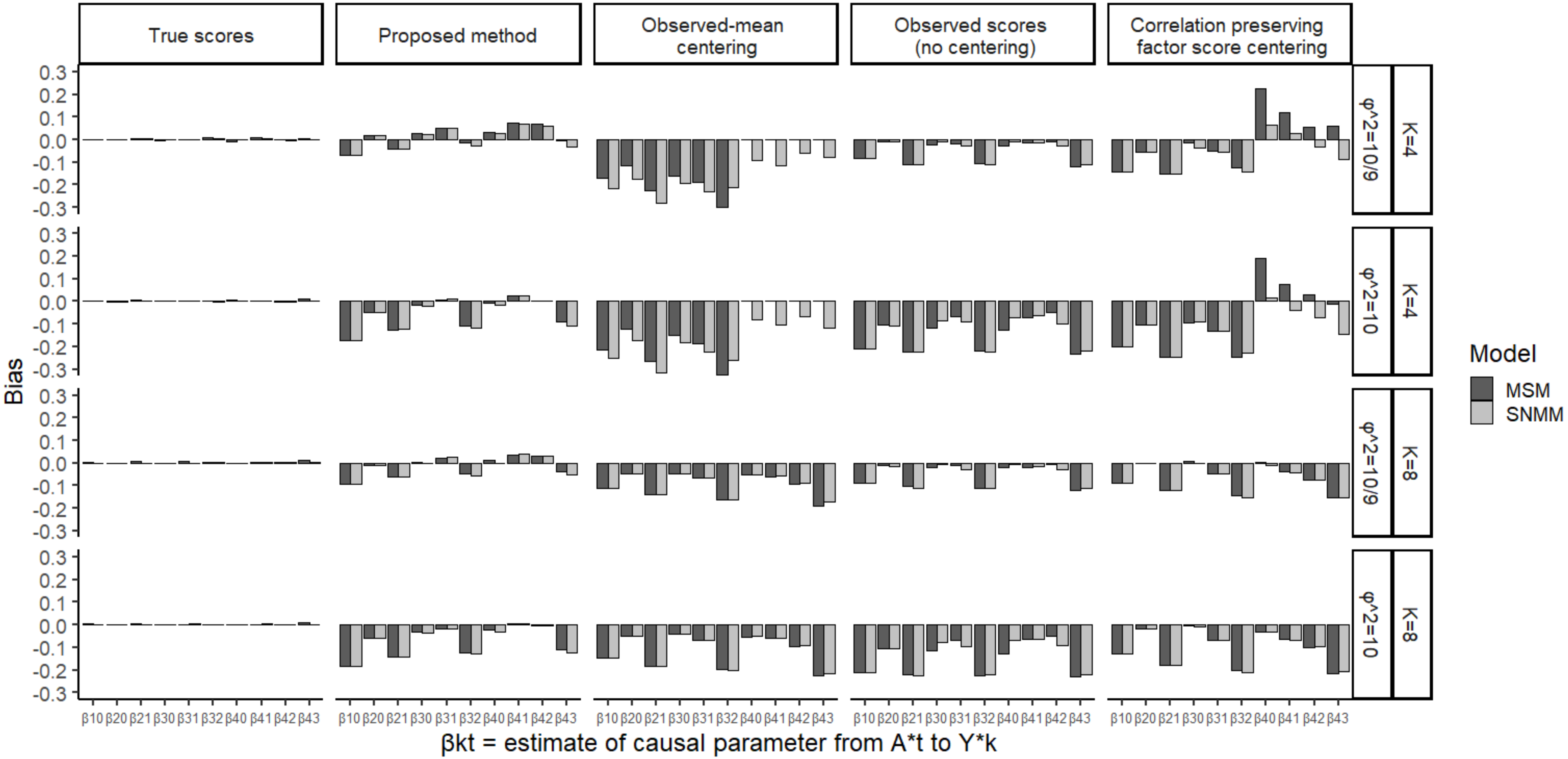

Figure S9. Biases of causal effect estimates ($N = 1{,}000$, when measurement errors [whose variances are equal to 20% of those of the initial measurements] are present)

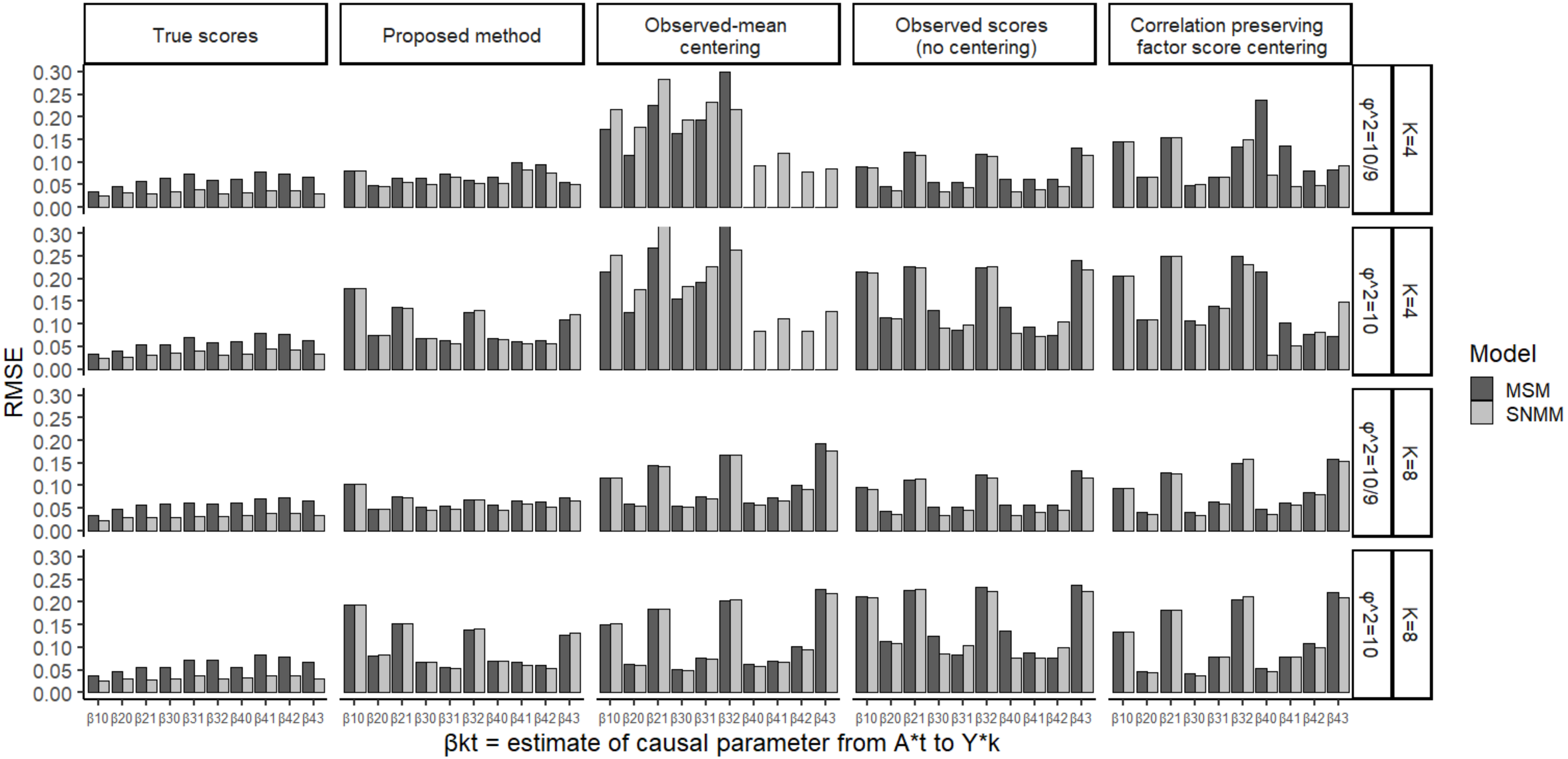

Figure S10. RMSEs of causal effect estimates ($N = 1{,}000$, when measurement errors [whose variances are equal to 20% of those of the initial measurements] are present)

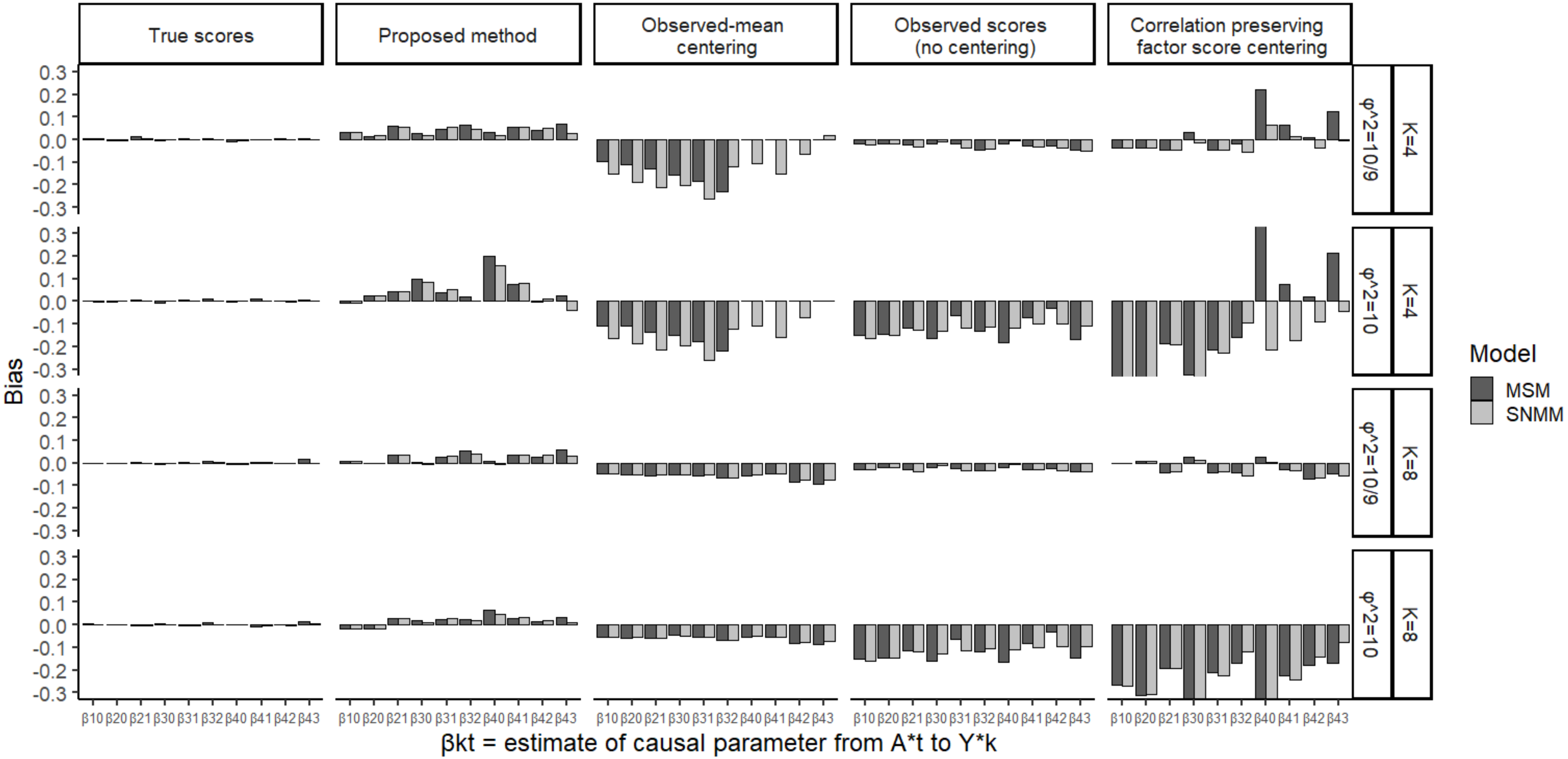

Figure S11. Biases of causal effect estimates ($N = 1{,}000$, time-invariant factors do not influence measurements as stable trait factors)

Note: The relation between outcomes and time-invariant factors (I) is set as $Y_{ik} = \left(1 + \frac{0.5k}{K}\right) I_i^{(Y)} + 0.3 I_i^{(A)} + 0.3 I_i^{(L)} + Y_{ik}^{*}$.

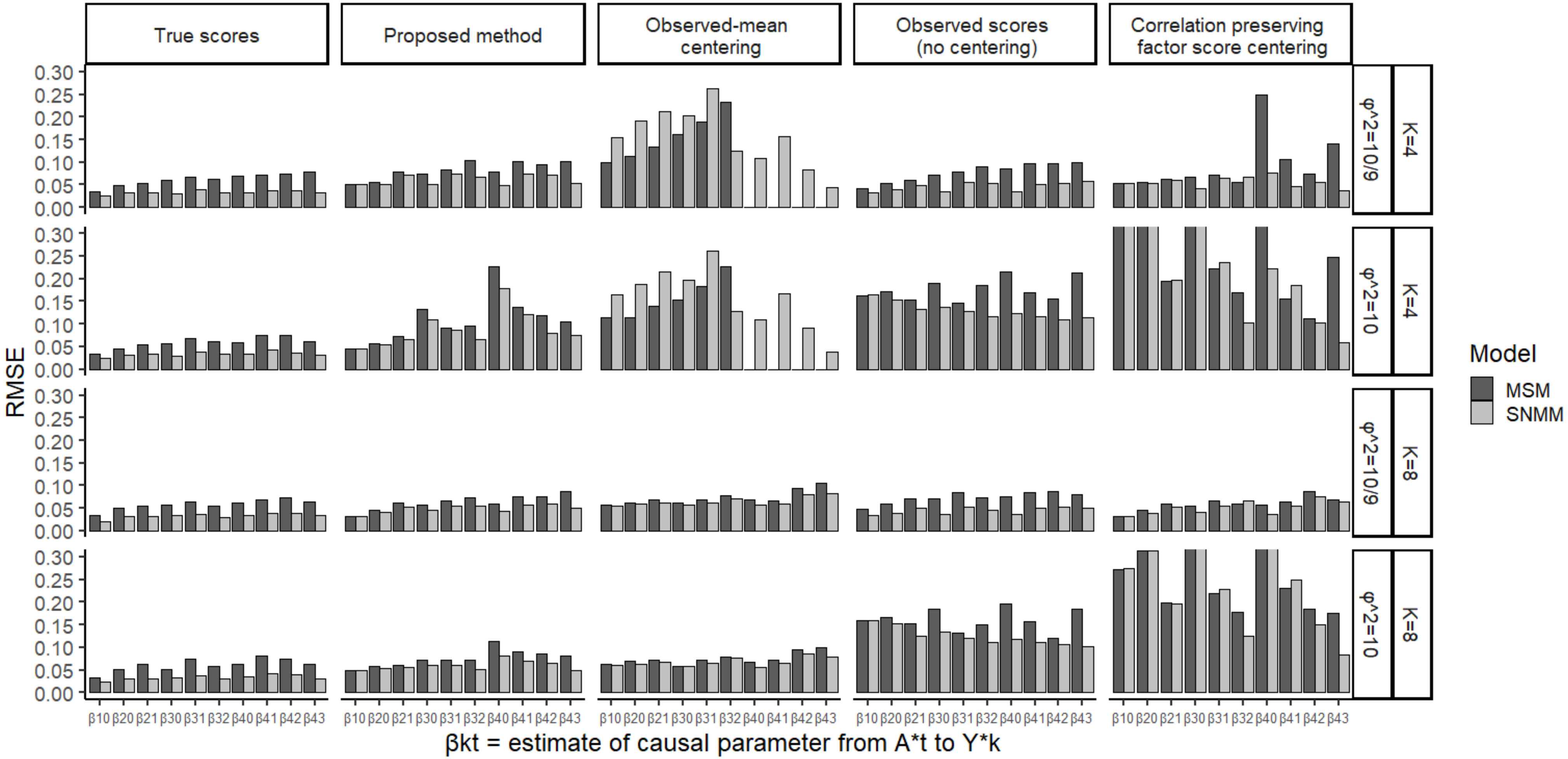

Figure S12. RMSEs of causal effect estimates ($N = 1{,}000$, time-invariant factors do not influence measurements as stable trait factors)

Note: The relation between outcomes and time-invariant factors (I) is set as $Y_{ik} = \left(1 + \frac{0.5k}{K}\right) I_i^{(Y)} + 0.3 I_i^{(A)} + 0.3 I_i^{(L)} + Y_{ik}^{*}$.

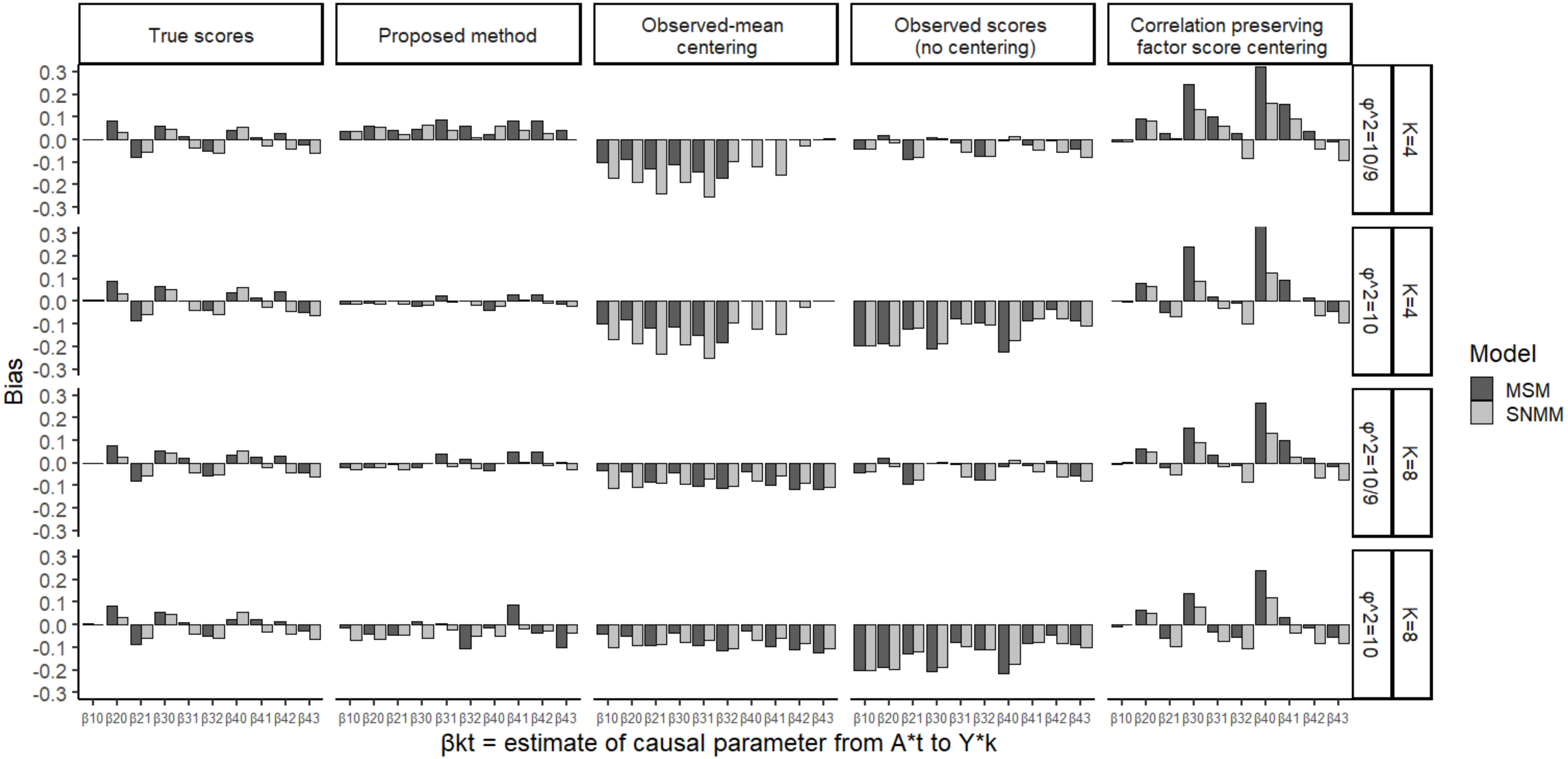

Figure S13. Biases of causal effect estimates ($N = 1{,}000$, quadratic effects of time-varying observed confounders are present in the treatment assignment model)

Note: Quadratic effects from time-varying observed confounders are included as $A^*_{ik} = 0.20Y^*_{ik} + 0.08Y^{*2}_{ik} + 0.40A^*_{i(k-1)} + 0.30L^*_{ik} + d^{(A)}_{ik}$.

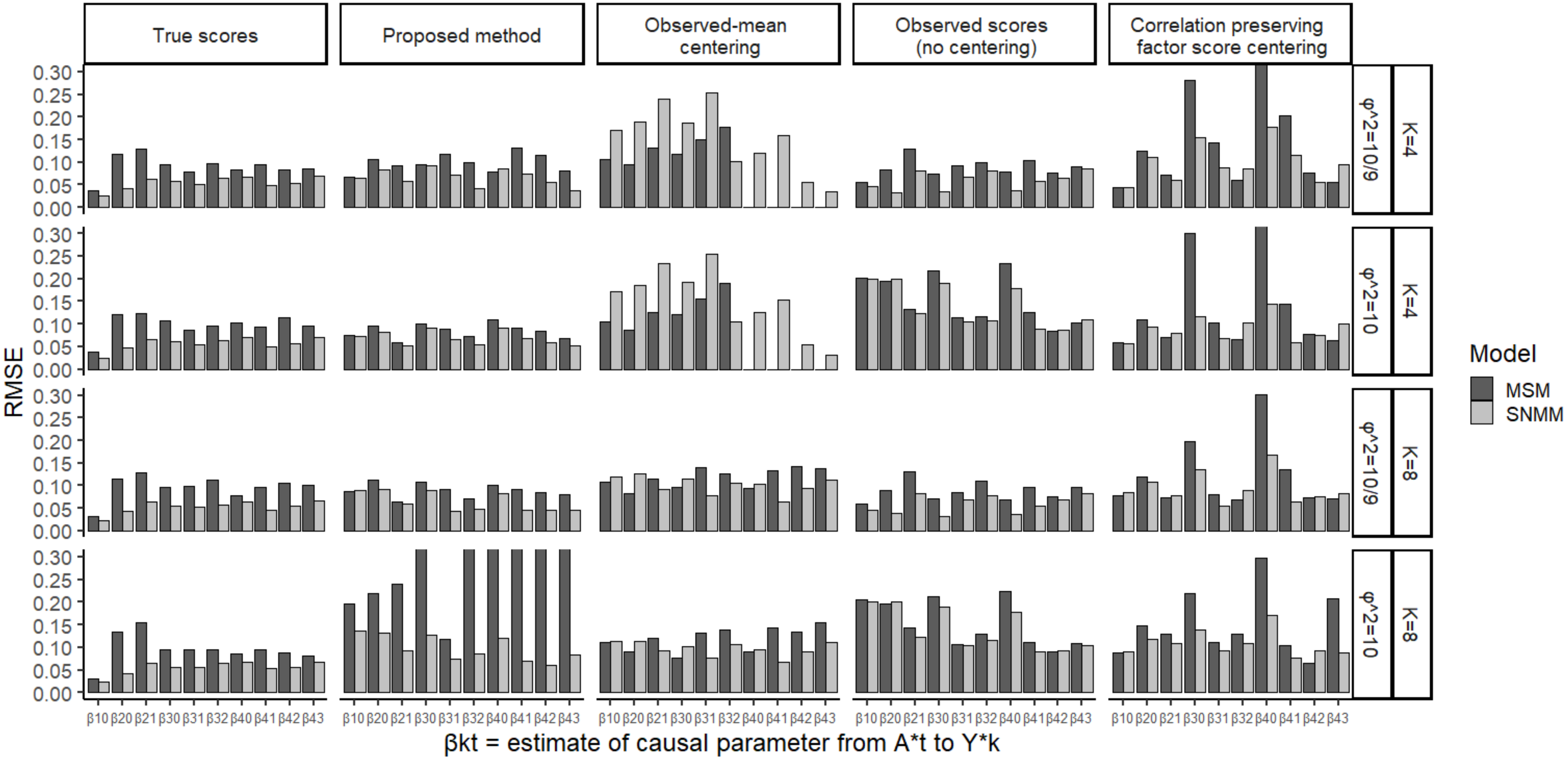

Figure S14. RMSEs of causal effect estimates ($N = 1{,}000$, quadratic effects of time-varying observed confounders are present in the treatment assignment model)

Note: Quadratic effects from time-varying observed confounders are included as $A^*_{ik} = 0.20Y^*_{ik} + 0.08Y^{*2}_{ik} + 0.40A^*_{i(k-1)} + 0.30L^*_{ik} + d^{(A)}_{ik}$.

Derivation of weight matrix W in Equations (17) and (19) of main manuscript

Let $X_i = (Y_i^t, A_i^t, L_i^t)^t$ be a $(3K+1) \times 1$ vector of observations. X_i is modeled using a stable trait factor (between-person difference) and a unique factor (within-person variability score) as

$$X_i = \mu + \Lambda I_i + X_i^*, \tag{A1}$$

where μ is a $(3K+1) \times 1$ mean vector, Λ is a $(3K+1) \times 3$ binary matrix (factor loading matrix) that connects the stable trait factors $I = (I_i^{(Y)}, I_i^{(A)}, I_i^{(L)})^t$ and the observation for each variable, and X_i^* is a $(3K+1) \times 1$ vector of within-person variability scores. Stable trait factors are uncorrelated with within-person variability scores: $cov(I_i, X_i^*) = 0.$ Unlike in the standard factor analysis model, the covariance structure (i.e., $E\left(\hat{X}_i^* \hat{X}_i^{t*}\right) = \Psi$) is not diagonal.

We are concerned with linear prediction of within-person variability scores using the $(3K+1) \times (3K+1)$ matrix W:

$$\hat{X}_i^* = W^t (X_i - \mu), \tag{A2}$$

which preserves the covariance structure of within-person variability scores:

$$E\left(\hat{X}_i^* \hat{X}_i^{t*}\right) = W^t E(X_i X_i^t) W = W^t \Sigma W = \Psi. \tag{A3}$$

Assume that Ψ and Σ are positive definite and are known because they are already estimated (as described in Section 4.1.1 of the main manuscript). Consider the following risk function defined as the trace of the usual mean squared error in within-person variability scores:

$$MSE\left(\hat{X}_i^*\right) = E\left[\left(\hat{X}_i^* - X_i^*\right)^t \left(\hat{X}_i^* - X_i^*\right)\right] = trE\left[\left(\hat{X}_i^* - X_i^*\right)\left(\hat{X}_i^* - X_i^*\right)^t\right]. \tag{A4}$$

From Equations (A1) and (A3), this risk function can be expressed as

$$MSE\left(\hat{X}_i^*\right) = trW^t\Sigma W + tr\Psi - 2tr\Psi W = 2tr\Psi - 2tr\Psi W. \quad \text{(A5)}$$

Therefore, minimizing Equation (A5) is mathematically equivalent to minimizing $tr\Psi W$. From Equation (A3), W can be expressed using a $(3K+1) \times (3K+1)$ orthogonal matrix Q as

$$W = \Sigma^{-1/2}Q\Psi^{1/2} \text{ for some } Q\text{: } (3K+1) \times (3K+1) \text{ such that } Q^tQ = I. \quad \text{(A6)}$$

From Equation (A6), $tr\Psi W$ can now be expressed as

$$tr\Psi W = tr\Psi\Sigma^{-1/2}Q\Psi^{1/2} = tr\Psi^{3/2}\Sigma^{-1/2}Q. \quad \text{(A7)}$$

Consider the singular value decomposition of $\Sigma^{-1/2}\Psi^{3/2}$ as

$$\Sigma^{-1/2}\Psi^{3/2} = UDV^t, \quad \text{(A8)}$$

where D is a diagonal matrix, and the matrices U, V satisfy $V^tV = VV^t = U^tU = UU^t = I$. From Equation (A8), Equation (A7) now becomes

$$tr\Psi^{3/2}\Sigma^{-1/2}Q = trVDU^tQ = trU^tQVD. \quad \text{(A9)}$$

Because of the relation $-1 \leq [U^tQV]_{kk} \leq 1$ $(k = 1, \cdots, 3K+1)$, Equation (A9) can be maximized (and Equation A5 can be minimized) when $Q = UV^t$. Therefore,

$$Q = UV^t = UDV^t(VD^2V^t)^{-1/2} = UDV^t(VDU^tUDV^t)^{-1/2} = \Sigma^{-1/2}\Psi^{3/2}(\Psi^{3/2}\Sigma^{-1}\Psi^{3/2})^{-1/2}. \quad \text{(A10)}$$

From Equations (A6) and (A10), W can now be expressed as

$$W = \Sigma^{-1}\Psi^{3/2}(\Psi^{3/2}\Sigma^{-1}\Psi^{3/2})^{-1/2}\Psi^{1/2}. \quad \text{(A11)}$$

Therefore, the best linear predictor for within-person variability scores can be obtained as

$$\hat{X}_i^* = W^t(X_i - \mu) = \Psi^{1/2}(\Psi^{3/2}\Sigma^{-1}\Psi^{3/2})^{-1/2}\Psi^{3/2}\Sigma^{-1}(X_i - \mu), \tag{A12}$$

which corresponds to Equations (17) and (19) in the main manuscript.

R Code for Simulations (*K*=4 condition)

```
SIMULATION<-function(TTT,REPEAT){
#Store the simulation results in POINTEST and SEEST
POINTEST<-matrix(rep(0,3*4*2*3*104),3*4*2*3,104);SEEST<-matrix(rep(0,3*4*2*3*104),3*4*2*3,104)

#Download required packages
library(lavaan);library(MASS); library(geepack);library(survey);library(ipw);library(reshape);library(dplyr)

#For loops (aaaa associates sample size N, bbbb associates stable trait variances TRAITVAR)
for(aaaa in 1:3){; for(bbbb in 1:3){;

#REPEAT=200 times in this simulation
COUNT<-0;while(COUNT<REPEAT){
N<-c(200,600,1000)[aaaa]; TRAITVAR<-c(10/9,30/7,10)[bbbb]

#Generate simulation data
#Generate stable trait factor scores
MV<-mvrnorm(N,c(0,0,0), (TRAITVAR*0.3)+(TRAITVAR*0.7)*diag(3))
IY<- MV[,1]-mean(MV[,1]); IA<- MV[,2]-mean(MV[,2]);IL<-MV[,3] -mean(MV[,3])
#Generate within-person variability scores
dMV<-mvrnorm(N,c(0,0,0), 3+7*diag(3)) ;dY0<- dMV[,1]-mean(dMV[,1]); dL0<- dMV[,2]-mean(dMV[,2])
dMV<-mvrnorm(N,c(0,0), 3+7*diag(2)) #variances and covariance are set to 10 and 3, respectively.
dY0<- dMV[,1]-mean(dMV[,1]); dL0<- dMV[,2]-mean(dMV[,2]) ;
dA0<-(3/13)*dY0+(3/13)*dL0+rnorm(N,0,sqrt(10-234/169))#this specification makes model-implied variance of dA0 and covariance between dA0 and dY0
(as well as covariance between dA0 and dL0) 10 and 3, respectively.
dA0<-dA0-mean(dA0)

dL0A0<-dL0*dA0; dY0A0<-dY0*dA0
dY1<-0.4*dY0+0.1*dL0+0.4*dA0+0.00*dL0A0+0.0*dY0A0+rnorm(N,0,sqrt(5));dY1<-dY1-mean(dY1)

dL1<-0.2*dY0+0.5*dL0+0.2*dA0+rnorm(N,0,sqrt(5)) ; dL1<-dL1-mean(dL1)
dA1<-0.3*dL1+0.2*dY1+0.4*dA0+rnorm(N,0,sqrt(5));dA1<-dA1-mean(dA1);dL1A1<-dL1*dA1;dY1A1<-dY1*dA1
dY2<-0.4*dY1+0.1*dL1+0.4*dA1+0.00*dL1A1+0.0*dY1A1+rnorm(N,0,sqrt(5));dY2<-dY2-mean(dY2)

dL2<-0.2*dY1+0.5*dL1+0.2*dA1+rnorm(N,0,sqrt(5)) ; dL2<-dL2-mean(dL2)
dA2<-0.3*dL2+0.2*dY2+0.4*dA1+rnorm(N,0,sqrt(5));dA2<-dA2-mean(dA2);dL2A2<-dL2*dA2;dY2A2<-dY2*dA2
dY3<-0.4*dY2+0.1*dL2+0.4*dA2+0.00*dL2A2+0.0*dY2A2+rnorm(N,0,sqrt(5));dY3<-dY3-mean(dY3)

dL3<-0.2*dY2+0.5*dL2+0.2*dA2+rnorm(N,0,sqrt(5)) ; dL3<-dL3-mean(dL3)
dA3<-0.3*dL3+0.2*dY3+0.4*dA2+rnorm(N,0,sqrt(5));dA3<-dA3-mean(dA3);dL3A3<-dL3*dA3;dY3A3<-dY3*dA3
dY4<-0.4*dY3+0.1*dL3+0.4*dA3+0.00*dL3A3+0.0*dY3A3+rnorm(N,0,sqrt(5));dY4<-dY4-mean(dY4)

#Generate observed scores and dataset
Y0<-dY0+IY; Y1<-dY1+IY; Y2<-dY2+IY; Y3<-dY3+IY; Y4<-dY4+IY;
L0<-dL0+IL; L0<-dL0+IL; L1<-dL1+IL; L2<-dL2+IL; L3<-dL3+IL
A0<-dA0+IA; A0<-dA0+IA; A1<-dA1+IA; A2<-dA2+IA; A3<-dA3+IA
YY0<-Y0; YY1<-Y1;YY2<-Y2;YY3<-Y3;YY4<-Y4; LL0<-L0; LL1<-L1;LL2<-L2;LL3<-L3; AA0<-A0; AA1<-A1;AA2<-A2;AA3<-A3;
```

#STEP1 Predict within-person variability scores

#Step 1.1 Estimate model parameters for a variable Y

```
#lavaan code
FAIY <- 'TraitY =~1*Y0+1*Y1 +1*Y2+1*Y3 +1*Y4
dY0=~1*Y0;dY1=~1*Y1; dY2=~1*Y2; dY3=~1*Y3; dY4=~1*Y4
dY1~beta1*dY0; dY2~beta2*dY1; dY3~beta3*dY2; dY4~beta4*dY3;
TraitY~~0*dY0;TraitY~~0*dY1; TraitY~~0*dY2; TraitY~~0*dY3; TraitY~~0*dY4;
TraitY~~vy*TraitY;vy>0
dY1~~omega*dY1; dY2~~omega2*dY2;dY3~~omega3*dY3;dY4~~omega4*dY4
Y0~~ 0 *Y0;Y1~~0*Y1;Y2~~ 0 *Y2;Y3~~ 0 *Y3;Y4~~ 0*Y4; omega>0;omega2>0 ; omega3>0;omega4>0;
'
#Model fitting
fit <- cfa(FAIY, data=cbind(Y0,Y1,Y2,Y3,Y4));
PhiY<- parameterEstimates(fit)[20,5] #Trait factor variance estimate of Y
LambdaY<-rep(1,5);PsiY<- fitted(fit)$cov-PhiY;SigmaY<- cov(cbind(Y0,Y1,Y2,Y3,Y4))

FYres<-rep(0,N); #Store correlation-preserving predictor for Y
for(i in 1:N){
YY<-c(Y0[i],Y1[i], Y2[i], Y3[i], Y4[i])
FYres[i]<-PhiY^(1/2)* (1/sqrt(t(LambdaY)%*%solve(SigmaY)%*%LambdaY))%*%t(LambdaY)%*% solve(SigmaY)%*%YY
}
FYres<-FYres-mean(FYres)
#Evalute the presence of improper solutions
IMPRO1<-sum(c(sign(parameterEstimates(fit)[c(20:24,30),5])) )+ ifelse(is.na(FYres[1]),0,1) #7
```

```
#Step 1.1 Estimate model parameters for a variable L
#lavaan code
FAIL <- 'TraitL =~1*L0 +1*L1 +1*L2+1*L3;
dL0=~1*L0; dL1=~1*L1; dL2=~1*L2; dL3=~1*L3
dL1~beta*dL0; dL2~beta2*dL1; dL3~beta3*dL2;
TraitL~~0*dL0; TraitL~~0*dL1; TraitL~~0*dL2; TraitL~~0*dL3;
TraitL~~vy*TraitL;
L0~~ 0*L0; L1~~ 0*L1;L2~~ 0*L2;L3~~ 0*L3;omega>0
dL1~~omega*dL1; dL2~~omega2*dL2; dL3~~omega3*dL3;
;omega>0;omega2>0;omega3>0'
#Model fitting
fit <- cfa(FAIL, data=cbind(L0,L1,L2,L3))
PhiL<- parameterEstimates(fit)[16,5] #Trait factor variance estimate of L
LambdaL<-rep(1,4);PsiL<- fitted(fit)$cov-PhiL;SigmaL<-cov(cbind(L0,L1,L2,L3))
FLres<-rep(0,N) #Store correlation-preserving predictor for Y
for(i in 1:N){
LL<-c(L0[i], L1[i], L2[i], L3[i])
FLres[i]<-PhiL^(1/2)* (1/sqrt(t(LambdaL)%*%solve(SigmaL)%*%LambdaL))%*%t(LambdaL)%*% solve(SigmaL)%*%LL
}
FLres<-FLres-mean(FLres);
#Evaluate the presence of improper solutions
IMPRO2<-sum(c(sign(parameterEstimates(fit)[c(16,21:24),5])) ) + ifelse(is.na(FLres[1]),0,1) #6

#Step 1.1 Estimate model parameters for a variable A
#lavaan code
FAIA <- 'TraitA =~1*A0 +1*A1 +1*A2+1*A3
dA0=~1*A0; dA1=~1*A1; dA2=~1*A2; dA3=~1*A3;
dA1~beta*dA0; dA2~beta2*dA1; dA3~beta3*dA2
TraitA~~0*dA0; TraitA~~0*dA1; TraitA~~0*dA2; TraitA~~0*dA3;
TraitA~~vy*TraitA;
A0~~0*A0; A1~~ 0*A1;A2~~ 0*A2;A3~~ 0*A3
dA1~~omega*dA1; dA2~~omega2*dA2; dA3~~omega3*dA3;
;omega>0;omega2>0;omega3>0 '
#Model fitting
fit <- cfa(FAIA, data=cbind(A0,A1,A2,A3))
PhiA<- parameterEstimates(fit)[16,5] #Trait factor variance estimate of A
LambdaA<-rep(1,4);PsiA<- fitted(fit)$cov-PhiA;SigmaA<- cov(cbind(A0,A1,A2,A3))
FAres<-rep(0,N) #Store correlation-preserving predictor for A
for(i in 1:N){
AA<-c(A0[i], A1[i], A2[i], A3[i])
FAres[i]<-PhiA^(1/2)* (1/sqrt(t(LambdaA)%*%solve(SigmaA)%*%LambdaA))%*%t(LambdaA)%*% solve(SigmaA)%*%AA
}
FAres<-FAres-mean(FAres)
#Evaluate the presence of improper solutions
IMPRO3<-sum(c(sign(parameterEstimates(fit)[c(16,21:24),5])) ) + ifelse(is.na(FAres[1]),0,1) #6

#Continue if no improper solutions were found
if(IMPRO1+ IMPRO2+ IMPRO3>18){

#Step 1.2 Predict within-person variability scores
SigmaX<-var(cbind(Y0,Y1,Y2,Y3,Y4,A0,A1,A2,A3,L0,L1,L2,L3))
Imat<-matrix(rep(0,13*13),13,13) #Store trait (co)variances matrix
Imat[1:5,1:5]<-PhiY; Imat[6:9,6:9]<-PhiA; Imat[10:13,10:13]<-PhiL;
Imat[1:5,6:9]<-cov(FYres,FAres);Imat[6:9,1:5]<-cov(FYres,FAres);Imat[1:5,10:13]<-cov(FYres,FLres)
Imat[10:13,1:5]<-cov(FYres,FLres);Imat[6:9,10:13]<-cov(FAres,FLres);Imat[10:13,6:9]<-cov(FAres,FLres)
PsiX<-SigmaX-Imat

#Singular value decomposition
  UPsiX <- svd(PsiX)$u; VPsiX <- svd(PsiX)$v ;DPsiX <- diag(sqrt(svd(PsiX)$d))
  PsiXhalf<- UPsiX %*% DPsiX %*% t(VPsiX)
  USigmaX <- svd(SigmaX)$u; VSigmaX <- svd(SigmaX)$v; DSigmaX <- diag(sqrt(svd(SigmaX)$d))
  SigmaXhalf<- USigmaX %*% DSigmaX %*% t(VSigmaX)

                SIGX<-PsiX%*%PsiXhalf%*%solve(SigmaX)%*%PsiX%*%PsiXhalf

  USIGX <- svd(SIGX)$u;VSIGX <- svd(SIGX)$v; DSIGX <- diag(sqrt(svd(SIGX)$d))
  SIGXhalf<- USIGX %*% DSIGX %*% t(VSIGX)

#Calculate weights
    WWX<-PsiXhalf%*% solve(SIGXhalf)%*% PsiX%*%PsiXhalf%*%solve(SigmaX)

#Calculate within-person variability scores
FYwith2<-matrix(rep(0,N*13),N,13);for(i in 1:N){
XX<-c(Y0[i],Y1[i], Y2[i], Y3[i], Y4[i], A0[i],A1[i], A2[i], A3[i], L0[i],L1[i], L2[i], L3[i])
FYwith2[i,]<-WWX%*%XX
}
```

```
#Step 2 Estimate Causal Parameters by SNMM

#True score condition
Y0<-   dY0
Y1<-   dY1
Y2<-   dY2
Y3<-   dY3
Y4<-     dY4
L0<-dL0; L1<-dL1; L2<-dL2; L3<-dL3; A0<-dA0; A1<-dA1; A2<-dA2; A3<-dA3

Y3sq<-Y3^2;L3sq<-L3^2;A2sq<-A2^2; Y2sq<-Y2^2;L2sq<-L2^2;A1sq<-A1^2; Y1sq<-Y1^2;L1sq<-L1^2;A0sq<-A0^2; Y0sq<-Y0^2;L0sq<-L0^2;
Y3L3<-Y3*L3; Y2L2<-Y2*L2; Y1L1<-Y1*L1; Y0L0<-Y0*L0;
Y3A3<-Y3*A3; Y2A2<-Y2*A2; Y1A1<-Y1*A1; Y0A0<-Y0*A0;
L3A3<-L3*A3; L2A2<-L2*A2; L1A1<-L1*A1; L0A0<-L0*A0;
#Estimate P(Y|A,L)
PredY43<-lm(Y4~ Y3+L3+A2+ Y3sq+L3sq +A2sq+Y3L3)
PredY42<-lm(Y4~ Y2+L2+A1+ Y2sq+L2sq +A1sq+Y2L2)
PredY41<-lm(Y4~ Y1+L1+A0+ Y1sq+L1sq +A0sq+Y1L1)
PredY40<-lm(Y4~ Y0+L0+Y0sq+L0sq+Y0L0)
PredY32<-lm(Y3~ Y2+L2+A1+ Y2sq+L2sq +A1sq+Y2L2)
PredY31<-lm(Y3~ Y1+L1+A0+ Y1sq+L1sq +A0sq+Y1L1)
PredY30<-lm(Y3~ Y0+L0+Y0sq+L0sq+Y0L0)
PredY21<-lm(Y2~ Y1+L1+A0+ Y1sq+L1sq +A0sq+Y1L1)
PredY20<-lm(Y2~ Y0+L0+Y0sq+L0sq+Y0L0)
PredY10<-lm(Y1~ Y0+L0+Y0sq+L0sq +Y0L0)
#Estimate P(A|A,L)
PredA33<-lm(A3~ Y3+L3+A2+ L3sq +A2sq+Y3L3)
PredA32<-lm(A3~ Y2+L2+A1+ L2sq +A1sq+Y2L2)
PredA31<-lm(A3~ Y1+L1+A0+ L1sq +A0sq+Y1L1)
PredA30<-lm(A3~ Y0+L0+L0sq+Y0L0)
PredA22<-lm(A2~ Y2+L2+A1+L2sq +A1sq+Y2L2)
PredA21<-lm(A2~ Y1+L1+A0+L1sq +A0sq+Y1L1)
PredA20<-lm(A2~ Y0+L0+L0sq+Y0L0)
PredA11<-lm(A1~ Y1+L1+A0+L1sq +A0sq+Y1L1)
PredA10<-lm(A1~ Y0+L0+L0sq+Y0L0)
PredA00<-lm(A0~ Y0+L0+L0sq+Y0L0)

DATA3<-data.frame(cbind(Y3,L3,A2, Y3sq,L3sq ,A2sq,Y3L3));DATA2<-data.frame(cbind(Y2,L2,A1, Y2sq,L2sq ,A1sq,Y2L2))
DATA1<-data.frame(cbind(Y1,L1,A0, Y1sq,L1sq ,A0sq,Y1L1));DATA0<-data.frame(cbind(Y0,L0, Y0sq,L0sq ,Y0L0))
#Set initial values
beta43<-0.5; beta42<-0.3; beta41<-0.5; beta40<-0.3; beta32<-0.5; beta31<-0.5; beta30<-0.5; beta21<-0.3; beta20<-0.5; beta10<-0.5

V1<-var(Y4-predict(PredY43,DATA3)-(beta43*(A3-predict(PredA33,DATA3))))
V2<-var(Y4-predict(PredY42,DATA2)-(beta43*(A3-predict(PredA32,DATA2))+beta42*(A2-predict(PredA22,DATA2))))
V3<-var(Y4-predict(PredY41,DATA1)-(beta43*(A3-predict(PredA31,DATA1))+beta42*(A2-predict(PredA21,DATA1))+beta41*(A1-
predict(PredA11,DATA1))))
V4<-var(Y4-predict(PredY40,DATA0)-(beta43*(A3-predict(PredA30,DATA0))+beta42*(A2-predict(PredA20,DATA0))+beta41*(A1-
predict(PredA10,DATA0))+beta40*(A0-predict(PredA00,DATA0))))
V5<-var(Y3-predict(PredY32,DATA2)-(beta32*(A2-predict(PredA22,DATA2))))
V6<-var(Y3-predict(PredY31,DATA1)-(beta32*(A2-predict(PredA21,DATA1))+beta31*(A1-predict(PredA11,DATA1))))
V7<-var(Y3-predict(PredY30,DATA0)-(beta32*(A2-predict(PredA20,DATA0))+beta31*(A1-predict(PredA10,DATA0))+beta30*(A0-
predict(PredA00,DATA0))))
V8<-var(Y2-predict(PredY21,DATA1)-(beta21*(A1-predict(PredA11,DATA1))))
V9<-var(Y2-predict(PredY20,DATA0)-(beta21*(A1-predict(PredA10,DATA0))+beta20*(A0-predict(PredA00,DATA0))))
V10<-var(Y1-predict(PredY10,DATA0)-(beta10*(A0-predict(PredA00,DATA0))))
#Solve estimating equations via the Newton-Raphson Method
CHECK<-0; ITER1<-0; while(CHECK<1 && ITER1<200){
#First-order differentiation
dI<-rep(0,10); ITER1<-ITER1+1
dI[1]<-sum(
(A3-predict(PredA33,DATA3)) *(1/V1)*(Y4-predict(PredY43,DATA3)-(beta43*(A3-predict(PredA33,DATA3))))
)
dI[2]<-sum(
(A2-predict(PredA22,DATA2))*(1/V2)*(Y4-predict(PredY42,DATA2)-(beta43*(A3-predict(PredA32,DATA2))+beta42*(A2-predict(PredA22,DATA2))))
)
dI[3]<-sum(
(A1-predict(PredA11,DATA1))*(1/V3)*(Y4-predict(PredY41,DATA1)-(beta43*(A3-predict(PredA31,DATA1))+beta42*(A2-
predict(PredA21,DATA1))+beta41*(A1-predict(PredA11,DATA1))))
)
dI[4]<-sum(
(A0-predict(PredA00,DATA0))*(1/V4)*(Y4-predict(PredY40,DATA0)-(beta43*(A3-predict(PredA30,DATA0))+beta42*(A2-
predict(PredA20,DATA0))+beta41*(A1-predict(PredA10,DATA0))+beta40*(A0-predict(PredA00,DATA0))))
)

dI[5]<-sum(
(A2-predict(PredA22,DATA2)) *(1/V5)*(Y3-predict(PredY32,DATA2)-(beta32*(A2-predict(PredA22,DATA2))))
```

```
)
dI[6]<-sum(
(A1-predict(PredA11,DATA1))*(1/V6)*(Y3-predict(PredY31,DATA1)-(beta32*(A2-predict(PredA21,DATA1))+beta31*(A1-predict(PredA11,DATA1))))
)
dI[7]<-sum(
(A0-predict(PredA00,DATA0))*(1/V7)*(Y3-predict(PredY30,DATA0)-(beta32*(A2-predict(PredA20,DATA0))+beta31*(A1-
predict(PredA10,DATA0))+beta30*(A0-predict(PredA00,DATA0))))
)
dI[8]<-sum(
(A1-predict(PredA11,DATA1))*(1/V8)*(Y2-predict(PredY21,DATA1)-(beta21*(A1-predict(PredA11,DATA1))))
)
dI[9]<-sum(
(A0-predict(PredA00,DATA0))*(1/V9)*(Y2-predict(PredY20,DATA0)-(beta21*(A1-predict(PredA10,DATA0))+beta20*(A0-predict(PredA00,DATA0))))
)
dI[10]<-sum(
(A0-predict(PredA00,DATA0)) *(1/V10)*( Y1-predict(PredY10,DATA0)-(beta10*(A0-predict(PredA00,DATA0))))
)

#Second-order differentiation
dII<-rep(0,5)
dII[1]<-sum(
(A3-predict(PredA33,DATA3)) *(1/V1)* (A3-predict(PredA33,DATA3))
)
dII[2]<-sum(
(A2-predict(PredA22,DATA2)) *(1/V2)* (A2-predict(PredA22,DATA2))
)
dII[3]<-sum(
(A1-predict(PredA11,DATA1)) *(1/V3)* (A1-predict(PredA11,DATA1))
)
dII[4]<-sum(
(A0-predict(PredA00,DATA0)) *(1/V4)* (A0-predict(PredA00,DATA0))
)
dII[5]<-sum(
(A2-predict(PredA22,DATA2)) *(1/V5)* (A2-predict(PredA22,DATA2))
)
dII[6]<-sum(
(A1-predict(PredA11,DATA1)) *(1/V6)* (A1-predict(PredA11,DATA1))
)
dII[7]<-sum(
(A0-predict(PredA00,DATA0)) *(1/V7)* (A0-predict(PredA00,DATA0))
)
dII[8]<-sum(
(A1-predict(PredA11,DATA1)) *(1/V8)* (A1-predict(PredA11,DATA1))
)
dII[9]<-sum(
(A0-predict(PredA00,DATA0)) *(1/V9)* (A0-predict(PredA00,DATA0))
)
dII[10]<-sum(
(A0-predict(PredA00,DATA0)) *(1/V10)* (A0-predict(PredA00,DATA0))
)

#Update parameter values
PSI<- c(beta43,beta42,beta41,beta40,beta32,beta31,beta30,beta21,beta20,beta10);NEW<-PSI+diag(1/dII)%*%dI
#Check the convergence criterion
CHECK<-ifelse(max(abs(PSI-NEW))<0.0001,1,0)
beta43<-NEW[1] ; beta42<-NEW[2]; beta41<-NEW[3] ; beta40<-NEW[4] ; beta32<-NEW[5]; beta31<-NEW[6]
beta30<-NEW[7] ; beta21<-NEW[8]; beta20<-NEW[9] ; beta10<-NEW[10]
V1<-var(Y4-predict(PredY43,DATA3)-(beta43*(A3-predict(PredA33,DATA3))))
V2<-var(Y4-predict(PredY42,DATA2)-(beta43*(A3-predict(PredA32,DATA2))+beta42*(A2-predict(PredA22,DATA2))))
V3<-var(Y4-predict(PredY41,DATA1)-(beta43*(A3-predict(PredA31,DATA1))+beta42*(A2-predict(PredA21,DATA1))+beta41*(A1-
predict(PredA11,DATA1))))
V4<-var(Y4-predict(PredY40,DATA0)-(beta43*(A3-predict(PredA30,DATA0))+beta42*(A2-predict(PredA20,DATA0))+beta41*(A1-
predict(PredA10,DATA0))+beta40*(A0-predict(PredA00,DATA0))))
V5<-var(Y3-predict(PredY32,DATA2)-(beta32*(A2-predict(PredA22,DATA2))))
V6<-var(Y3-predict(PredY31,DATA1)-(beta32*(A2-predict(PredA21,DATA1))+beta31*(A1-predict(PredA11,DATA1))))
V7<-var(Y3-predict(PredY30,DATA0)-(beta32*(A2-predict(PredA20,DATA0))+beta31*(A1-predict(PredA10,DATA0))+beta30*(A0-
predict(PredA00,DATA0))))
V8<-var(Y2-predict(PredY21,DATA1)-(beta21*(A1-predict(PredA11,DATA1))))
V9<-var(Y2-predict(PredY20,DATA0)-(beta21*(A1-predict(PredA10,DATA0))+beta20*(A0-predict(PredA00,DATA0))))
V10<-var(Y1-predict(PredY10,DATA0)-(beta10*(A0-predict(PredA00,DATA0))))
};
#Store estimates and SEs
PSI1<-PSI;SE1<-sqrt(1/dII)
```

```
#Observed score (no centering) condition
Y0<-   YY0-mean(YY0)
Y1<-   YY1-mean(YY1)
Y2<-   YY2-mean(YY2)
Y3<-   YY3-mean(YY3)
Y4<-   YY4-mean(YY4)
L0<-LL0-mean(LL0);  L1<-LL1-mean(LL1);  L2<-LL2-mean(LL2);  L3<-LL3-mean(LL3);  A0<-AA0-mean(AA0);  A1<-AA1-mean(AA1);  A2<-AA2-
mean(AA2); A3<-AA3-mean(AA3)


Y3sq<-Y3^2;L3sq<-L3^2;A2sq<-A2^2; Y2sq<-Y2^2;L2sq<-L2^2;A1sq<-A1^2; Y1sq<-Y1^2;L1sq<-L1^2;A0sq<-A0^2; Y0sq<-Y0^2;L0sq<-L0^2;
Y3L3<-Y3*L3; Y2L2<-Y2*L2; Y1L1<-Y1*L1; Y0L0<-Y0*L0;
Y3A3<-Y3*A3; Y2A2<-Y2*A2; Y1A1<-Y1*A1; Y0A0<-Y0*A0;
L3A3<-L3*A3; L2A2<-L2*A2; L1A1<-L1*A1; L0A0<-L0*A0;
#Estimate P(Y|A,L)
PredY43<-lm(Y4~ Y3+L3+A2+ Y3sq+L3sq +A2sq+Y3L3)
PredY42<-lm(Y4~ Y2+L2+A1+ Y2sq+L2sq +A1sq+Y2L2)
PredY41<-lm(Y4~ Y1+L1+A0+ Y1sq+L1sq +A0sq+Y1L1)
PredY40<-lm(Y4~ Y0+L0+Y0sq+L0sq+Y0L0)
PredY32<-lm(Y3~ Y2+L2+A1+ Y2sq+L2sq +A1sq+Y2L2)
PredY31<-lm(Y3~ Y1+L1+A0+ Y1sq+L1sq +A0sq+Y1L1)
PredY30<-lm(Y3~ Y0+L0+Y0sq+L0sq+Y0L0)
PredY21<-lm(Y2~ Y1+L1+A0+ Y1sq+L1sq +A0sq+Y1L1)
PredY20<-lm(Y2~ Y0+L0+Y0sq+L0sq+Y0L0)
PredY10<-lm(Y1~ Y0+L0+Y0sq+L0sq +Y0L0)
#Estimate P(A|A,L)
PredA33<-lm(A3~ Y3+L3+A2+ L3sq +A2sq+Y3L3)
PredA32<-lm(A3~ Y2+L2+A1+ L2sq +A1sq+Y2L2)
PredA31<-lm(A3~ Y1+L1+A0+ L1sq +A0sq+Y1L1)
PredA30<-lm(A3~ Y0+L0+L0sq+Y0L0)
PredA22<-lm(A2~ Y2+L2+A1+L2sq +A1sq+Y2L2)
PredA21<-lm(A2~ Y1+L1+A0+L1sq +A0sq+Y1L1)
PredA20<-lm(A2~ Y0+L0+L0sq+Y0L0)
PredA11<-lm(A1~ Y1+L1+A0+L1sq +A0sq+Y1L1)
PredA10<-lm(A1~ Y0+L0+L0sq+Y0L0)
PredA00<-lm(A0~ Y0+L0+L0sq+Y0L0)

DATA3<-data.frame(cbind(Y3,L3,A2, Y3sq,L3sq ,A2sq,Y3L3));DATA2<-data.frame(cbind(Y2,L2,A1, Y2sq,L2sq ,A1sq,Y2L2))
DATA1<-data.frame(cbind(Y1,L1,A0, Y1sq,L1sq ,A0sq,Y1L1));DATA0<-data.frame(cbind(Y0,L0, Y0sq,L0sq ,Y0L0))
#Set initial values
beta43<-0.5; beta42<-0.3; beta41<-0.5; beta40<-0.3; beta32<-0.5; beta31<-0.5; beta30<-0.5; beta21<-0.3; beta20<-0.5; beta10<-0.5

V1<-var(Y4-predict(PredY43,DATA3)-(beta43*(A3-predict(PredA33,DATA3))))
V2<-var(Y4-predict(PredY42,DATA2)-(beta43*(A3-predict(PredA32,DATA2))+beta42*(A2-predict(PredA22,DATA2))))
V3<-var(Y4-predict(PredY41,DATA1)-(beta43*(A3-predict(PredA31,DATA1))+beta42*(A2-predict(PredA21,DATA1))+beta41*(A1-
predict(PredA11,DATA1))))
V4<-var(Y4-predict(PredY40,DATA0)-(beta43*(A3-predict(PredA30,DATA0))+beta42*(A2-predict(PredA20,DATA0))+beta41*(A1-
predict(PredA10,DATA0))+beta40*(A0-predict(PredA00,DATA0))))
V5<-var(Y3-predict(PredY32,DATA2)-(beta32*(A2-predict(PredA22,DATA2))))
V6<-var(Y3-predict(PredY31,DATA1)-(beta32*(A2-predict(PredA21,DATA1))+beta31*(A1-predict(PredA11,DATA1))))
V7<-var(Y3-predict(PredY30,DATA0)-(beta32*(A2-predict(PredA20,DATA0))+beta31*(A1-predict(PredA10,DATA0))+beta30*(A0-
predict(PredA00,DATA0))))
V8<-var(Y2-predict(PredY21,DATA1)-(beta21*(A1-predict(PredA11,DATA1))))
V9<-var(Y2-predict(PredY20,DATA0)-(beta21*(A1-predict(PredA10,DATA0))+beta20*(A0-predict(PredA00,DATA0))))
V10<-var(Y1-predict(PredY10,DATA0)-(beta10*(A0-predict(PredA00,DATA0))))
#Solve estimating equations via the Newton-Raphson Method
CHECK<-0; ITER2<-0; while(CHECK<1 && ITER2<200){
#First-order differentiation
dI<-rep(0,10); ITER2<-ITER2+1
dI[1]<-sum(
(A3-predict(PredA33,DATA3)) *(1/V1)*(Y4-predict(PredY43,DATA3)-(beta43*(A3-predict(PredA33,DATA3))))
)
dI[2]<-sum(
(A2-predict(PredA22,DATA2))*(1/V2)*(Y4-predict(PredY42,DATA2)-(beta43*(A3-predict(PredA32,DATA2))+beta42*(A2-predict(PredA22,DATA2))))
)
dI[3]<-sum(
(A1-predict(PredA11,DATA1))*(1/V3)*(Y4-predict(PredY41,DATA1)-(beta43*(A3-predict(PredA31,DATA1))+beta42*(A2-
predict(PredA21,DATA1))+beta41*(A1-predict(PredA11,DATA1))))
)
dI[4]<-sum(
(A0-predict(PredA00,DATA0))*(1/V4)*(Y4-predict(PredY40,DATA0)-(beta43*(A3-predict(PredA30,DATA0))+beta42*(A2-
predict(PredA20,DATA0))+beta41*(A1-predict(PredA10,DATA0))+beta40*(A0-predict(PredA00,DATA0))))
)

dI[5]<-sum(
(A2-predict(PredA22,DATA2)) *(1/V5)*(Y3-predict(PredY32,DATA2)-(beta32*(A2-predict(PredA22,DATA2))))
```

```
)
dI[6]<-sum(
(A1-predict(PredA11,DATA1))*(1/V6)*(Y3-predict(PredY31,DATA1)-(beta32*(A2-predict(PredA21,DATA1))+beta31*(A1-predict(PredA11,DATA1))))
)
dI[7]<-sum(
(A0-predict(PredA00,DATA0))*(1/V7)*(Y3-predict(PredY30,DATA0)-(beta32*(A2-predict(PredA20,DATA0))+beta31*(A1-
predict(PredA10,DATA0))+beta30*(A0-predict(PredA00,DATA0))))
)
dI[8]<-sum(
(A1-predict(PredA11,DATA1))*(1/V8)*(Y2-predict(PredY21,DATA1)-(beta21*(A1-predict(PredA11,DATA1))))
)
dI[9]<-sum(
(A0-predict(PredA00,DATA0))*(1/V9)*(Y2-predict(PredY20,DATA0)-(beta21*(A1-predict(PredA10,DATA0))+beta20*(A0-predict(PredA00,DATA0))))
)
dI[10]<-sum(
(A0-predict(PredA00,DATA0)) *(1/V10)*( Y1-predict(PredY10,DATA0)-(beta10*(A0-predict(PredA00,DATA0))))
)

#Second-order differentiation
dII<-rep(0,5)
dII[1]<-sum(
(A3-predict(PredA33,DATA3)) *(1/V1)* (A3-predict(PredA33,DATA3))
)
dII[2]<-sum(
(A2-predict(PredA22,DATA2)) *(1/V2)* (A2-predict(PredA22,DATA2))
)
dII[3]<-sum(
(A1-predict(PredA11,DATA1)) *(1/V3)* (A1-predict(PredA11,DATA1))
)
dII[4]<-sum(
(A0-predict(PredA00,DATA0)) *(1/V4)* (A0-predict(PredA00,DATA0))
)
dII[5]<-sum(
(A2-predict(PredA22,DATA2)) *(1/V5)* (A2-predict(PredA22,DATA2))
)
dII[6]<-sum(
(A1-predict(PredA11,DATA1)) *(1/V6)* (A1-predict(PredA11,DATA1))
)
dII[7]<-sum(
(A0-predict(PredA00,DATA0)) *(1/V7)* (A0-predict(PredA00,DATA0))
)
dII[8]<-sum(
(A1-predict(PredA11,DATA1)) *(1/V8)* (A1-predict(PredA11,DATA1))
)
dII[9]<-sum(
(A0-predict(PredA00,DATA0)) *(1/V9)* (A0-predict(PredA00,DATA0))
)
dII[10]<-sum(
(A0-predict(PredA00,DATA0)) *(1/V10)* (A0-predict(PredA00,DATA0))
)

#Update parameter values
PSI<- c(beta43,beta42,beta41,beta40,beta32,beta31,beta30,beta21,beta20,beta10);NEW<-PSI+diag(1/dII)%*%dI
#Check the convergence criterion
CHECK<-ifelse(max(abs(PSI-NEW))<0.0001,1,0)
beta43<-NEW[1] ; beta42<-NEW[2]; beta41<-NEW[3] ; beta40<-NEW[4] ; beta32<-NEW[5]; beta31<-NEW[6]
beta30<-NEW[7] ; beta21<-NEW[8]; beta20<-NEW[9] ; beta10<-NEW[10]
V1<-var(Y4-predict(PredY43,DATA3)-(beta43*(A3-predict(PredA33,DATA3))))
V2<-var(Y4-predict(PredY42,DATA2)-(beta43*(A3-predict(PredA32,DATA2))+beta42*(A2-predict(PredA22,DATA2))))
V3<-var(Y4-predict(PredY41,DATA1)-(beta43*(A3-predict(PredA31,DATA1))+beta42*(A2-predict(PredA21,DATA1))+beta41*(A1-
predict(PredA11,DATA1))))
V4<-var(Y4-predict(PredY40,DATA0)-(beta43*(A3-predict(PredA30,DATA0))+beta42*(A2-predict(PredA20,DATA0))+beta41*(A1-
predict(PredA10,DATA0))+beta40*(A0-predict(PredA00,DATA0))))
V5<-var(Y3-predict(PredY32,DATA2)-(beta32*(A2-predict(PredA22,DATA2))))
V6<-var(Y3-predict(PredY31,DATA1)-(beta32*(A2-predict(PredA21,DATA1))+beta31*(A1-predict(PredA11,DATA1))))
V7<-var(Y3-predict(PredY30,DATA0)-(beta32*(A2-predict(PredA20,DATA0))+beta31*(A1-predict(PredA10,DATA0))+beta30*(A0-
predict(PredA00,DATA0))))
V8<-var(Y2-predict(PredY21,DATA1)-(beta21*(A1-predict(PredA11,DATA1))))
V9<-var(Y2-predict(PredY20,DATA0)-(beta21*(A1-predict(PredA10,DATA0))+beta20*(A0-predict(PredA00,DATA0))))
V10<-var(Y1-predict(PredY10,DATA0)-(beta10*(A0-predict(PredA00,DATA0))))
};
#Store estimates and SEs
PSI2<-PSI;SE2<-sqrt(1/dII)
```

```
#Observed-mean centering condition
MY<-rowMeans(cbind(YY0,YY1,YY2,YY3)); ML<-rowMeans(cbind(LL0,LL1,LL2,LL3)); MA<-rowMeans(cbind(AA0,AA1,AA2,AA3))
Y0<-     (YY0-mean(YY0)-MY)
Y1<-     (YY1-mean(YY1)-MY)
Y2<-     (YY2-mean(YY2)-MY)
Y3<-     (YY3-mean(YY3)-MY)
Y4<-     (YY4-mean(YY4)-MY)
L0<-LL0-mean(LL0)-ML; L1<-LL1-mean(LL1)-ML; L2<-LL2-mean(LL2)-ML; L3<-LL3-mean(LL3)-ML;
A0<-AA0-mean(AA0)-MA; A1<-AA1-mean(AA1)-MA; A2<-AA2-mean(AA2)-MA; A3<-AA3-mean(AA3)-MA

Y3sq<-Y3^2;L3sq<-L3^2;A2sq<-A2^2; Y2sq<-Y2^2;L2sq<-L2^2;A1sq<-A1^2; Y1sq<-Y1^2;L1sq<-L1^2;A0sq<-A0^2; Y0sq<-Y0^2;L0sq<-L0^2;
Y3L3<-Y3*L3; Y2L2<-Y2*L2; Y1L1<-Y1*L1; Y0L0<-Y0*L0;
Y3A3<-Y3*A3; Y2A2<-Y2*A2; Y1A1<-Y1*A1; Y0A0<-Y0*A0;
L3A3<-L3*A3; L2A2<-L2*A2; L1A1<-L1*A1; L0A0<-L0*A0;
#Estimate P(Y|A,L)
PredY43<-lm(Y4~ Y3+L3+A2+ Y3sq+L3sq +A2sq+Y3L3)
PredY42<-lm(Y4~ Y2+L2+A1+ Y2sq+L2sq +A1sq+Y2L2)
PredY41<-lm(Y4~ Y1+L1+A0+ Y1sq+L1sq +A0sq+Y1L1)
PredY40<-lm(Y4~ Y0+L0+Y0sq+L0sq+Y0L0)
PredY32<-lm(Y3~ Y2+L2+A1+ Y2sq+L2sq +A1sq+Y2L2)
PredY31<-lm(Y3~ Y1+L1+A0+ Y1sq+L1sq +A0sq+Y1L1)
PredY30<-lm(Y3~ Y0+L0+Y0sq+L0sq+Y0L0)
PredY21<-lm(Y2~ Y1+L1+A0+ Y1sq+L1sq +A0sq+Y1L1)
PredY20<-lm(Y2~ Y0+L0+Y0sq+L0sq+Y0L0)
PredY10<-lm(Y1~ Y0+L0+Y0sq+L0sq +Y0L0)
#Estimate P(A|A,L)
PredA33<-lm(A3~ Y3+L3+A2+ L3sq +A2sq+Y3L3)
PredA32<-lm(A3~ Y2+L2+A1+ L2sq +A1sq+Y2L2)
PredA31<-lm(A3~ Y1+L1+A0+ L1sq +A0sq+Y1L1)
PredA30<-lm(A3~ Y0+L0+L0sq+Y0L0)
PredA22<-lm(A2~ Y2+L2+A1+L2sq +A1sq+Y2L2)
PredA21<-lm(A2~ Y1+L1+A0+L1sq +A0sq+Y1L1)
PredA20<-lm(A2~ Y0+L0+L0sq+Y0L0)
PredA11<-lm(A1~ Y1+L1+A0+L1sq +A0sq+Y1L1)
PredA10<-lm(A1~ Y0+L0+L0sq+Y0L0)
PredA00<-lm(A0~ Y0+L0+L0sq+Y0L0)

DATA3<-data.frame(cbind(Y3,L3,A2, Y3sq,L3sq ,A2sq,Y3L3));DATA2<-data.frame(cbind(Y2,L2,A1, Y2sq,L2sq ,A1sq,Y2L2))
DATA1<-data.frame(cbind(Y1,L1,A0, Y1sq,L1sq ,A0sq,Y1L1));DATA0<-data.frame(cbind(Y0,L0, Y0sq,L0sq ,Y0L0))
#Set initial values
beta43<-0.5; beta42<-0.3; beta41<-0.5; beta40<-0.3; beta32<-0.5; beta31<-0.5; beta30<-0.5; beta21<-0.3; beta20<-0.5; beta10<-0.5

V1<-var(Y4-predict(PredY43,DATA3)-(beta43*(A3-predict(PredA33,DATA3))))
V2<-var(Y4-predict(PredY42,DATA2)-(beta43*(A3-predict(PredA32,DATA2))+beta42*(A2-predict(PredA22,DATA2))))
V3<-var(Y4-predict(PredY41,DATA1)-(beta43*(A3-predict(PredA31,DATA1))+beta42*(A2-predict(PredA21,DATA1))+beta41*(A1-
predict(PredA11,DATA1))))
V4<-var(Y4-predict(PredY40,DATA0)-(beta43*(A3-predict(PredA30,DATA0))+beta42*(A2-predict(PredA20,DATA0))+beta41*(A1-
predict(PredA10,DATA0))+beta40*(A0-predict(PredA00,DATA0))))
V5<-var(Y3-predict(PredY32,DATA2)-(beta32*(A2-predict(PredA22,DATA2))))
V6<-var(Y3-predict(PredY31,DATA1)-(beta32*(A2-predict(PredA21,DATA1))+beta31*(A1-predict(PredA11,DATA1))))
V7<-var(Y3-predict(PredY30,DATA0)-(beta32*(A2-predict(PredA20,DATA0))+beta31*(A1-predict(PredA10,DATA0))+beta30*(A0-
predict(PredA00,DATA0))))
V8<-var(Y2-predict(PredY21,DATA1)-(beta21*(A1-predict(PredA11,DATA1))))
V9<-var(Y2-predict(PredY20,DATA0)-(beta21*(A1-predict(PredA10,DATA0))+beta20*(A0-predict(PredA00,DATA0))))
V10<-var(Y1-predict(PredY10,DATA0)-(beta10*(A0-predict(PredA00,DATA0))))
#Solve estimating equations via the Newton-Raphson Method
CHECK<-0; ITER3<-0; while(CHECK<1 && ITER3<200){
#First-order differentiation
dI<-rep(0,10); ITER3<-ITER3+1
dI[1]<-sum(
(A3-predict(PredA33,DATA3)) *(1/V1)*(Y4-predict(PredY43,DATA3)-(beta43*(A3-predict(PredA33,DATA3))))
)
dI[2]<-sum(
(A2-predict(PredA22,DATA2))*(1/V2)*(Y4-predict(PredY42,DATA2)-(beta43*(A3-predict(PredA32,DATA2))+beta42*(A2-predict(PredA22,DATA2))))
)
dI[3]<-sum(
(A1-predict(PredA11,DATA1))*(1/V3)*(Y4-predict(PredY41,DATA1)-(beta43*(A3-predict(PredA31,DATA1))+beta42*(A2-
predict(PredA21,DATA1))+beta41*(A1-predict(PredA11,DATA1))))
)
dI[4]<-sum(
(A0-predict(PredA00,DATA0))*(1/V4)*(Y4-predict(PredY40,DATA0)-(beta43*(A3-predict(PredA30,DATA0))+beta42*(A2-
predict(PredA20,DATA0))+beta41*(A1-predict(PredA10,DATA0))+beta40*(A0-predict(PredA00,DATA0))))
)

dI[5]<-sum(
(A2-predict(PredA22,DATA2)) *(1/V5)*(Y3-predict(PredY32,DATA2)-(beta32*(A2-predict(PredA22,DATA2))))
```

```
)
dI[6]<-sum(
(A1-predict(PredA11,DATA1))*(1/V6)*(Y3-predict(PredY31,DATA1)-(beta32*(A2-predict(PredA21,DATA1))+beta31*(A1-predict(PredA11,DATA1))))
)
dI[7]<-sum(
(A0-predict(PredA00,DATA0))*(1/V7)*(Y3-predict(PredY30,DATA0)-(beta32*(A2-predict(PredA20,DATA0))+beta31*(A1-
predict(PredA10,DATA0))+beta30*(A0-predict(PredA00,DATA0))))
)
dI[8]<-sum(
(A1-predict(PredA11,DATA1))*(1/V8)*(Y2-predict(PredY21,DATA1)-(beta21*(A1-predict(PredA11,DATA1))))
)
dI[9]<-sum(
(A0-predict(PredA00,DATA0))*(1/V9)*(Y2-predict(PredY20,DATA0)-(beta21*(A1-predict(PredA10,DATA0))+beta20*(A0-predict(PredA00,DATA0))))
)
dI[10]<-sum(
(A0-predict(PredA00,DATA0)) *(1/V10)*( Y1-predict(PredY10,DATA0)-(beta10*(A0-predict(PredA00,DATA0))))
)

#Second-order differentiation
dII<-rep(0,5)
dII[1]<-sum(
(A3-predict(PredA33,DATA3)) *(1/V1)* (A3-predict(PredA33,DATA3))
)
dII[2]<-sum(
(A2-predict(PredA22,DATA2)) *(1/V2)* (A2-predict(PredA22,DATA2))
)
dII[3]<-sum(
(A1-predict(PredA11,DATA1)) *(1/V3)* (A1-predict(PredA11,DATA1))
)
dII[4]<-sum(
(A0-predict(PredA00,DATA0)) *(1/V4)* (A0-predict(PredA00,DATA0))
)
dII[5]<-sum(
(A2-predict(PredA22,DATA2)) *(1/V5)* (A2-predict(PredA22,DATA2))
)
dII[6]<-sum(
(A1-predict(PredA11,DATA1)) *(1/V6)* (A1-predict(PredA11,DATA1))
)
dII[7]<-sum(
(A0-predict(PredA00,DATA0)) *(1/V7)* (A0-predict(PredA00,DATA0))
)
dII[8]<-sum(
(A1-predict(PredA11,DATA1)) *(1/V8)* (A1-predict(PredA11,DATA1))
)
dII[9]<-sum(
(A0-predict(PredA00,DATA0)) *(1/V9)* (A0-predict(PredA00,DATA0))
)
dII[10]<-sum(
(A0-predict(PredA00,DATA0)) *(1/V10)* (A0-predict(PredA00,DATA0))
)

#Update parameter values
PSI<- c(beta43,beta42,beta41,beta40,beta32,beta31,beta30,beta21,beta20,beta10);NEW<-PSI+diag(1/dII)%*%dI
#Check the convergence criterion
CHECK<-ifelse(max(abs(PSI-NEW))<0.0001,1,0)
beta43<-NEW[1] ; beta42<-NEW[2]; beta41<-NEW[3] ; beta40<-NEW[4] ; beta32<-NEW[5]; beta31<-NEW[6]
beta30<-NEW[7] ; beta21<-NEW[8]; beta20<-NEW[9] ; beta10<-NEW[10]
V1<-var(Y4-predict(PredY43,DATA3)-(beta43*(A3-predict(PredA33,DATA3))))
V2<-var(Y4-predict(PredY42,DATA2)-(beta43*(A3-predict(PredA32,DATA2))+beta42*(A2-predict(PredA22,DATA2))))
V3<-var(Y4-predict(PredY41,DATA1)-(beta43*(A3-predict(PredA31,DATA1))+beta42*(A2-predict(PredA21,DATA1))+beta41*(A1-
predict(PredA11,DATA1))))
V4<-var(Y4-predict(PredY40,DATA0)-(beta43*(A3-predict(PredA30,DATA0))+beta42*(A2-predict(PredA20,DATA0))+beta41*(A1-
predict(PredA10,DATA0))+beta40*(A0-predict(PredA00,DATA0))))
V5<-var(Y3-predict(PredY32,DATA2)-(beta32*(A2-predict(PredA22,DATA2))))
V6<-var(Y3-predict(PredY31,DATA1)-(beta32*(A2-predict(PredA21,DATA1))+beta31*(A1-predict(PredA11,DATA1))))
V7<-var(Y3-predict(PredY30,DATA0)-(beta32*(A2-predict(PredA20,DATA0))+beta31*(A1-predict(PredA10,DATA0))+beta30*(A0-
predict(PredA00,DATA0))))
V8<-var(Y2-predict(PredY21,DATA1)-(beta21*(A1-predict(PredA11,DATA1))))
V9<-var(Y2-predict(PredY20,DATA0)-(beta21*(A1-predict(PredA10,DATA0))+beta20*(A0-predict(PredA00,DATA0))))
V10<-var(Y1-predict(PredY10,DATA0)-(beta10*(A0-predict(PredA00,DATA0))))
};
#Store estimates and SEs
PSI3<-PSI;SE3<-sqrt(1/dII)
```

```r
#Proposed method
Y0<-    FYwith2[,1]
Y1<-    FYwith2[,2]
Y2<-    FYwith2[,3]
Y3<-    FYwith2[,4]
Y4<-    FYwith2[,5]
L0<-    FYwith2[,10]
L1<-    FYwith2[,11]
L2<-    FYwith2[,12]
L3<-    FYwith2[,13]
A0<-    FYwith2[,6]
A1<-    FYwith2[,7]
A2<-    FYwith2[,8]
A3<-    FYwith2[,9]

Y3sq<-Y3^2;L3sq<-L3^2;A2sq<-A2^2; Y2sq<-Y2^2;L2sq<-L2^2;A1sq<-A1^2; Y1sq<-Y1^2;L1sq<-L1^2;A0sq<-A0^2; Y0sq<-Y0^2;L0sq<-L0^2;
Y3L3<-Y3*L3; Y2L2<-Y2*L2; Y1L1<-Y1*L1; Y0L0<-Y0*L0;
Y3A3<-Y3*A3; Y2A2<-Y2*A2; Y1A1<-Y1*A1; Y0A0<-Y0*A0;
L3A3<-L3*A3; L2A2<-L2*A2; L1A1<-L1*A1; L0A0<-L0*A0;
#Estimate P(Y|A,L)
PredY43<-lm(Y4~ Y3+L3+A2+ Y3sq+L3sq +A2sq+Y3L3)
PredY42<-lm(Y4~ Y2+L2+A1+ Y2sq+L2sq +A1sq+Y2L2)
PredY41<-lm(Y4~ Y1+L1+A0+ Y1sq+L1sq +A0sq+Y1L1)
PredY40<-lm(Y4~ Y0+L0+Y0sq+L0sq+Y0L0)
PredY32<-lm(Y3~ Y2+L2+A1+ Y2sq+L2sq +A1sq+Y2L2)
PredY31<-lm(Y3~ Y1+L1+A0+ Y1sq+L1sq +A0sq+Y1L1)
PredY30<-lm(Y3~ Y0+L0+Y0sq+L0sq+Y0L0)
PredY21<-lm(Y2~ Y1+L1+A0+ Y1sq+L1sq +A0sq+Y1L1)
PredY20<-lm(Y2~ Y0+L0+Y0sq+L0sq+Y0L0)
PredY10<-lm(Y1~ Y0+L0+Y0sq+L0sq +Y0L0)
#Estimate P(A|A,L)
PredA33<-lm(A3~ Y3+L3+A2+ L3sq +A2sq+Y3L3)
PredA32<-lm(A3~ Y2+L2+A1+ L2sq +A1sq+Y2L2)
PredA31<-lm(A3~ Y1+L1+A0+ L1sq +A0sq+Y1L1)
PredA30<-lm(A3~ Y0+L0+L0sq+Y0L0)
PredA22<-lm(A2~ Y2+L2+A1+L2sq +A1sq+Y2L2)
PredA21<-lm(A2~ Y1+L1+A0+L1sq +A0sq+Y1L1)
PredA20<-lm(A2~ Y0+L0+L0sq+Y0L0)
PredA11<-lm(A1~ Y1+L1+A0+L1sq +A0sq+Y1L1)
PredA10<-lm(A1~ Y0+L0+L0sq+Y0L0)
PredA00<-lm(A0~ Y0+L0+L0sq+Y0L0)

DATA3<-data.frame(cbind(Y3,L3,A2, Y3sq,L3sq ,A2sq,Y3L3));DATA2<-data.frame(cbind(Y2,L2,A1, Y2sq,L2sq ,A1sq,Y2L2))
DATA1<-data.frame(cbind(Y1,L1,A0, Y1sq,L1sq ,A0sq,Y1L1));DATA0<-data.frame(cbind(Y0,L0, Y0sq,L0sq ,Y0L0))
#Set initial values
beta43<-0.5; beta42<-0.3; beta41<-0.5; beta40<-0.3; beta32<-0.5; beta31<-0.5; beta30<-0.5; beta21<-0.3; beta20<-0.5; beta10<-0.5

V1<-var(Y4-predict(PredY43,DATA3)-(beta43*(A3-predict(PredA33,DATA3))))
V2<-var(Y4-predict(PredY42,DATA2)-(beta43*(A3-predict(PredA32,DATA2))+beta42*(A2-predict(PredA22,DATA2))))
V3<-var(Y4-predict(PredY41,DATA1)-(beta43*(A3-predict(PredA31,DATA1))+beta42*(A2-predict(PredA21,DATA1))+beta41*(A1-
predict(PredA11,DATA1))))
V4<-var(Y4-predict(PredY40,DATA0)-(beta43*(A3-predict(PredA30,DATA0))+beta42*(A2-predict(PredA20,DATA0))+beta41*(A1-
predict(PredA10,DATA0))+beta40*(A0-predict(PredA00,DATA0))))
V5<-var(Y3-predict(PredY32,DATA2)-(beta32*(A2-predict(PredA22,DATA2))))
V6<-var(Y3-predict(PredY31,DATA1)-(beta32*(A2-predict(PredA21,DATA1))+beta31*(A1-predict(PredA11,DATA1))))
V7<-var(Y3-predict(PredY30,DATA0)-(beta32*(A2-predict(PredA20,DATA0))+beta31*(A1-predict(PredA10,DATA0))+beta30*(A0-
predict(PredA00,DATA0))))
V8<-var(Y2-predict(PredY21,DATA1)-(beta21*(A1-predict(PredA11,DATA1))))
V9<-var(Y2-predict(PredY20,DATA0)-(beta21*(A1-predict(PredA10,DATA0))+beta20*(A0-predict(PredA00,DATA0))))
V10<-var(Y1-predict(PredY10,DATA0)-(beta10*(A0-predict(PredA00,DATA0))))
#Solve estimating equations via the Newton-Raphson Method
CHECK<-0; ITER4<-0; while(CHECK<1 && ITER4<200){
#First-order differentiation
dI<-rep(0,10); ITER4<-ITER4+1
dI[1]<-sum(
(A3-predict(PredA33,DATA3)) *(1/V1)*(Y4-predict(PredY43,DATA3)-(beta43*(A3-predict(PredA33,DATA3))))
)
dI[2]<-sum(
(A2-predict(PredA22,DATA2))*(1/V2)*(Y4-predict(PredY42,DATA2)-(beta43*(A3-predict(PredA32,DATA2))+beta42*(A2-predict(PredA22,DATA2))))
)
dI[3]<-sum(
(A1-predict(PredA11,DATA1))*(1/V3)*(Y4-predict(PredY41,DATA1)-(beta43*(A3-predict(PredA31,DATA1))+beta42*(A2-
predict(PredA21,DATA1))+beta41*(A1-predict(PredA11,DATA1))))
)
dI[4]<-sum(
(A0-predict(PredA00,DATA0))*(1/V4)*(Y4-predict(PredY40,DATA0)-(beta43*(A3-predict(PredA30,DATA0))+beta42*(A2-
```

```
predict(PredA20,DATA0))+beta41*(A1-predict(PredA10,DATA0))+beta40*(A0-predict(PredA00,DATA0))))
)

dI[5]<-sum(
(A2-predict(PredA22,DATA2)) *(1/V5)*(Y3-predict(PredY32,DATA2)-(beta32*(A2-predict(PredA22,DATA2))))
)
dI[6]<-sum(
(A1-predict(PredA11,DATA1))*(1/V6)*(Y3-predict(PredY31,DATA1)-(beta32*(A2-predict(PredA21,DATA1))+beta31*(A1-predict(PredA11,DATA1))))
)
dI[7]<-sum(
(A0-predict(PredA00,DATA0))*(1/V7)*(Y3-predict(PredY30,DATA0)-(beta32*(A2-predict(PredA20,DATA0))+beta31*(A1-
predict(PredA10,DATA0))+beta30*(A0-predict(PredA00,DATA0))))
)
dI[8]<-sum(
(A1-predict(PredA11,DATA1))*(1/V8)*(Y2-predict(PredY21,DATA1)-(beta21*(A1-predict(PredA11,DATA1))))
)
dI[9]<-sum(
(A0-predict(PredA00,DATA0))*(1/V9)*(Y2-predict(PredY20,DATA0)-(beta21*(A1-predict(PredA10,DATA0))+beta20*(A0-predict(PredA00,DATA0))))
)
dI[10]<-sum(
(A0-predict(PredA00,DATA0)) *(1/V10)*( Y1-predict(PredY10,DATA0)-(beta10*(A0-predict(PredA00,DATA0))))
)
#Second-order differentiation
dII<-rep(0,5)
dII[1]<-sum(
(A3-predict(PredA33,DATA3)) *(1/V1)* (A3-predict(PredA33,DATA3))
)
dII[2]<-sum(
(A2-predict(PredA22,DATA2)) *(1/V2)* (A2-predict(PredA22,DATA2))
)
dII[3]<-sum(
(A1-predict(PredA11,DATA1)) *(1/V3)* (A1-predict(PredA11,DATA1))
)
dII[4]<-sum(
(A0-predict(PredA00,DATA0)) *(1/V4)* (A0-predict(PredA00,DATA0))
)
dII[5]<-sum(
(A2-predict(PredA22,DATA2)) *(1/V5)* (A2-predict(PredA22,DATA2))
)
dII[6]<-sum(
(A1-predict(PredA11,DATA1)) *(1/V6)* (A1-predict(PredA11,DATA1))
)
dII[7]<-sum(
(A0-predict(PredA00,DATA0)) *(1/V7)* (A0-predict(PredA00,DATA0))
)
dII[8]<-sum(
(A1-predict(PredA11,DATA1)) *(1/V8)* (A1-predict(PredA11,DATA1))
)
dII[9]<-sum(
(A0-predict(PredA00,DATA0)) *(1/V9)* (A0-predict(PredA00,DATA0))
)
dII[10]<-sum(
(A0-predict(PredA00,DATA0)) *(1/V10)* (A0-predict(PredA00,DATA0))
)
#Update parameter values
PSI<- c(beta43,beta42,beta41,beta40,beta32,beta31,beta30,beta21,beta20,beta10);NEW<-PSI+diag(1/dII)%*%dI
#Check the convergence criterion
CHECK<-ifelse(max(abs(PSI-NEW))<0.0001,1,0)
beta43<-NEW[1] ; beta42<-NEW[2]; beta41<-NEW[3] ; beta40<-NEW[4] ; beta32<-NEW[5]; beta31<-NEW[6]
beta30<-NEW[7] ; beta21<-NEW[8]; beta20<-NEW[9] ; beta10<-NEW[10]
V1<-var(Y4-predict(PredY43,DATA3)-(beta43*(A3-predict(PredA33,DATA3))))
V2<-var(Y4-predict(PredY42,DATA2)-(beta43*(A3-predict(PredA32,DATA2))+beta42*(A2-predict(PredA22,DATA2))))
V3<-var(Y4-predict(PredY41,DATA1)-(beta43*(A3-predict(PredA31,DATA1))+beta42*(A2-predict(PredA21,DATA1))+beta41*(A1-
predict(PredA11,DATA1))))
V4<-var(Y4-predict(PredY40,DATA0)-(beta43*(A3-predict(PredA30,DATA0))+beta42*(A2-predict(PredA20,DATA0))+beta41*(A1-
predict(PredA10,DATA0))+beta40*(A0-predict(PredA00,DATA0))))
V5<-var(Y3-predict(PredY32,DATA2)-(beta32*(A2-predict(PredA22,DATA2))))
V6<-var(Y3-predict(PredY31,DATA1)-(beta32*(A2-predict(PredA21,DATA1))+beta31*(A1-predict(PredA11,DATA1))))
V7<-var(Y3-predict(PredY30,DATA0)-(beta32*(A2-predict(PredA20,DATA0))+beta31*(A1-predict(PredA10,DATA0))+beta30*(A0-
predict(PredA00,DATA0))))
V8<-var(Y2-predict(PredY21,DATA1)-(beta21*(A1-predict(PredA11,DATA1))))
V9<-var(Y2-predict(PredY20,DATA0)-(beta21*(A1-predict(PredA10,DATA0))+beta20*(A0-predict(PredA00,DATA0))))
V10<-var(Y1-predict(PredY10,DATA0)-(beta10*(A0-predict(PredA00,DATA0))))
};
#Store estimates and SEs
PSI4<-PSI;SE4<-sqrt(1/dII)
```

```
#(Univariate) correlation-preserving factor score centering condition
Y0<-     (YY0-mean(YY0)-FYres)
Y1<-     (YY1-mean(YY1)-FYres)
Y2<-     (YY2-mean(YY2)-FYres)
Y3<-     (YY3-mean(YY3)-FYres)
Y4<-     (YY4-mean(YY4)-FYres)
L0<-     (LL0-mean(LL0)-FLres)
L1<-     (LL1-mean(LL1)-FLres)
L2<-     (LL2-mean(LL2)-FLres)
L3<-      (LL3-mean(LL3)-FLres)
A0<-   (AA0-mean(AA0)-FAres)
A1<-     (AA1-mean(AA1)-FAres)
A2<-     (AA2-mean(AA2)-FAres)
A3<-     (AA3-mean(AA3)-FAres)

Y3sq<-Y3^2;L3sq<-L3^2;A2sq<-A2^2; Y2sq<-Y2^2;L2sq<-L2^2;A1sq<-A1^2; Y1sq<-Y1^2;L1sq<-L1^2;A0sq<-A0^2; Y0sq<-Y0^2;L0sq<-L0^2;
Y3L3<-Y3*L3; Y2L2<-Y2*L2; Y1L1<-Y1*L1; Y0L0<-Y0*L0;
Y3A3<-Y3*A3; Y2A2<-Y2*A2; Y1A1<-Y1*A1; Y0A0<-Y0*A0;
L3A3<-L3*A3; L2A2<-L2*A2; L1A1<-L1*A1; L0A0<-L0*A0;
#Estimate P(Y|A,L)
PredY43<-lm(Y4~ Y3+L3+A2+ Y3sq+L3sq +A2sq+Y3L3)
PredY42<-lm(Y4~ Y2+L2+A1+ Y2sq+L2sq +A1sq+Y2L2)
PredY41<-lm(Y4~ Y1+L1+A0+ Y1sq+L1sq +A0sq+Y1L1)
PredY40<-lm(Y4~ Y0+L0+Y0sq+L0sq+Y0L0)
PredY32<-lm(Y3~ Y2+L2+A1+ Y2sq+L2sq +A1sq+Y2L2)
PredY31<-lm(Y3~ Y1+L1+A0+ Y1sq+L1sq +A0sq+Y1L1)
PredY30<-lm(Y3~ Y0+L0+Y0sq+L0sq+Y0L0)
PredY21<-lm(Y2~ Y1+L1+A0+ Y1sq+L1sq +A0sq+Y1L1)
PredY20<-lm(Y2~ Y0+L0+Y0sq+L0sq+Y0L0)
PredY10<-lm(Y1~ Y0+L0+Y0sq+L0sq +Y0L0)
#Estimate P(A|A,L)
PredA33<-lm(A3~ Y3+L3+A2+ L3sq +A2sq+Y3L3)
PredA32<-lm(A3~ Y2+L2+A1+ L2sq +A1sq+Y2L2)
PredA31<-lm(A3~ Y1+L1+A0+ L1sq +A0sq+Y1L1)
PredA30<-lm(A3~ Y0+L0+L0sq+Y0L0)
PredA22<-lm(A2~ Y2+L2+A1+L2sq +A1sq+Y2L2)
PredA21<-lm(A2~ Y1+L1+A0+L1sq +A0sq+Y1L1)
PredA20<-lm(A2~ Y0+L0+L0sq+Y0L0)
PredA11<-lm(A1~ Y1+L1+A0+L1sq +A0sq+Y1L1)
PredA10<-lm(A1~ Y0+L0+L0sq+Y0L0)
PredA00<-lm(A0~ Y0+L0+L0sq+Y0L0)


DATA3<-data.frame(cbind(Y3,L3,A2, Y3sq,L3sq ,A2sq,Y3L3));DATA2<-data.frame(cbind(Y2,L2,A1, Y2sq,L2sq ,A1sq,Y2L2))
DATA1<-data.frame(cbind(Y1,L1,A0, Y1sq,L1sq ,A0sq,Y1L1));DATA0<-data.frame(cbind(Y0,L0, Y0sq,L0sq ,Y0L0))
#Set initial values
beta43<-0.5; beta42<-0.3; beta41<-0.5; beta40<-0.3; beta32<-0.5; beta31<-0.5; beta30<-0.5; beta21<-0.3; beta20<-0.5; beta10<-0.5

V1<-var(Y4-predict(PredY43,DATA3)-(beta43*(A3-predict(PredA33,DATA3))))
V2<-var(Y4-predict(PredY42,DATA2)-(beta43*(A3-predict(PredA32,DATA2))+beta42*(A2-predict(PredA22,DATA2))))
V3<-var(Y4-predict(PredY41,DATA1)-(beta43*(A3-predict(PredA31,DATA1))+beta42*(A2-predict(PredA21,DATA1))+beta41*(A1-
predict(PredA11,DATA1))))
V4<-var(Y4-predict(PredY40,DATA0)-(beta43*(A3-predict(PredA30,DATA0))+beta42*(A2-predict(PredA20,DATA0))+beta41*(A1-
predict(PredA10,DATA0))+beta40*(A0-predict(PredA00,DATA0))))
V5<-var(Y3-predict(PredY32,DATA2)-(beta32*(A2-predict(PredA22,DATA2))))
V6<-var(Y3-predict(PredY31,DATA1)-(beta32*(A2-predict(PredA21,DATA1))+beta31*(A1-predict(PredA11,DATA1))))
V7<-var(Y3-predict(PredY30,DATA0)-(beta32*(A2-predict(PredA20,DATA0))+beta31*(A1-predict(PredA10,DATA0))+beta30*(A0-
predict(PredA00,DATA0))))
V8<-var(Y2-predict(PredY21,DATA1)-(beta21*(A1-predict(PredA11,DATA1))))
V9<-var(Y2-predict(PredY20,DATA0)-(beta21*(A1-predict(PredA10,DATA0))+beta20*(A0-predict(PredA00,DATA0))))
V10<-var(Y1-predict(PredY10,DATA0)-(beta10*(A0-predict(PredA00,DATA0))))
#Solve estimating equations via the Newton-Raphson Method
CHECK<-0; ITER5<-0; while(CHECK<1 && ITER5<200){
#First-order differentiation
dI<-rep(0,10); ITER5<-ITER5+1
dI[1]<-sum(
(A3-predict(PredA33,DATA3)) *(1/V1)*(Y4-predict(PredY43,DATA3)-(beta43*(A3-predict(PredA33,DATA3))))
)
dI[2]<-sum(
(A2-predict(PredA22,DATA2))*(1/V2)*(Y4-predict(PredY42,DATA2)-(beta43*(A3-predict(PredA32,DATA2))+beta42*(A2-predict(PredA22,DATA2))))
)
dI[3]<-sum(
(A1-predict(PredA11,DATA1))*(1/V3)*(Y4-predict(PredY41,DATA1)-(beta43*(A3-predict(PredA31,DATA1))+beta42*(A2-
predict(PredA21,DATA1))+beta41*(A1-predict(PredA11,DATA1))))
)
dI[4]<-sum(
```

```
(A0-predict(PredA00,DATA0))*(1/V4)*(Y4-predict(PredY40,DATA0)-(beta43*(A3-predict(PredA30,DATA0))+beta42*(A2-
predict(PredA20,DATA0))+beta41*(A1-predict(PredA10,DATA0))+beta40*(A0-predict(PredA00,DATA0))))
)
dI[5]<-sum(
(A2-predict(PredA22,DATA2)) *(1/V5)*(Y3-predict(PredY32,DATA2)-(beta32*(A2-predict(PredA22,DATA2))))
)
dI[6]<-sum(
(A1-predict(PredA11,DATA1))*(1/V6)*(Y3-predict(PredY31,DATA1)-(beta32*(A2-predict(PredA21,DATA1))+beta31*(A1-predict(PredA11,DATA1))))
)
dI[7]<-sum(
(A0-predict(PredA00,DATA0))*(1/V7)*(Y3-predict(PredY30,DATA0)-(beta32*(A2-predict(PredA20,DATA0))+beta31*(A1-
predict(PredA10,DATA0))+beta30*(A0-predict(PredA00,DATA0))))
)
dI[8]<-sum(
(A1-predict(PredA11,DATA1))*(1/V8)*(Y2-predict(PredY21,DATA1)-(beta21*(A1-predict(PredA11,DATA1))))
)
dI[9]<-sum(
(A0-predict(PredA00,DATA0))*(1/V9)*(Y2-predict(PredY20,DATA0)-(beta21*(A1-predict(PredA10,DATA0))+beta20*(A0-predict(PredA00,DATA0))))
)
dI[10]<-sum(
(A0-predict(PredA00,DATA0)) *(1/V10)*( Y1-predict(PredY10,DATA0)-(beta10*(A0-predict(PredA00,DATA0))))
)
dII<-rep(0,5)
#Second-order differentiation
dII[1]<-sum(
(A3-predict(PredA33,DATA3)) *(1/V1)* (A3-predict(PredA33,DATA3))
)
dII[2]<-sum(
(A2-predict(PredA22,DATA2)) *(1/V2)* (A2-predict(PredA22,DATA2))
)
dII[3]<-sum(
(A1-predict(PredA11,DATA1)) *(1/V3)* (A1-predict(PredA11,DATA1))
)
dII[4]<-sum(
(A0-predict(PredA00,DATA0)) *(1/V4)* (A0-predict(PredA00,DATA0))
)
dII[5]<-sum(
(A2-predict(PredA22,DATA2)) *(1/V5)* (A2-predict(PredA22,DATA2))
)
dII[6]<-sum(
(A1-predict(PredA11,DATA1)) *(1/V6)* (A1-predict(PredA11,DATA1))
)
dII[7]<-sum(
(A0-predict(PredA00,DATA0)) *(1/V7)* (A0-predict(PredA00,DATA0))
)
dII[8]<-sum(
(A1-predict(PredA11,DATA1)) *(1/V8)* (A1-predict(PredA11,DATA1))
)
dII[9]<-sum(
(A0-predict(PredA00,DATA0)) *(1/V9)* (A0-predict(PredA00,DATA0))
)
dII[10]<-sum(
(A0-predict(PredA00,DATA0)) *(1/V10)* (A0-predict(PredA00,DATA0))
)
#Update parameter values
PSI<- c(beta43,beta42,beta41,beta40,beta32,beta31,beta30,beta21,beta20,beta10);NEW<-PSI+diag(1/dII)%*%dI
#Check the convergence criterion
CHECK<-ifelse(max(abs(PSI-NEW))<0.0001,1,0)
beta43<-NEW[1] ; beta42<-NEW[2]; beta41<-NEW[3] ; beta40<-NEW[4] ; beta32<-NEW[5]; beta31<-NEW[6]
beta30<-NEW[7] ; beta21<-NEW[8]; beta20<-NEW[9] ; beta10<-NEW[10]
V1<-var(Y4-predict(PredY43,DATA3)-(beta43*(A3-predict(PredA33,DATA3))))
V2<-var(Y4-predict(PredY42,DATA2)-(beta43*(A3-predict(PredA32,DATA2))+beta42*(A2-predict(PredA22,DATA2))))
V3<-var(Y4-predict(PredY41,DATA1)-(beta43*(A3-predict(PredA31,DATA1))+beta42*(A2-predict(PredA21,DATA1))+beta41*(A1-
predict(PredA11,DATA1))))
V4<-var(Y4-predict(PredY40,DATA0)-(beta43*(A3-predict(PredA30,DATA0))+beta42*(A2-predict(PredA20,DATA0))+beta41*(A1-
predict(PredA10,DATA0))+beta40*(A0-predict(PredA00,DATA0))))
V5<-var(Y3-predict(PredY32,DATA2)-(beta32*(A2-predict(PredA22,DATA2))))
V6<-var(Y3-predict(PredY31,DATA1)-(beta32*(A2-predict(PredA21,DATA1))+beta31*(A1-predict(PredA11,DATA1))))
V7<-var(Y3-predict(PredY30,DATA0)-(beta32*(A2-predict(PredA20,DATA0))+beta31*(A1-predict(PredA10,DATA0))+beta30*(A0-
predict(PredA00,DATA0))))
V8<-var(Y2-predict(PredY21,DATA1)-(beta21*(A1-predict(PredA11,DATA1))))
V9<-var(Y2-predict(PredY20,DATA0)-(beta21*(A1-predict(PredA10,DATA0))+beta20*(A0-predict(PredA00,DATA0))))
V10<-var(Y1-predict(PredY10,DATA0)-(beta10*(A0-predict(PredA00,DATA0))))
};
#Store estimates and SEs
PSI5<-PSI;SE5<-sqrt(1/dII)
```

#Step 2 Estimate Causal Parameters by MSM

#True score condition

```
ZY_1<- dY0; ZY_2<- dY1; ZY_3<- dY2; ZY_4<- dY3
ZA_1<-dA0; ZA_2<-dA1; ZA_3<-dA2; ZA_4<-dA3;
ZL_1<-dL0; ZL_2<-dL1; ZL_3<-dL2; ZL_4<-dL3
ZLL_2<-dA0; ZLL_3<-dA1; ZLL_4<-dA2
ZZY_1<- dY1;ZZY_2<- dY2;ZZY_3<- dY3;ZZY_4<- dY4
ZZA_1<-dA0; ZZA_2<-dA1; ZZA_3<-dA2; ZZA_4<-dA3;

id<-c(1:N);data1 <- data.frame(id,ZY_1,ZA_1,ZL_1)
data_long<-reshape(data1, varying=c("ZY_1", "ZA_1", "ZL_1"), direction="long", idvar="id", sep="_")
data_long_sort <- arrange(data_long, id, time)

#Calculate weight for A0
w <- ipwtm(exposure = ZA,family = "gaussian",link = "logit",numerator = ~ 1,
   denominator = ~ ZL+ZY,
   id = id,timevar=time,corstr="ar1", type="all",data = data_long_sort)
iptw1 = c(w$ipw.weights)

iptww<-iptw1; data2 <- data.frame(id,ZZY_1,ZZA_1,iptww)
#Estimate causal effect β10 (for outcome at k=1)
gee.iptw <- lm(ZZY_1~ZZA_1,data=data2, weights=iptww)
out<-summary(gee.iptw) ; M10est<-out$coefficients[2,1]

data1 <- data.frame(id,ZY_2 ,ZA_2,ZL_2,ZLL_2)
data_long<-reshape(data1, varying=c("ZY_2","ZA_2","ZL_2","ZLL_2"), direction="long", idvar="id", sep="_")
data_long_sort <- arrange(data_long, id, time)

#Calculate weight for A1
w <- ipwtm(exposure = ZA,family = "gaussian",link = "logit",numerator = ~ ZLL,
   denominator = ~ ZL+ZY+ZLL,
   id = id,timevar=time,corstr="ar1", type="all",data = data_long_sort)
iptw2 = c(w$ipw.weights)

iptww<-iptw1*iptw2; data2 <- data.frame(id,ZZY_2,ZZA_1,ZZA_2,iptww)
#Estimate causal effects β20 and β21 (for outcome at k=2)
gee.iptw <- lm(ZZY_2~ZZA_2+ZZA_1,data=data2, weights=iptww)
out<-summary(gee.iptw) ; M20est<-out$coefficients[2,1]; M21est<-out$coefficients[3,1];


data1 <- data.frame(id,ZY_3 ,ZA_3,ZL_3,ZLL_3)
data_long<-reshape(data1, varying=c("ZY_3","ZA_3","ZL_3","ZLL_3"), direction="long", idvar="id", sep="_")
data_long_sort <- arrange(data_long, id, time)

#Calculate weight for A2
w <- ipwtm(exposure = ZA,family = "gaussian",link = "logit",numerator = ~ ZLL,
   denominator = ~ ZL+ZY+ZLL,
   id = id,timevar=time,corstr="ar1", type="all",data = data_long_sort)
iptw3= c(w$ipw.weights)

iptww<- iptw1*iptw2*iptw3; data2 <- data.frame(id,ZZY_3, ZZA_1,ZZA_2,ZZA_3,iptww)
#Estimate causal effects β30, β31, and β32 (for outcome at k=3)
gee.iptw <- lm(ZZY_3~ZZA_3+ZZA_2+ZZA_1,data=data2, weights=iptww)
out<-summary(gee.iptw) ; M30est<-out$coefficients[2,1]; M31est<-out$coefficients[3,1];M32est<-out$coefficients[4,1];


data1 <- data.frame(id,ZY_4 ,ZA_4,ZL_4,ZLL_4)
data_long<-reshape(data1, varying=c("ZY_4","ZA_4","ZL_4","ZLL_4"), direction="long", idvar="id", sep="_")
data_long_sort <- arrange(data_long, id, time)

#Calculate weight for A3
w <- ipwtm(exposure = ZA,family = "gaussian",link = "logit",numerator = ~ ZLL,
   denominator = ~ ZL+ZY+ZLL,
   id = id,timevar=time,corstr="ar1", type="all",data = data_long_sort)
iptw4= c(w$ipw.weights)

iptww<- iptw1*iptw2*iptw3*iptw4; data2 <- data.frame(id,ZZY_4,ZZA_1,ZZA_2,ZZA_3,ZZA_4,iptww)
#Estimate causal effects   β40, β41, β42, and β43 (for outcome at k=4)
gee.iptw <- lm(ZZY_4~ZZA_4+ZZA_3+ZZA_2+ZZA_1,data=data2, weights=iptww)
out<-summary(gee.iptw) ; M40est<-out$coefficients[2,1]; M41est<-out$coefficients[3,1];M42est<-out$coefficients[4,1] ;M43est<-out$coefficients[5,1]

#Store estimation results
MPSI1<-c(M40est,M41est,M42est,M43est,M30est,M31est,M32est,M20est,M21est,M10est)
```

```
#Observed score (no centering) condition
ZY_1<-     YY0-mean(YY0);ZY_2<-     YY1-mean(YY1);ZY_3<-     YY2-mean(YY2);ZY_4<-     YY3-mean(YY3)
ZA_1<-AA0-mean(AA0); ZA_2<-AA1-mean(AA1); ZA_3<- AA2-mean(AA2); ZA_4<- AA3-mean(AA3);
ZL_1<- LL0-mean(LL0); ZL_2<- LL1-mean(LL1); ZL_3<- LL2-mean(LL2); ZL_4<- LL3-mean(LL3)
ZLL_2<- AA0-mean(AA0); ZLL_3<- AA1-mean(AA1); ZLL_4<- AA2-mean(AA2)
ZZY_1<- YY1-mean(YY1);ZZY_2<- YY2-mean(YY2);ZZY_3<- YY3-mean(YY3);ZZY_4<- YY4-mean(YY4)
ZZA_1<- AA0-mean(AA0); ZZA_2<- AA1-mean(AA1); ZZA_3<- AA2-mean(AA2); ZZA_4<- AA3-mean(AA3);

id<-c(1:N);data1 <- data.frame(id,ZY_1,ZA_1,ZL_1)
data_long<-reshape(data1, varying=c("ZY_1", "ZA_1", "ZL_1"), direction="long", idvar="id", sep="_")
data_long_sort <- arrange(data_long, id, time)

#Calculate weight for A0
w <- ipwtm(exposure = ZA,family = "gaussian",link = "logit",numerator = ~ 1,
   denominator = ~ ZL+ZY,
   id = id,timevar=time,corstr="ar1", type="all",data = data_long_sort)
iptw1 = c(w$ipw.weights)

iptww<-iptw1; data2 <- data.frame(id,ZZY_1,ZZA_1,iptww)
#Estimate causal effect β10 (for outcome at k=1)
gee.iptw <- lm(ZZY_1~ZZA_1,data=data2, weights=iptww)
out<-summary(gee.iptw) ; M10est<-out$coefficients[2,1]

data1 <- data.frame(id,ZY_2 ,ZA_2,ZL_2,ZLL_2)
data_long<-reshape(data1, varying=c("ZY_2","ZA_2","ZL_2","ZLL_2"), direction="long", idvar="id", sep="_")
data_long_sort <- arrange(data_long, id, time)

#Calculate weight for A1
w <- ipwtm(exposure = ZA,family = "gaussian",link = "logit",numerator = ~ ZLL,
   denominator = ~ ZL+ZY+ZLL,
   id = id,timevar=time,corstr="ar1", type="all",data = data_long_sort)
iptw2 = c(w$ipw.weights)

iptww<-iptw1*iptw2; data2 <- data.frame(id,ZZY_2,ZZA_1,ZZA_2,iptww)
#Estimate causal effects β20 and β21 (for outcome at k=2)
gee.iptw <- lm(ZZY_2~ZZA_2+ZZA_1,data=data2, weights=iptww)
out<-summary(gee.iptw) ; M20est<-out$coefficients[2,1]; M21est<-out$coefficients[3,1];


data1 <- data.frame(id,ZY_3 ,ZA_3,ZL_3,ZLL_3)
data_long<-reshape(data1, varying=c("ZY_3","ZA_3","ZL_3","ZLL_3"), direction="long", idvar="id", sep="_")
data_long_sort <- arrange(data_long, id, time)

#Calculate weight for A2
w <- ipwtm(exposure = ZA,family = "gaussian",link = "logit",numerator = ~ ZLL,
   denominator = ~ ZL+ZY+ZLL,
   id = id,timevar=time,corstr="ar1", type="all",data = data_long_sort)
iptw3= c(w$ipw.weights)

iptww<- iptw1*iptw2*iptw3; data2 <- data.frame(id,ZZY_3, ZZA_1,ZZA_2,ZZA_3,iptww)
#Estimate causal effects β30, β31, and β32 (for outcome at k=3)
gee.iptw <- lm(ZZY_3~ZZA_3+ZZA_2+ZZA_1,data=data2, weights=iptww)
out<-summary(gee.iptw) ; M30est<-out$coefficients[2,1]; M31est<-out$coefficients[3,1];M32est<-out$coefficients[4,1];


data1 <- data.frame(id,ZY_4 ,ZA_4,ZL_4,ZLL_4)
data_long<-reshape(data1, varying=c("ZY_4","ZA_4","ZL_4","ZLL_4"), direction="long", idvar="id", sep="_")
data_long_sort <- arrange(data_long, id, time)

#Calculate weight for A3
w <- ipwtm(exposure = ZA,family = "gaussian",link = "logit",numerator = ~ ZLL,
   denominator = ~ ZL+ZY+ZLL,
   id = id,timevar=time,corstr="ar1", type="all",data = data_long_sort)
iptw4= c(w$ipw.weights)

iptww<- iptw1*iptw2*iptw3*iptw4; data2 <- data.frame(id,ZZY_4,ZZA_1,ZZA_2,ZZA_3,ZZA_4,iptww)
#Estimate causal effect    β40, β41, β42, and β43 (for outcome at k=4)
gee.iptw <- lm(ZZY_4~ZZA_4+ZZA_3+ZZA_2+ZZA_1,data=data2, weights=iptww)
out<-summary(gee.iptw) ; M40est<-out$coefficients[2,1]; M41est<-out$coefficients[3,1];M42est<-out$coefficients[4,1] ;M43est<-out$coefficients[5,1]

#Store estimation results
MPSI2<-c(M40est,M41est,M42est,M43est,M30est,M31est,M32est,M20est,M21est,M10est)
```

```
#Observed-mean centering condition
MY<-rowMeans(cbind(YY0,YY1,YY2,YY3,YY4)); ML<-rowMeans(cbind(LL0,LL1,LL2,LL3)); MA<-rowMeans(cbind(AA0,AA1,AA2,AA3))
ZY_1<-      YY0-mean(YY0)-MY;ZY_2<-      YY1-mean(YY1) -MY;ZY_3<-      YY2-mean(YY2) -MY;ZY_4<-      YY3-mean(YY3) -MY
ZA_1<-AA0-mean(AA0)-MA; ZA_2<-AA1-mean(AA1)-MA; ZA_3<- AA2-mean(AA2)-MA; ZA_4<- AA3-mean(AA3)-MA;
ZL_1<- LL0-mean(LL0)-ML; ZL_2<- LL1-mean(LL1)-ML; ZL_3<- LL2-mean(LL2)-ML; ZL_4<- LL3-mean(LL3)-ML
ZLL_2<- AA0-mean(AA0)-MA; ZLL_3<- AA1-mean(AA1)-MA; ZLL_4<- AA2-mean(AA2)-MA
ZZY_1<- YY1-mean(YY1) -MY;ZZY_2<- YY2-mean(YY2) -MY;ZZY_3<- YY3-mean(YY3) -MY;ZZY_4<- YY4-mean(YY4) -MY
ZZA_1<- AA0-mean(AA0)-MA; ZZA_2<- AA1-mean(AA1)-MA; ZZA_3<- AA2-mean(AA2)-MA; ZZA_4<- AA3-mean(AA3)-MA;

id<-c(1:N);data1 <- data.frame(id,ZY_1,ZA_1,ZL_1)
data_long<-reshape(data1, varying=c("ZY_1", "ZA_1", "ZL_1"), direction="long", idvar="id", sep="_")
data_long_sort <- arrange(data_long, id, time)

#Calculate weight for A0
w <- ipwtm(exposure = ZA,family = "gaussian",link = "logit",numerator = ~ 1,
   denominator = ~ ZL+ZY,
   id = id,timevar=time,corstr="ar1", type="all",data = data_long_sort)
iptw1 = c(w$ipw.weights)

iptww<-iptw1; data2 <- data.frame(id,ZZY_1,ZZA_1,iptww)
#Estimate causal effect β10 (for outcome at k=1)
gee.iptw <- lm(ZZY_1~ZZA_1,data=data2, weights=iptww)
out<-summary(gee.iptw) ; M10est<-out$coefficients[2,1]

data1 <- data.frame(id,ZY_2 ,ZA_2,ZL_2,ZLL_2)
data_long<-reshape(data1, varying=c("ZY_2","ZA_2","ZL_2","ZLL_2"), direction="long", idvar="id", sep="_")
data_long_sort <- arrange(data_long, id, time)

#Calculate weight for A1
w <- ipwtm(exposure = ZA,family = "gaussian",link = "logit",numerator = ~ ZLL,
   denominator = ~ ZL+ZY+ZLL,
   id = id,timevar=time,corstr="ar1", type="all",data = data_long_sort)
iptw2 = c(w$ipw.weights)

iptww<-iptw1*iptw2; data2 <- data.frame(id,ZZY_2,ZZA_1,ZZA_2,iptww)
#Estimate causal effects β20 and β21 (for outcome at k=2)
gee.iptw <- lm(ZZY_2~ZZA_2+ZZA_1,data=data2, weights=iptww)
out<-summary(gee.iptw) ; M20est<-out$coefficients[2,1]; M21est<-out$coefficients[3,1];


data1 <- data.frame(id,ZY_3 ,ZA_3,ZL_3,ZLL_3)
data_long<-reshape(data1, varying=c("ZY_3","ZA_3","ZL_3","ZLL_3"), direction="long", idvar="id", sep="_")
data_long_sort <- arrange(data_long, id, time)

#Calculate weight for A2
w <- ipwtm(exposure = ZA,family = "gaussian",link = "logit",numerator = ~ ZLL,
   denominator = ~ ZL+ZY+ZLL,
   id = id,timevar=time,corstr="ar1", type="all",data = data_long_sort)
iptw3= c(w$ipw.weights)

iptww<- iptw1*iptw2*iptw3; data2 <- data.frame(id,ZZY_3, ZZA_1,ZZA_2,ZZA_3,iptww)
#Estimate causal effects β30, β31, and β32 (for outcome at k=3)
gee.iptw <- lm(ZZY_3~ZZA_3+ZZA_2+ZZA_1,data=data2, weights=iptww)
out<-summary(gee.iptw) ; M30est<-out$coefficients[2,1]; M31est<-out$coefficients[3,1];M32est<-out$coefficients[4,1];


data1 <- data.frame(id,ZY_4 ,ZA_4,ZL_4,ZLL_4)
data_long<-reshape(data1, varying=c("ZY_4","ZA_4","ZL_4","ZLL_4"), direction="long", idvar="id", sep="_")
data_long_sort <- arrange(data_long, id, time)

#Calculate weight for A3
w <- ipwtm(exposure = ZA,family = "gaussian",link = "logit",numerator = ~ ZLL,
   denominator = ~ ZL+ZY+ZLL,
   id = id,timevar=time,corstr="ar1", type="all",data = data_long_sort)
iptw4= c(w$ipw.weights)

iptww<- iptw1*iptw2*iptw3*iptw4; data2 <- data.frame(id,ZZY_4,ZZA_1,ZZA_2,ZZA_3,ZZA_4,iptww)
#Estimate causal effects    β40, β41, β42, and β43 (for outcome at k=4)
gee.iptw <- lm(ZZY_4~ZZA_4+ZZA_3+ZZA_2+ZZA_1,data=data2, weights=iptww)
out<-summary(gee.iptw) ; M40est<-out$coefficients[2,1]; M41est<-out$coefficients[3,1];M42est<-out$coefficients[4,1] ;M43est<-out$coefficients[5,1]

#Store estimation results
MPSI3<-c(M40est,M41est,M42est,M43est,M30est,M31est,M32est,M20est,M21est,M10est)
```

```
#Proposed method
ZY_1<-   FYwith2[,1]; ZY_2<-   FYwith2[,2]; ZY_3<-   FYwith2[,3]; ZY_4<-   FYwith2[,4]
ZL_1<-   FYwith2[,10] ;ZL_2<-   FYwith2[,11]; ZL_3<-   FYwith2[,12]; ZL_4<-   FYwith2[,13]
ZA_1<-   FYwith2[,6]; ZA_2<-   FYwith2[,7]; ZA_3<-   FYwith2[,8]; ZA_4<-  FYwith2[,9]
ZLL_2<-   FYwith2[,6]; ZLL_3<-   FYwith2[,7]; ZLL_4<-   FYwith2[,8]
ZZY_1<-   FYwith2[,2]; ZZY_2<-   FYwith2[,3]; ZZY_3<-   FYwith2[,4]; ZZY_4<-   FYwith2[,5]
ZZA_1<-   FYwith2[,6]; ZZA_2<-   FYwith2[,7]; ZZA_3<-   FYwith2[,8]; ZZA_4<-  FYwith2[,9]

id<-c(1:N);data1 <- data.frame(id,ZY_1,ZA_1,ZL_1)
data_long<-reshape(data1, varying=c("ZY_1", "ZA_1", "ZL_1"), direction="long", idvar="id", sep="_")
data_long_sort <- arrange(data_long, id, time)

#Calculate weight for A0
w <- ipwtm(exposure = ZA,family = "gaussian",link = "logit",numerator = ~ 1,
  denominator = ~ ZL+ZY,
  id = id,timevar=time,corstr="ar1", type="all",data = data_long_sort)
iptw1 = c(w$ipw.weights)

iptww<-iptw1; data2 <- data.frame(id,ZZY_1,ZZA_1,iptww)
#Estimate causal effect β10 (for outcome at k=1)
gee.iptw <- lm(ZZY_1~ZZA_1,data=data2, weights=iptww)
out<-summary(gee.iptw) ; M10est<-out$coefficients[2,1]

data1 <- data.frame(id,ZY_2 ,ZA_2,ZL_2,ZLL_2)
data_long<-reshape(data1, varying=c("ZY_2","ZA_2","ZL_2","ZLL_2"), direction="long", idvar="id", sep="_")
data_long_sort <- arrange(data_long, id, time)

#Calculate weight for A1
w <- ipwtm(exposure = ZA,family = "gaussian",link = "logit",numerator = ~ ZLL,
  denominator = ~ ZL+ZY+ZLL,
  id = id,timevar=time,corstr="ar1", type="all",data = data_long_sort)
iptw2 = c(w$ipw.weights)

iptww<-iptw1*iptw2; data2 <- data.frame(id,ZZY_2,ZZA_1,ZZA_2,iptww)
#Estimate causal effects β20 and β21 (for outcome at k=2)
gee.iptw <- lm(ZZY_2~ZZA_2+ZZA_1,data=data2, weights=iptww)
out<-summary(gee.iptw) ; M20est<-out$coefficients[2,1]; M21est<-out$coefficients[3,1];


data1 <- data.frame(id,ZY_3 ,ZA_3,ZL_3,ZLL_3)
data_long<-reshape(data1, varying=c("ZY_3","ZA_3","ZL_3","ZLL_3"), direction="long", idvar="id", sep="_")
data_long_sort <- arrange(data_long, id, time)

#Calculate weight for A2
w <- ipwtm(exposure = ZA,family = "gaussian",link = "logit",numerator = ~ ZLL,
  denominator = ~ ZL+ZY+ZLL,
  id = id,timevar=time,corstr="ar1", type="all",data = data_long_sort)
iptw3= c(w$ipw.weights)

iptww<- iptw1*iptw2*iptw3; data2 <- data.frame(id,ZZY_3, ZZA_1,ZZA_2,ZZA_3,iptww)
#Estimate causal effects β30, β31, and β32 (for outcome at k=3)
gee.iptw <- lm(ZZY_3~ZZA_3+ZZA_2+ZZA_1,data=data2, weights=iptww)
out<-summary(gee.iptw) ; M30est<-out$coefficients[2,1]; M31est<-out$coefficients[3,1];M32est<-out$coefficients[4,1];


data1 <- data.frame(id,ZY_4 ,ZA_4,ZL_4,ZLL_4)
data_long<-reshape(data1, varying=c("ZY_4","ZA_4","ZL_4","ZLL_4"), direction="long", idvar="id", sep="_")
data_long_sort <- arrange(data_long, id, time)

#Calculate weight for A3
w <- ipwtm(exposure = ZA,family = "gaussian",link = "logit",numerator = ~ ZLL,
  denominator = ~ ZL+ZY+ZLL,
  id = id,timevar=time,corstr="ar1", type="all",data = data_long_sort)
iptw4= c(w$ipw.weights)

iptww<- iptw1*iptw2*iptw3*iptw4; data2 <- data.frame(id,ZZY_4,ZZA_1,ZZA_2,ZZA_3,ZZA_4,iptww)
#Estimate causal effects   β40, β41, β42, and β43 (for outcome at k=4)
gee.iptw <- lm(ZZY_4~ZZA_4+ZZA_3+ZZA_2+ZZA_1,data=data2, weights=iptww)
out<-summary(gee.iptw) ; M40est<-out$coefficients[2,1]; M41est<-out$coefficients[3,1];M42est<-out$coefficients[4,1] ;M43est<-out$coefficients[5,1]

#Store estimation results
MPSI4<-c(M40est,M41est,M42est,M43est,M30est,M31est,M32est,M20est,M21est,M10est)
```

```
#(Univariate) correlation-preserving factor score centering condition
ZY_1<-     (YY0-mean(YY0)-FYres)
ZY_2<-     (YY1-mean(YY1)-FYres)
ZY_3<-     (YY2-mean(YY2)-FYres)
ZY_4<-     (YY3-mean(YY3)-FYres)
ZA_1<-AA0-mean(AA0)-FAres; ZA_2<-AA1-mean(AA1)-FAres; ZA_3<- AA2-mean(AA2)-FAres; ZA_4<- AA3-mean(AA3)-FAres;
ZL_1<- LL0-mean(LL0)-FLres; ZL_2<- LL1-mean(LL1)-FLres; ZL_3<- LL2-mean(LL2)-FLres; ZL_4<- LL3-mean(LL3)-FLres
ZLL_2<- AA0-mean(AA0)-FAres; ZLL_3<- AA1-mean(AA1)-FAres; ZLL_4<- AA2-mean(AA2)-FAres
ZZY_1<-     (YY1-mean(YY1)-FYres)
ZZY_2<-     (YY2-mean(YY2)-FYres)
ZZY_3<-     (YY3-mean(YY3)-FYres)
ZZY_4<-     (YY4-mean(YY4)-FYres)
ZZA_1<- AA0-mean(AA0)-FAres; ZZA_2<- AA1-mean(AA1)-FAres; ZZA_3<- AA2-mean(AA2)-FAres; ZZA_4<- AA3-mean(AA3)-FAres;

id<-c(1:N);data1 <- data.frame(id,ZY_1,ZA_1,ZL_1)
data_long<-reshape(data1, varying=c("ZY_1", "ZA_1", "ZL_1"), direction="long", idvar="id", sep="_")
data_long_sort <- arrange(data_long, id, time)

#Calculate weight for A0
w <- ipwtm(exposure = ZA,family = "gaussian",link = "logit",numerator = ~ 1,
   denominator = ~ ZL+ZY,
   id = id,timevar=time,corstr="ar1", type="all",data = data_long_sort)
iptw1 = c(w$ipw.weights)

iptww<-iptw1; data2 <- data.frame(id,ZZY_1,ZZA_1,iptww)
#Estimate causal effect β10 (for outcome at k=1)
gee.iptw <- lm(ZZY_1~ZZA_1,data=data2, weights=iptww)
out<-summary(gee.iptw) ; M10est<-out$coefficients[2,1]

data1 <- data.frame(id,ZY_2 ,ZA_2,ZL_2,ZLL_2)
data_long<-reshape(data1, varying=c("ZY_2","ZA_2","ZL_2","ZLL_2"), direction="long", idvar="id", sep="_")
data_long_sort <- arrange(data_long, id, time)

#Calculate weight for A1
w <- ipwtm(exposure = ZA,family = "gaussian",link = "logit",numerator = ~ ZLL,
   denominator = ~ ZL+ZY+ZLL,
   id = id,timevar=time,corstr="ar1", type="all",data = data_long_sort)
iptw2 = c(w$ipw.weights)

iptww<-iptw1*iptw2; data2 <- data.frame(id,ZZY_2,ZZA_1,ZZA_2,iptww)
#Estimate causal effects β20 and β21 (for outcome at k=2)
gee.iptw <- lm(ZZY_2~ZZA_2+ZZA_1,data=data2, weights=iptww)
out<-summary(gee.iptw) ; M20est<-out$coefficients[2,1]; M21est<-out$coefficients[3,1];

data1 <- data.frame(id,ZY_3 ,ZA_3,ZL_3,ZLL_3)
data_long<-reshape(data1, varying=c("ZY_3","ZA_3","ZL_3","ZLL_3"), direction="long", idvar="id", sep="_")
data_long_sort <- arrange(data_long, id, time)

#Calculate weight for A2
w <- ipwtm(exposure = ZA,family = "gaussian",link = "logit",numerator = ~ ZLL,
   denominator = ~ ZL+ZY+ZLL,
   id = id,timevar=time,corstr="ar1", type="all",data = data_long_sort)
iptw3= c(w$ipw.weights)

iptww<- iptw1*iptw2*iptw3; data2 <- data.frame(id,ZZY_3, ZZA_1,ZZA_2,ZZA_3,iptww)
#Estimate causal effects β30, β31, and β32 (for outcome at k=3)
gee.iptw <- lm(ZZY_3~ZZA_3+ZZA_2+ZZA_1,data=data2, weights=iptww)
out<-summary(gee.iptw) ; M30est<-out$coefficients[2,1]; M31est<-out$coefficients[3,1];M32est<-out$coefficients[4,1];

data1 <- data.frame(id,ZY_4 ,ZA_4,ZL_4,ZLL_4)
data_long<-reshape(data1, varying=c("ZY_4","ZA_4","ZL_4","ZLL_4"), direction="long", idvar="id", sep="_")
data_long_sort <- arrange(data_long, id, time)

#Calculate weight for A3
w <- ipwtm(exposure = ZA,family = "gaussian",link = "logit",numerator = ~ ZLL,
   denominator = ~ ZL+ZY+ZLL,
   id = id,timevar=time,corstr="ar1", type="all",data = data_long_sort)
iptw4= c(w$ipw.weights)

iptww<- iptw1*iptw2*iptw3*iptw4; data2 <- data.frame(id,ZZY_4,ZZA_1,ZZA_2,ZZA_3,ZZA_4,iptww)
#Estimate causal effects    β40, β41, β42, and β43 (for outcome at k=4)
gee.iptw <- lm(ZZY_4~ZZA_4+ZZA_3+ZZA_2+ZZA_1,data=data2, weights=iptww)
out<-summary(gee.iptw) ; M40est<-out$coefficients[2,1]; M41est<-out$coefficients[3,1];M42est<-out$coefficients[4,1] ;M43est<-out$coefficients[5,1]

#Store estimation results
MPSI5<-c(M40est,M41est,M42est,M43est,M30est,M31est,M32est,M20est,M21est,M10est)
```

```
ITER<-ifelse(ITER1==200,0,1)+ ifelse(ITER2==200,0,1)+ ifelse(ITER3==200,0,1)+ ifelse(ITER5==200,0,1)
IMPMSM<-ifelse(is.na(prod(MPSI1,MPSI2,MPSI3,MPSI4,MPSI5)),0,1)

if(IMPRO1+IMPRO2+IMPRO3+ITER+IMPMSM>23){
#Y4A3 0.4
#Y4Y3A2 0.4*0.4#Y4L3A2 0.1*0.2
#Y4Y3Y2A1 0.4*0.4*0.4#Y4Y3L2A1 0.4*0.1*0.2#Y4L3Y2A1 0.1*0.2*0.4#Y4L3L2A1 0.1*0.5*0.2
#Y4Y3Y2Y1A0 0.4*0.4*0.4*0.4#Y4Y3Y2L1A0 0.4*0.4*0.1*0.2 #Y4Y3L2Y1A0 0.4*0.1*0.2*0.4 #Y4Y3L2L1A0 0.4*0.1*0.5*0.2
#Y4L3Y2Y1A0 0.1*0.2*0.4*0.4#Y4L3Y2L1A0 0.1*0.2*0.1*0.2
#Y4L3L2Y1A0 0.1*0.5*0.2*0.4#Y4L3L2L1A0 0.1*0.5*0.5*0.2
#True values of causal effects
PTRUE<-c(0.400,0.180,0.090,0.0486,0.400,0.180,0.090,0.400,0.180,0.400)

#Store point estimates results
POINTEST[3*(aaaa-1)+bbbb,1]<- POINTEST[3*(aaaa-1)+bbbb,1]+4
POINTEST[3*(aaaa-1)+bbbb,2]<- POINTEST[3*(aaaa-1)+bbbb,2]+aaaa
POINTEST[3*(aaaa-1)+bbbb,3]<- POINTEST[3*(aaaa-1)+bbbb,3]+bbbb
POINTEST[3*(aaaa-1)+bbbb,4:13]<- POINTEST[3*(aaaa-1)+bbbb,4:13]+PSI1 -PTRUE
POINTEST[3*(aaaa-1)+bbbb,14:23]<- POINTEST[3*(aaaa-1)+bbbb,14:23]+ PSI2 -PTRUE
POINTEST[3*(aaaa-1)+bbbb,24:33]<- POINTEST[3*(aaaa-1)+bbbb,24:33]+ PSI3 -PTRUE
POINTEST[3*(aaaa-1)+bbbb,34:43]<- POINTEST[3*(aaaa-1)+bbbb,34:43]+ PSI4 -PTRUE
POINTEST[3*(aaaa-1)+bbbb,44:53]<- POINTEST[3*(aaaa-1)+bbbb,44:53]+ PSI5 -PTRUE
POINTEST[3*(aaaa-1)+bbbb,54:63]<- POINTEST[3*(aaaa-1)+bbbb,54:63]+ MPSI1 -PTRUE
POINTEST[3*(aaaa-1)+bbbb,64:73]<- POINTEST[3*(aaaa-1)+bbbb,64:73]+ MPSI2 -PTRUE
POINTEST[3*(aaaa-1)+bbbb,74:83]<- POINTEST[3*(aaaa-1)+bbbb,74:83]+ MPSI3 -PTRUE
POINTEST[3*(aaaa-1)+bbbb,84:93]<- POINTEST[3*(aaaa-1)+bbbb,84:93]+ MPSI4 -PTRUE
POINTEST[3*(aaaa-1)+bbbb,94:103]<- POINTEST[3*(aaaa-1)+bbbb,94:103]+ MPSI5 -PTRUE

#Store SE estimates results
SEEST[3*(aaaa-1)+bbbb,1]<- SEEST[3*(aaaa-1)+bbbb,1]+4
SEEST[3*(aaaa-1)+bbbb,2]<- SEEST[3*(aaaa-1)+bbbb,2]+aaaa
SEEST[3*(aaaa-1)+bbbb,3]<- SEEST[3*(aaaa-1)+bbbb,3]+bbbb
SEEST[3*(aaaa-1)+bbbb,4:13]<- SEEST[3*(aaaa-1)+bbbb,4:13]+(PSI1 -PTRUE)^2
SEEST[3*(aaaa-1)+bbbb,14:23]<- SEEST[3*(aaaa-1)+bbbb,14:23]+ (PSI2 -PTRUE)^2
SEEST[3*(aaaa-1)+bbbb,24:33]<- SEEST[3*(aaaa-1)+bbbb,24:33]+ (PSI3 -PTRUE)^2
SEEST[3*(aaaa-1)+bbbb,34:43]<- SEEST[3*(aaaa-1)+bbbb,34:43]+ (PSI4 -PTRUE)^2
SEEST[3*(aaaa-1)+bbbb,44:53]<- SEEST[3*(aaaa-1)+bbbb,44:53]+ (PSI5 -PTRUE)^2
SEEST[3*(aaaa-1)+bbbb,54:63]<- SEEST[3*(aaaa-1)+bbbb,54:63]+ (MPSI1 -PTRUE)^2
SEEST[3*(aaaa-1)+bbbb,64:73]<- SEEST[3*(aaaa-1)+bbbb,64:73]+ (MPSI2 -PTRUE)^2
SEEST[3*(aaaa-1)+bbbb,74:83]<- SEEST[3*(aaaa-1)+bbbb,74:83]+ (MPSI3 -PTRUE)^2
SEEST[3*(aaaa-1)+bbbb,84:93]<- SEEST[3*(aaaa-1)+bbbb,84:93]+ (MPSI4 -PTRUE)^2
SEEST[3*(aaaa-1)+bbbb,94:103]<- SEEST[3*(aaaa-1)+bbbb,94:103]+ (MPSI5 -PTRUE)^2

COUNT<-COUNT+1
}else{;}
}else{;}

}
;};}

KKKK1<-read.csv("*****/pointest41.csv")[,-1]
if(KKKK1[1,5]>199){;q();}else{;}
KKKK1[,104]<-KKKK1[,104]+1
POINTEST<-POINTEST+KKKK1
write.csv(POINTEST, "*****/pointest41.csv")

KKKK2<-read.csv("*****/seest41.csv")[,-1]
KKKK2[,104]<-KKKK2[,104]+1
SEEST<-SEEST+KKKK2
#Save simulation results in directory
write.csv(SEEST, "*****/seest41.csv")
}
```
